# Beyond wingtips: backbone alkylation affects the orientation of N-heterocyclic carbenes on gold nanoparticles

**DOI:** 10.1039/d5sc05986k

**Published:** 2026-01-12

**Authors:** Ahmadreza Nezamzadeh, Shayanta Chowdhury, Gaohe Hu, Nathaniel L. Dominique, Emmett Desroche, Sakiat Hossain, Mark D. Aloisio, Michael Furlan, Ryan R. K. Groome, Kayla Boire, Alastair B. McLean, Lasse Jensen, Jon P. Camden, Cathleen M. Crudden

**Affiliations:** a Department of Chemistry, Queen's University Kingston Ontario K7L 3N6 Canada r.nezam@queensu.ca cruddenc@chem.queensu.ca sakiat.hossain@queensu.ca 18mda1@queensu.ca 21kb56@queensu.ca; b Carbon to Metal Coating Institute, Queen's University Kingston Ontario K7L 3N6 Canada; c Queen's University, Department of Physics, Engineering Physics and Astronomy, Stirling Hall Kingston Ontario K7L3N6 Canada 15ed@queensu.ca groome.ryan@gmail.com michael.furlan@exeter.ox.ac.uk mcleana@queensu.ca; d Department of Chemistry and Biochemistry, University of Notre Dame Notre Dame Indiana 46556 USA schowdh2@nd.edu jon.camden@nd.edu ndominiq@alumni.nd.edu; e Institute of Transformative Bio-Molecules (WPI-ITbM), Nagoya University Chikusa Nagoya 464-8602 Japan; f Department of Chemistry, The Pennsylvania State University 104 Benkovic Building, University Park Pennsylvania 16802 USA gjh5278@psu.edu jensen@chem.psu.edu

## Abstract

The effect of wingtip groups on the orientation of N-heterocyclic carbene (NHC)-based self-assembled monolayers (SAMs) on a variety of metal surfaces has received considerable attention. However, the influence of backbone substituents on orientation has received virtually no attention, despite the fact that backbone interactions are critical for upright orientation of thiolate-based SAMs and that backbone functionalization is important for many applications. To address this question, a series of gold nanoparticles (NPs) supported by NHCs featuring symmetrical or asymmetrical long alkyl backbone substituents and ethyl and isopropyl wingtips were synthesized. The gold NPs were characterized using UV-vis spectroscopy, electron microscopy, mass spectrometry, and surface-enhanced Raman spectroscopy (SERS). Experimental SER spectra were compared to simulated spectra, illustrating that both ethyl and isopropyl NHCs with symmetrical dodecyl long chains in the backbone adopt a primarily vertical configuration on the gold surface. However, the ethyl NHC with a single hexyloxy backbone substituent adopts mainly a flat configuration on the gold NP surface based on combined SERS and scanning tunneling microscopy (STM) results. This is attributed to on-surface interactions between long alkyl chains, which provide an unanticipated source of stability favoring the flat-lying orientation. Lastly, the thermal stability of the NHC-functionalized gold NPs at elevated temperatures was investigated. The dodecyloxy-functionalized NHC AuNPs remain thermally stable for 72 hours at 100 °C, representing a significant improvement over state-of-the-art NHC-AuNPs. NHCs containing isopropyl wingtip groups provide NPs with higher levels of stability than diethyl-substituted NHCs, regardless of backbone substituents. Taken together, our results highlight critical synthetic considerations for NHC ligand design, enabling control of ligand orientation and nanomaterial stability by tuning NHC backbone substituents.

## Abstract 2

In addition to the strong effect of wingtip structure, the type and number of backbone groups on N-heterocyclic carbenes (NHCs) is shown to have a significant effect on the orientation of NHCs on gold nanoparticles and flat Au(111) surfaces.

## Graphical abstract

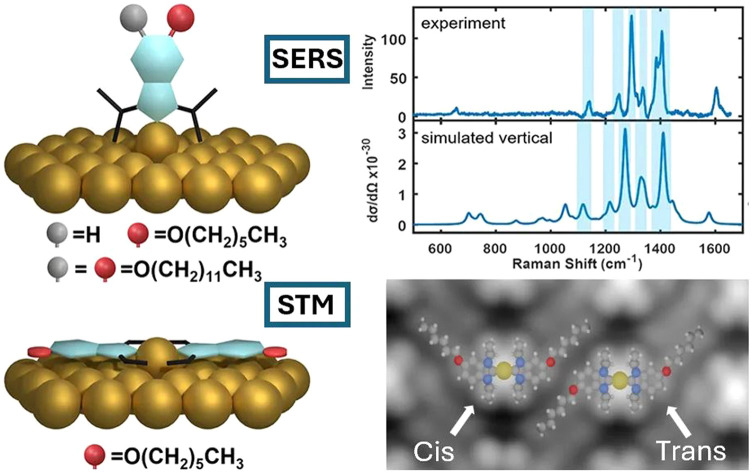

## Introduction

**Fig. 1:**
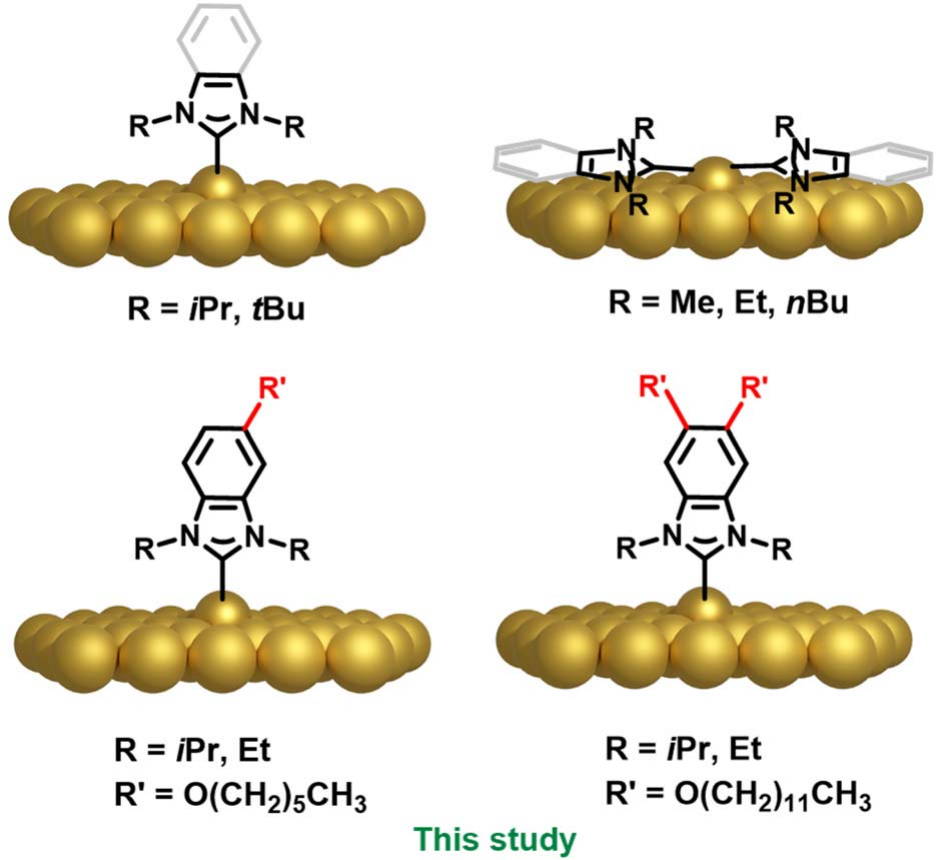
Predominant NHC orientation on gold surfaces and NPs as a function of NHC structure.

N-heterocyclic carbenes (NHCs) are increasingly being examined as alternatives to thiol ligands for surface functionalization due to their unique properties and strong binding affinity to a variety of metals.^1–6^ The structures, and therefore stabilities of NHC monolayers are heavily influenced by the wingtip groups attached to the NHC nitrogen atoms, which modulate steric interactions and affect surface packing and orientation.^7–13^ The binding geometries of NHCs on planar gold surfaces have been the focus of considerable study since Crudden, Baddeley *et al.* and Papageorgiu *et al.* independently demonstrated that NHCs with small wingtips form NHC_2_M-type species oriented parallel to the gold surface (Fig. 1).^9,12^ Subsequent work from Glorius,^10,13^ and Venkataraman,^8^ supported the conclusion that NHCs with small, primarily alkyl wingtip groups like methyl, ethyl and *n*-butyl take up a similar flat-lying geometry after metal atom abstraction. The isopropyl NHC is unique in that it typically positioned upright to the surface, but can show a tilt and evolve to flat lying species upon exposure to high heat.^8^ NHCs with *tert*-butyl wingtips exhibit a consistent upright, ad-atom bound configurations on coinage metals (Fig. 1).^11^ Other factors such as the temperature of deposition and annealing have been shown to play important roles.^7,14^

Importantly, upright geometries are frequently seen on step edges even for NHCs with Me substituents^11,12^ which likely complicates analysis of polycrystalline/highly faceted systems.^15,16^ The expectation of different orientations on step edges *vs.* terraces mean that understanding how NHCs bind to nanoparticle surfaces (NPs) may be highly complex.^8–13,17,18^

The Camden, Jenkins, and Jensen groups have pioneered the use of experimental and simulated surface-enhanced Raman spectroscopy (SERS) to provide information about NHC orientation on gold NPs.^13,19–23^ This technique provides important insights into how NHC ligands are arranged on NP surfaces, and how their orientation affects the electronic properties.^21,22^ SERS analyses suggest that NHCs with *i*Pr wingtips can take on both flat and upright orientations on the NP surface.^21^ NHCs with Me, Et, *tert*-butyl, and hexyl wingtip groups on spherical AuNPs most commonly adopt a flat configuration. However, a small fraction of vertically oriented carbenes is also observed on NP surfaces.^22^

Understanding the geometry of the NHC on these surfaces is of critical importance to predict stability, since flat-lying NHC_2_M complexes are more labile,^7,11^ and since the use of NHC-SAMs in sensing applications will be optimal with the NHC upright, exposing biorecognition elements to solution.^19,24–30^

This situation is not dissimilar from that observed in thiol-based SAMS, which often take up either flat-lying or upright conformations depending on surfactant density and thiol structure.^31–38^ The main structural element employed in thiol-based SAMs to enforce upright geometry is the presence of long alkyl chains, whose multiple van der Waals interactions are the critical driving force behind the formation of upright SAMs.^31,39–41^

While long alkyl chains have been introduced on NHC backbones,^42,43^ their effect in the context of NHC orientation has not been reported and it is unclear whether van der Waals interactions between neighbouring alkyl groups are possible because of the increased spacing of NHCs on planar metal surfaces compared with thiols. Herein, we describe the use of SERS combined with scanning tunneling microscopy (STM) to investigate the influence long-chain alkyl groups on the backbone of NHC ligands have on the orientation of NHCs on both NP and planar surfaces. Interestingly, we show that on-surface interactions between a single long alkyl chain on the backbone of an NHC provide an unanticipated source of stability favoring the flat-lying orientation. However, two alkyl chains are not well accommodated in the flat-lying conformation and cause even those NHCs known to exist primarily in the flat-lying configuration to assume an upright binding geometry. Considering the importance of NHC monolayer stability for real-life applications,^44–47^ we also assess the impact of these structural modifications on the thermal stability of the resulting NHC-stabilized NPs.

## Results and discussion

**Scheme 1:**
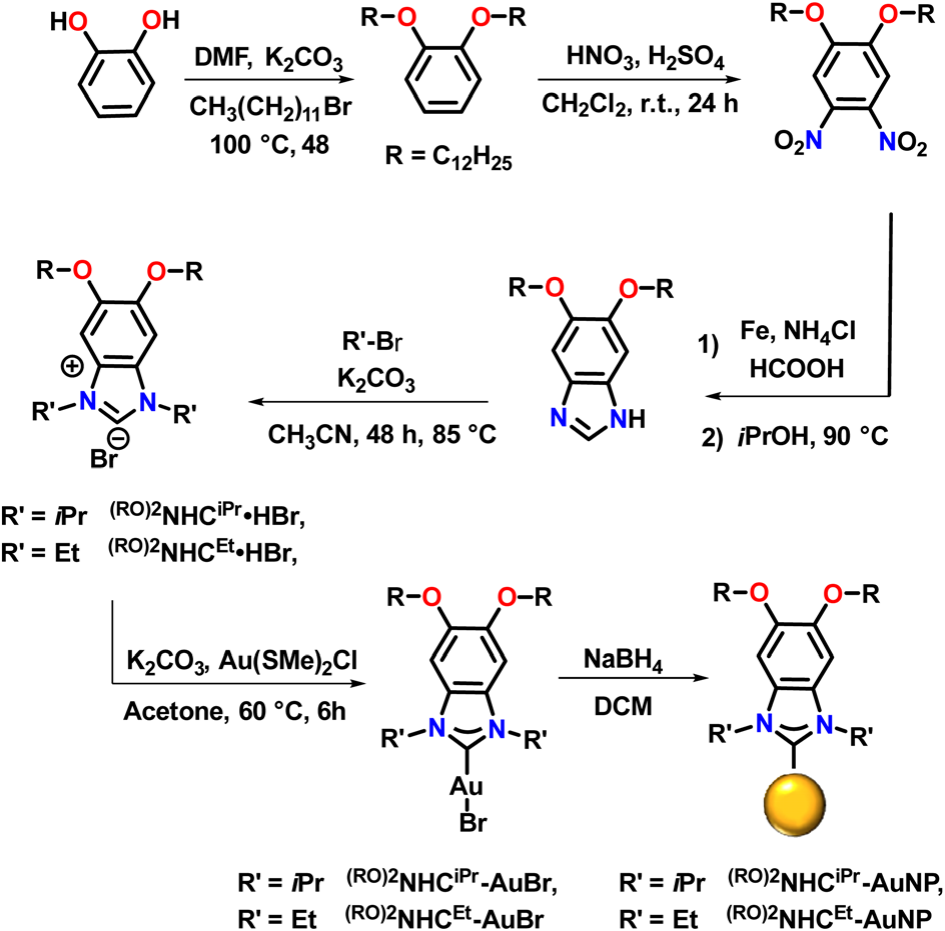
Schematic illustration of bottom-up strategy to fabricate AuNPs protected by NHCs.

We began by preparing benzannulated NHC precursors containing two alkyl chains on the backbone (Scheme 1). Catechol was first reacted with dodecyl bromide in the presence of K_2_CO_3_ in DMF at 100 °C to yield 1,2-bis(dodecyloxy)benzene. The resulting dialkylated product was then treated with a mixture of HNO_3_ and H_2_SO_4_ in dichloromethane to yield the corresponding dinitrobenzene, with the directing effects of the oxygen substituents controlling the placement of the nitro groups. Next, reduction with iron, followed by reaction with formic acid resulted in the formation of benzannulated imidazoles. Alkylation with the respective alkyl halides gave ^(RO)2^NHC^iPr^·HBr and ^(RO)2^NHC^Et^·HBr. In the next step, the Au complexes, ^(RO)2^NHC^iPr^-AuBr and ^(RO)2^NHC^Et^-AuBr, were prepared by reacting NHC bromide salts with ClAuSMe_2_ in the presence of K_2_CO_3_ in acetone at 60 °C (Scheme 1). The loss of signals attributable to the NC**H**N proton peak in the ^1^H NMR spectra and the appearance of C–Au signals in the ^13^C{^1^H} NMR spectra confirmed the successful synthesis of the desired Au(i) complexes (Fig. S34–S37 and S42–S45).

**Fig. 2:**
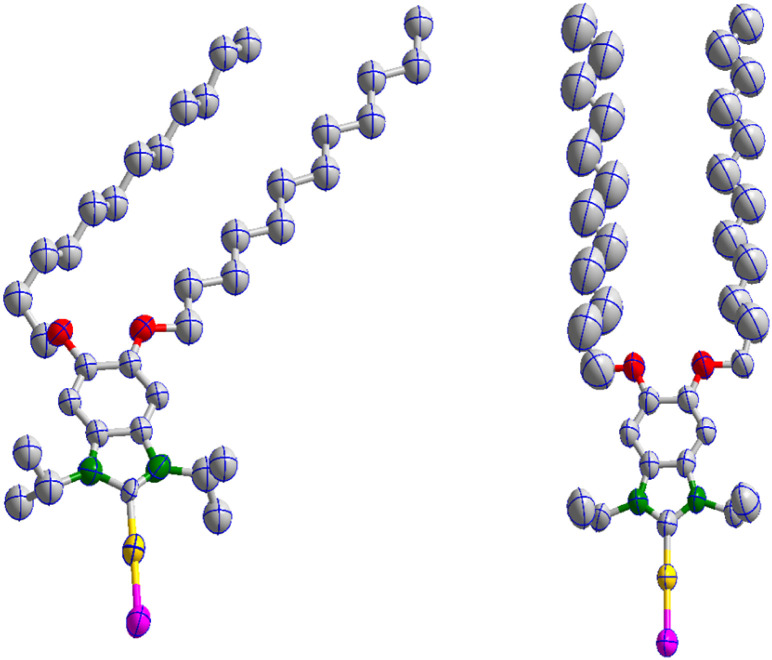
Molecular structures of ^(RO)2^NHC^iPr^-AuBr (left) and ^(RO)2^NHC^Et^-AuBr (right) with thermal ellipsoids shown at the 50% probability level. H atoms are omitted for clarity. For bond lengths (Å) and angles (deg) (Tables S2–S5) and other crystallographic data (Table S1), see the SI.

The ^(RO)2^NHC^iPr^-AuBr and ^(RO)2^NHC^Et^-AuBr complexes crystallize in monoclinic crystal systems with *P*2_1_/*c* and *P*2_1_/*n* space groups, respectively (Fig. 2). Single crystal X-ray diffraction studies reveal that for ^(RO)2^NHC^iPr^-AuBr, one –OC_12_H_25_ substituent lies in the same plane as the heterocyclic ring, while the other –OC_12_H_25_ chain is positioned above and below this plane. In case of ^(RO)2^NHC^Et^-AuBr, both OC_12_H_25_ substituents and the benzimidazole NHC backbone lie in a single plane. It is unclear whether this difference will have any impact on on-surface geometry.

Gold NPs were then prepared by a bottom-up route following published procedures with some modifications.^42,48^ To a solution of ^(RO)2^NHC^iPr^-AuBr and ^(RO)2^NHC^Et^-AuBr in dichloromethane (DCM), a freshly prepared solution of NaBH_4_ in ethanol was added (Scheme 1). This resulted in a colour change from pale yellow to dark red over a period of 20 hours. After this period, work-up and purification proceeded as described in the SI.

**Fig. 3:**
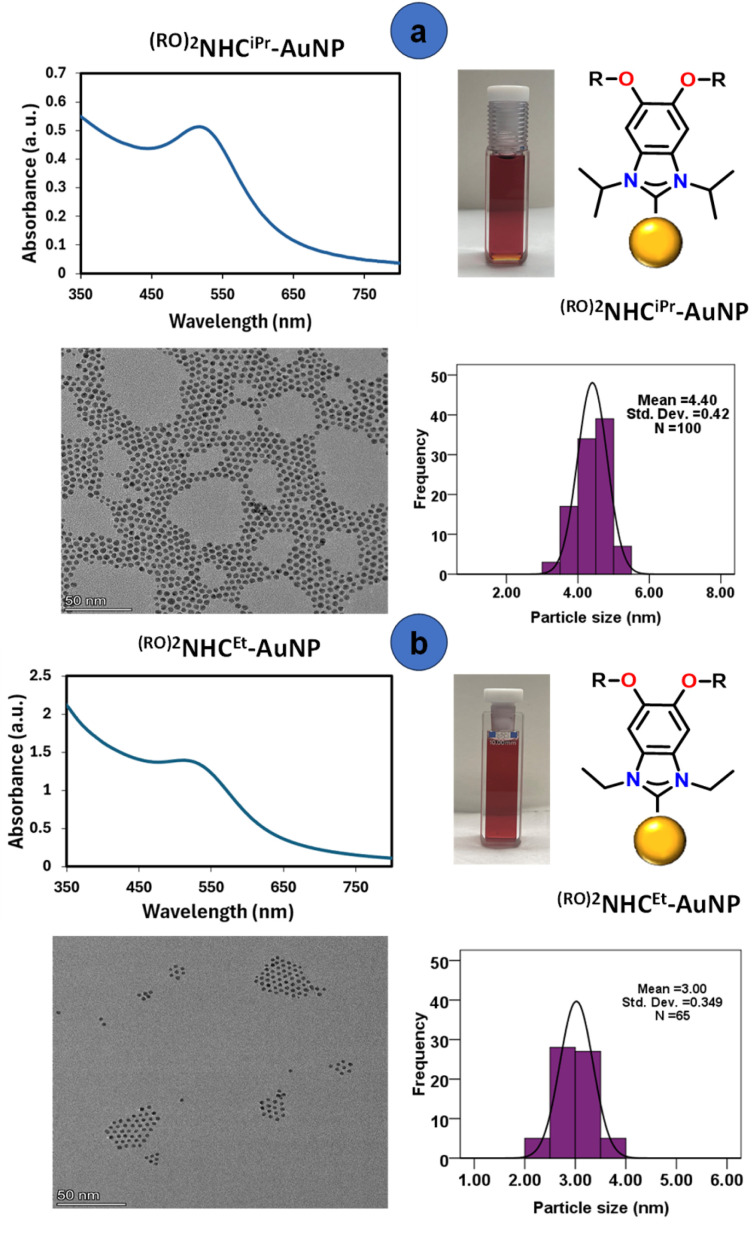
UV-vis and TEM images of bottom-up synthesized (a) ^(RO)2^NHC^iPr^-AuNP and (b) ^(RO)2^NHC^Et^-AuNP.

UV/vis spectra of the obtained NPs are characterized by a broad absorption peak around 530 nm, confirming the formation of Au NPs (Fig. 3). The NPs obtained from reduction of ^(RO)2^NHC^iPr^-AuNP were observed to have a stronger, more well defined plasmonic signal than those prepared from reduction of ^(RO)2^NHC^Et^-AuNP (Fig. 3). To measure the particle size of the obtained NPs, transmission electron microscopy (TEM) analysis was performed. This revealed the formation of highly uniform AuNPs with an average diameter of 4.4 ± 0.4 nm for ^(RO)2^NHC^iPr^-AuNP and 3.0 ± 0.4 nm for ^(RO)2^NHC^Et^-AuNP (Fig. 3a and b).

The presence of a covalent Au–C bond in ^(RO)2^NHC^iPr^-AuNP and ^(RO)2^NHC^Et^-AuNP was supported by mass spectrometry studies. Laser desorption ionization mass spectrometry (LDI-MS) showed prominent peaks at *m/z* = 1337.96 for ^(RO)2^NHC^iPr^-AuNP and *m/z* = 1281.93 for ^(RO)2^NHC^Et^-AuNP (Fig. S1). These peaks are widely agreed upon to correspond to the bis ion [(NHC)_2_Au]^+^, which has been previously observed in LDI-mass spectra of NHC-based SAMs on gold surfaces.^30,49–51^

**Fig. 4:**
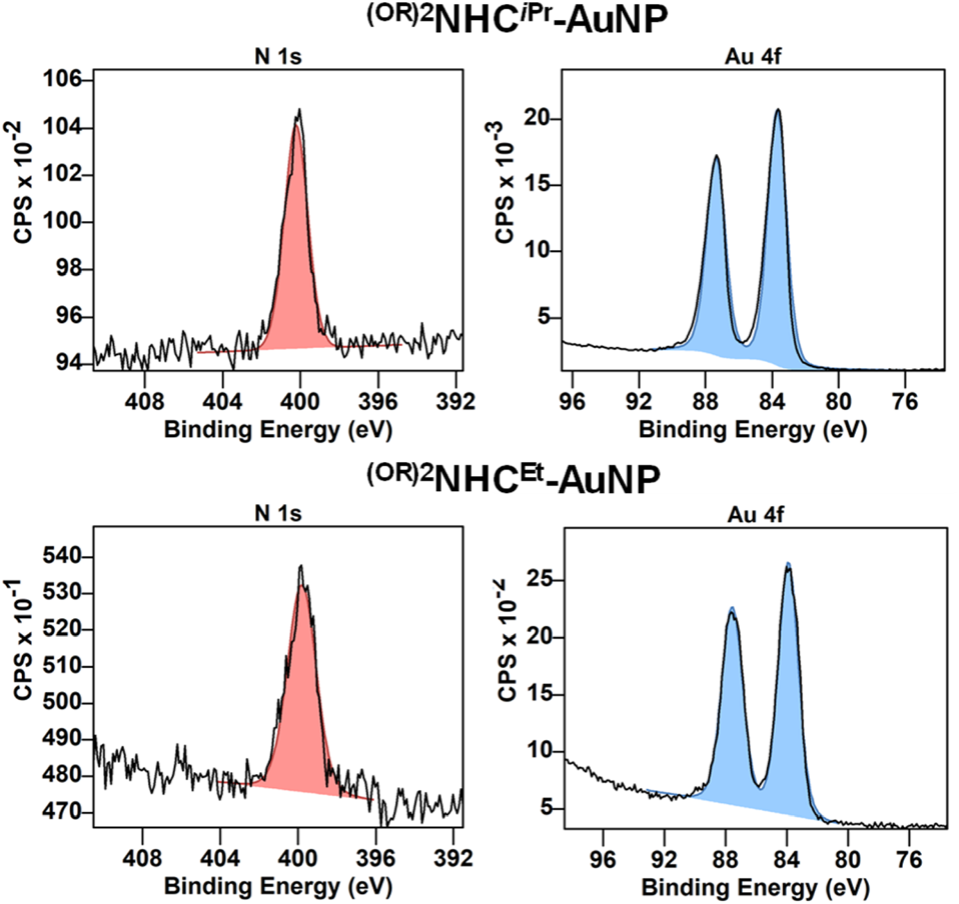
XPS spectra of bottom-up synthesized ^(RO)2^NHC^iPr^-AuNP (top) and ^(RO)2^NHC^Et^-AuNP (bottom) showing the N 1s and Au 4f regions.

X-ray photoelectron spectroscopic (XPS) measurements were also conducted to investigate the binding of NHCs to the surface of the AuNPs. For ^(RO)2^NHC^iPr^-AuNP and ^(RO)2^NHC^Et^-AuNP, the C 1s spectra were deconvoluted into three components (Fig. S4 and S5): C–C aliphatic and C=C aromatic heterocycle (284.7 eV), C–N bond (285.9 eV), and C–O bond (286.5 eV).^48,52^ The N 1s signal was characterized by a symmetric peak at 400.2 and 399.9 eV for ^(RO)2^NHC^iPr^-AuNP and ^(RO)2^NHC^Et^-AuNP, respectively (Fig. 4). These peaks were shifted towards weaker binding energies in comparison to those obtained for ^(RO)2^NHC^iPr^-AuBr (401.0 eV) and ^(RO)2^NHC^Et^-AuNP (400.9 eV) (Fig. S2 and 3). This type of shift is consistent with previous reports of NHCs on metallic NPs and NHCs on planar gold surfaces.^25,52,53^ As expected, Br 3d_5/2_ and 3d_3/2_ signals observed for ^(RO)2^NHC^iPr^-AuBr and ^(RO)2^NHC^Et^-AuBr at ∼68.0 eV, and were absent after the formation and purification of the AuNPs.^53^ This absence indicates the complete reduction of the Au complexes to NPs and indicates no contamination by bromide.^53^ The clean reduction is also supported by the XPS Au 4f signal, which is composed of only two peaks at 84.0 eV and 87.6 eV, as expected for the Au 4f_7/2_ and 4f_5/2_ contributions (Fig. 4). The energy shift between both components, measured at 3.6 eV, is characteristic of Au(0).^53^ These data show that the surface of the AuNPs has an overall oxidation state close to zero, and that any Au(i) species present are below the limit of detection.^25,54^

Thermogravimetric analysis (TGA) of bottom-up synthesized NPs was employed to assess the thermal stability of the NPs (Fig. S7 and S9). The compositions of the NP samples were determined to be 35 wt% and 39 wt% of organic surface components for ^(RO)2^NHC^iPr^-AuNP and ^(RO)2^NHC^Et^-AuNP, respectively, with the remaining 65 wt% and 61 wt% corresponding to the Au core. By comparing the organic content obtained from TGA with the NP diameters measured by TEM, the average number of NHC ligands per NP was estimated using the method reported by Johnson *et al.*^55^ The calculations indicate that each ^(RO)2^NHC^iPr^-AuNP and ^(RO)2^NHC^Et^-AuNP contains approximately 497 and 194 NHC ligands per NP, respectively. It should be noted that these values are approximate, as the method assumes uniform ligand coverage and idealized particle geometry.

### Surface-enhanced Raman spectroscopy (SERS)

Having shown that the reduction of complexes ^(RO)2^NHC^iPr^-AuBr and ^(RO)2^NHC^Et^-AuBr results in the clean formation of NHC-functionalized nanoparticles, we then examined the orientation of the NHC ligand on the NP surface through surface enhanced Raman spectroscopy (SERS). Experimental SERS measurements were supported by simulations of the Raman spectra carried out using density functional theory (DFT) with the Amsterdam density functional (ADF) engine from the Amsterdam modelling suite 2024.^56,57^ Raman intensities were calculated as squared polarizability derivatives with respect to normal mode displacement.^58,59^ Typically, an Au_58_ cluster is chosen to simulate the SERS of NHCs on NPs, which provides sufficient space to accommodate the wingtips in a flat NHC configuration.^22^ To reduce the computational cost of simulating SERS of NHCs taking upright configuration, we opted to simplify the model to an Au_2_ cluster. For this model, the molecule-cluster axis was aligned with the *z*-axis and the *zz* component of polarizability derivative was chosen to effectively simulate the SERS spectra of upright NHC ligands.^58,59^ A comparison of the Au_2_ and the Au_58_ cluster (Fig. S10) indicates that the SER spectra of the upright configuration can be effectively modelled in this way.

**Fig. 5:**
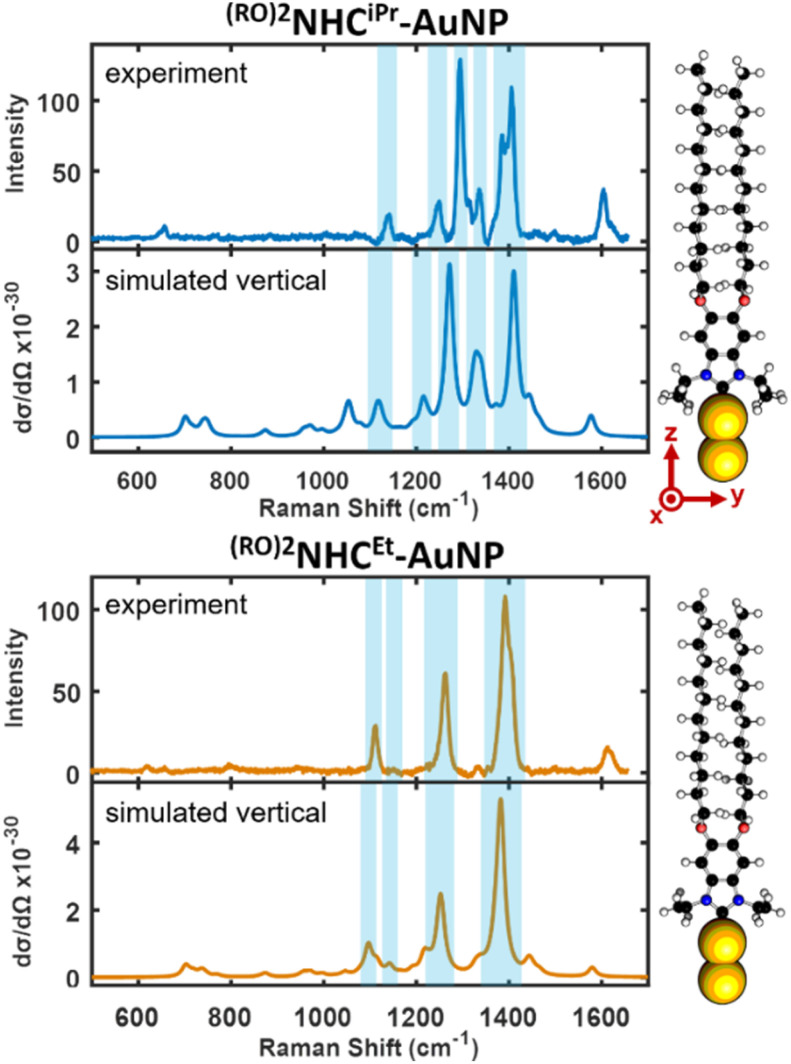
Experimental and simulated SER spectra of bottom-up synthesized ^(RO)2^NHC^iPr^-AuNP (top) and ^(RO)2^NHC^Et^-AuNP (bottom).

Previous reports have shown that a combination of the simulated spectra of flat-lying and upright species captures the main features of the experimental SER spectra of NHCs on NPs.^21,22^ In Fig. 5 we show experimental and simulated SER spectra for ^(RO)2^NHC^iPr^-AuNP and ^(RO)2^NHC^Et^-AuNP. The *y*-axis of the simulated spectra provides the calculated Raman cross-section arising from the model used.^21,22^ For both systems, the spectroscopic features observed experimentally correlate well with the vertical SERS simulation, despite the presence of primary alkyl wingtips in ^(RO)2^NHC^Et^-AuNP that would be expected to lead to flat-lying species. For example, the dominant peaks in the experimental spectra of ^(RO)2^NHC^iPr^-AuNP appear at ∼1295 and ∼1406 cm^−1^ and correlate well to the dominant peaks in the simulated spectrum at ∼1270 and ∼1411 cm^−1^, respectively. Likewise, the dominant peaks in the experimental spectrum for ^(RO)2^NHC^Et^-AuNP appear at ∼1113, ∼1262, and ∼1392 cm^−1^ and correlate well with the dominant peaks in the simulated spectrum at ∼1096, ∼1253, and ∼1382 cm^−1^, respectively.

In each case, the spectroscopic signatures for the experimental SERS are in excellent agreement with the simulated vertical spectral signatures. On the *y*-axes, experimental intensity is in units of counts mW^−1^ s^−1^ and theoretical Raman cross section d*σ*/d*Ω* is in units of cm^2^ sr^−1^.

To provide more insight into the NHC geometry on the nanoparticle surfaces, we also prepared NHC-stabilized nanoparticles using a top-down approach. This approach allows us to use larger nanoparticle scaffolds, which will have greater surface enhancement during the SERS experiments and to investigate the impact of the NP preparation method on the resulting materials. The top-down NPs are synthesized according to the method of Camden and Jenkins.^28^ Briefly, 20 nm Lee and Meisel-type^60^ gold colloids are synthesized in water (TEM analysis Fig. S11a) according to standard procedures.^30,61^ Next, these citrate-capped AuNPs were treated with a solution of 10 mM ^(RO)2^NHC^iPr^-AuBr or ^(RO)2^NHC^Et^-AuBr gold complex in dichloromethane (1 µL of complex for every 1 mL of gold nanoparticles). These AuNPs are still able to be dispersed in water.

TEM analysis of these particles shows almost no change in particle size after ligand exchange (Fig. S11b and c). To confirm the presence of NHCs on the surface of the NPs, XPS measurements were performed. Similar to the bottom-up synthesized NPs, a symmetric N 1s peak at approximately ∼400.0 eV was observed, confirming the successful binding of NHCs to the NP surface (Fig. S12). In addition to the expected signals, the C 1s spectrum showed additional peaks at ∼287.7 and ∼288.5 eV, corresponding to C=O and O–C=O species, respectively, indicating the presence of citrate on the NP surface.

**Fig. 6:**
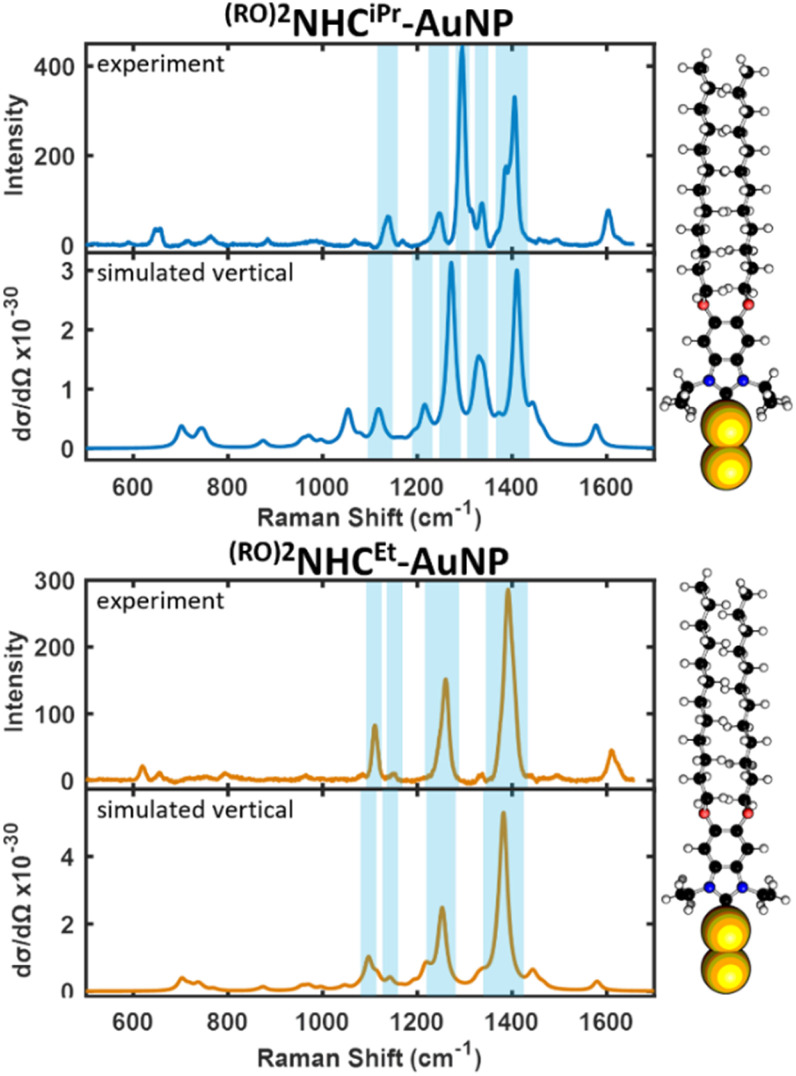
Experimental and simulated SER spectra of top-down synthesized ^(RO)2^NHC^iPr^-AuNP (top) and ^(RO)2^NHC^Et^-AuNP (bottom).

The NPs were then aggregated using sodium bromide to maximize SERS intensity, and spectra were acquired for the NP aggregates. The resulting SER spectra (Fig. 6) illustrate that both bottom-up and top-down protocols result in identical SERS signatures, suggesting that ligand orientation is agnostic to the method of NHC introduction. Prior work has also shown no discernible change in SER spectra of NHC-functionalized AuNPs when NPs between 20 and 100 nm were examined.^28^ However, the NPs obtained from the top-down approach exhibit spectra with higher intensity, enabling the detection of weaker bands in the 1100–500 cm^−1^ range. As the experimental spectra are dominated by features attributed to vertically oriented carbenes, we can infer that the population of the flat species is not significant. This is interesting because previous SERS studies on NP surfaces show that while NHCs with *i*Pr wingtips adopt a combination of vertical and flat orientations, NHCs with Et wingtips most commonly adopt a flat-lying orientation.^21,22^ STM studies on planar surfaces concur that NHCs with Et wingtip groups and no backbone functionalization adopt a flat-lying NHC_2_M species on Au(111) terraces.^12^ While we cannot exclude the possibility of a minority population of flat-lying species at the NP surface, it is clear from our SERS studies that for these nanoparticles, there is a high population of the NHC in a vertical orientation. As we will show subsequently, we attribute this to the presence of the two backbone substituents.

The strong SERS modes that arise from the vertical orientation of the NHC are highlighted in blue, as interpreted by their respective simulated spectra. On the *y*-axes, experimental intensity is in units of counts mW^−1^ s^−1^ and theoretical Raman cross section d*σ*/d*Ω* is in units of cm^2^ sr^−1^.

**Scheme 2:**
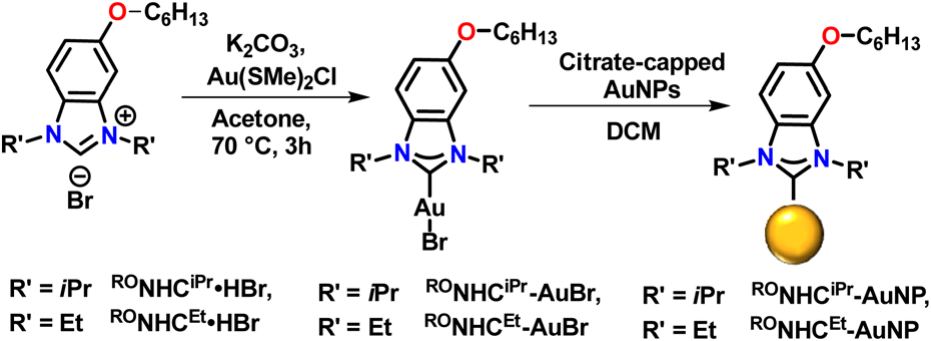
Synthesis of ^RO^NHC^iPr^-AuNP and ^RO^NHC^Et^-AuNP using top-down approach.

**Fig. 7:**
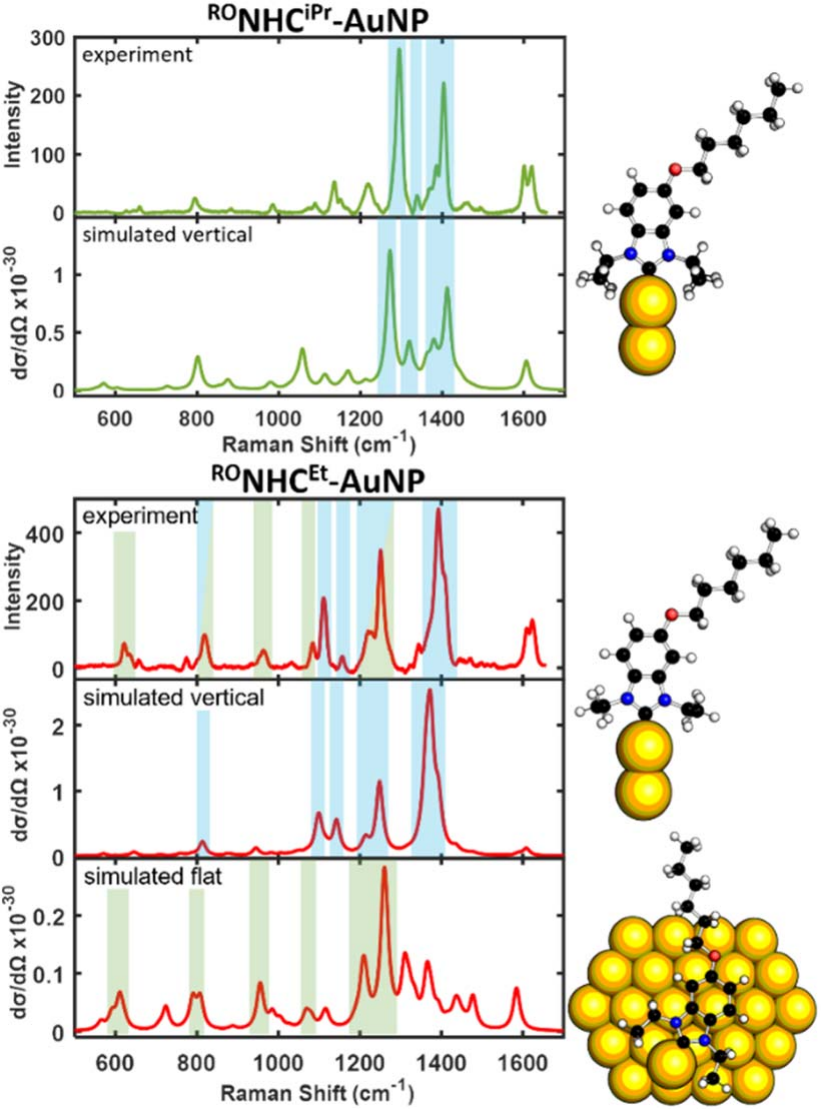
Experimental and simulated SER spectra of top-down synthesized ^RO^NHC^iPr^-AuNP (top) and ^RO^NHC^Et^-AuNP (bottom).

To test this hypothesis, we prepared two additional NHC precursors: ^RO^NHC^iPr^·HBr and ^RO^NHC^Et^·HBr, that have a single alkyl chain on the backbone (Scheme 2). Gold complexes of these NHCs (^RO^NHC^iPr^-AuBr and ^RO^NHC^Et^-AuBr) were prepared by the same method used to prepare ^(RO)2^NHC^iPr^-AuBr and ^(RO)2^NHC^Et^-AuBr. NPs functionalized by these complexes were then prepared *via* the top-down approach and the resulting NPs characterized by XPS (Fig. S15) and utilized in a SERS study (Fig. 7).

For ^RO^NHC^iPr^-AuNP, the observed spectrum is again well described by the vertical simulated spectrum. However, for ^RO^NHC^Et^-AuNP, which bears Et wingtips, we find that the sub ∼1100 cm^−1^ peaks become significantly more intense (*e.g.* bands at ∼621, ∼963, and ∼1084 cm^−1^ are examples of such peaks and are highlighted in green). In a previous SERS study of NHC orientation,^22^ peaks in this region were attributed to flat lying NHCs.

Given the potential importance of the flat configuration and the inability of the Au_2_ model to accurately simulate these flat NHCs (Fig. S10), an Au_58_ cluster was used to calculate the SERS spectra of a flat-lying ^RO^NHC^Et^. Consistent with previous literature, only the polarizability component perpendicular to the metal surface was used to simulate the SERS spectra and the resulting flat Raman cross-sections were several orders of magnitude lower than the vertical cross-sections.^21,22^ This distinction is crucial, as it implies that if the flat simulated SERS modes appear in the experimental spectra to any extent, the population of flat NHCs is vastly higher (>90%) than that of the vertical species. These results, taken together, are best explained by the presence of a significantly larger population of the flat-lying species in the case of ^RO^NHC^Et^-AuNP and provide strong evidence that the nature of backbone substitution affects orientation.

The strong SERS modes that arise from the vertical orientation of the NHC are highlighted in blue, while the modes arising from the flat orientation of the NHC are highlighted in green, as interpreted by their respective simulated spectra. On the *y*-axes, experimental intensity is in units of counts mW^−1^ s^−1^ and theoretical Raman cross section d*σ*/d*Ω* is in units of cm^2^ sr^−1^.

### STM studies

**Fig. 8:**
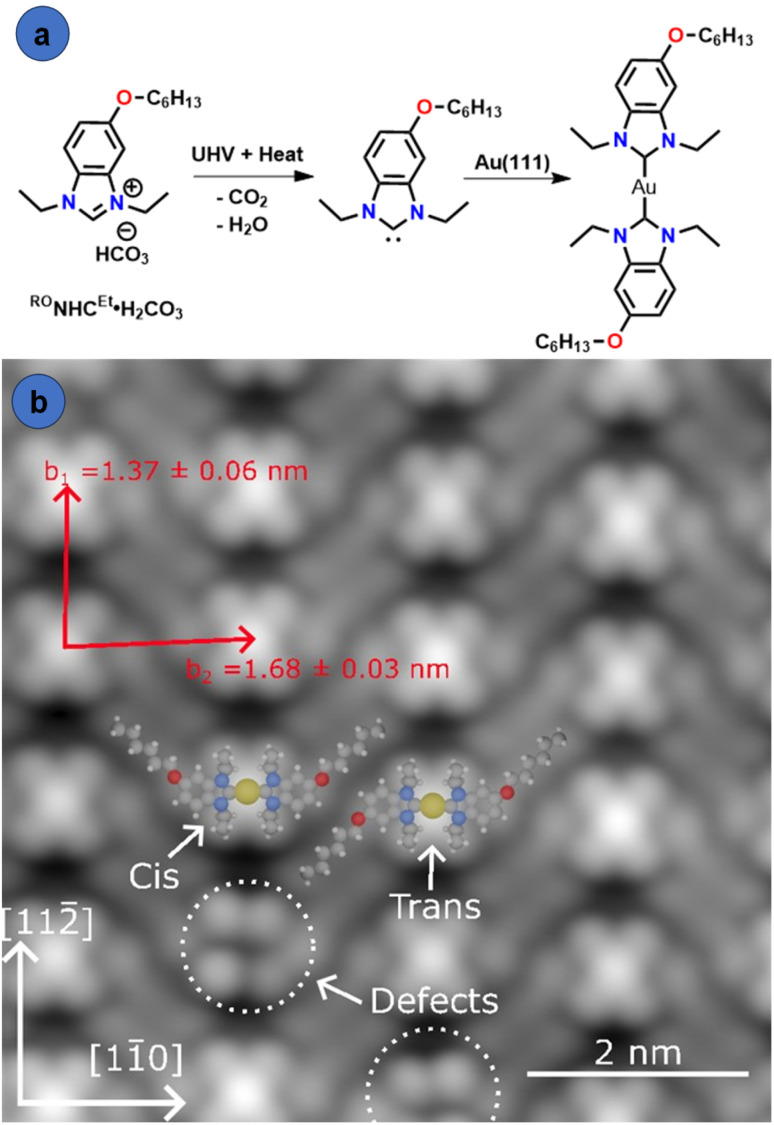
(a) Schematic representation of ^RO^NHC^Et^·H_2_CO_3_ deposition on Au(111), and (b) STM image of the ^RO^NHC^Et^ overlayer on Au(111) (7 nm × 7 nm, 20 pA, 0.100 V, 77.08 K). The rectangular unit cell describing the overlayer structure is overlaid in red. Molecular models are overlaid, illustrating the surface-bound cis and trans isomers. On-surface interactions between alkyl chains play an important, and previously unexplored role in guiding surface organization.

To provide greater insight into the orientation of ^RO^NHC^Et^, we examined SAMs of ^RO^NHC^Et^ on single crystalline Au(111) surfaces by scanning tunneling microscopy, STM. We began by preparing ^RO^NHC^Et^·H_2_CO_3_ (R = *n*-hexyl Fig. 8a) and depositing this NHC onto an atomically flat Au(111) surface in a vacuum chamber attached to the STM. As shown in Fig. 8b, microscopy of the overlayer, performed at 77 K with constant-current, clearly resolves single molecules within the overlayer. These images are best explained by the formation of flat-lying bis-(NHC)_2_Au complexes from ^RO^NHC^Et^, in agreement with previous observations of NHCs with primary alkyl groups on planar surfaces and with our SERS studies.^9,12,13^

These NHC complexes are found to self-assemble into a rectangular pseudo-lattice. The assembly is best described by an approximate unit cell with parameters ‖*b*_1_‖ = 1.32 nm and ‖*b*_2_‖ = 1.70 nm, corresponding to the adatom positions (Fig. 8b) and resulting in an overlayer density of 0.9 NHCs nm^−2^. The STM images also show resolved Et wingtips, benzimidazole backbone and alkyl chains of the NHC complexes. This high-resolution imaging reveals that the assembly is composed of columns of cis and trans isomers of NHC complexes, as indicated by the overlaid models in Fig. 8b. Within the domains observed, the isomers alternate randomly along the [11̄0] direction (Fig. S16a), causing small variations in the nearest neighbour distances of adjacent Au adatoms. Along with the persisting herringbone reconstruction of the Au(111) surface (Fig. S16b), this prevents the assignment of an exact lattice model. Also observed in Fig. 8b are local defects that do not disrupt the order of the self-assembly pattern, indicated by dashed circles. They appear similar to the bis-complexes but lack the bright central protrusion. As such, these are thought to be surface bound NHCs configured to form a bis-complex, but lacking the Au adatom. The imaged domains consist of roughly equal portions of both isomers, suggesting these are energetically equivalent. While alternative lattices consisting of only cis or trans isomers are feasible, none are observed experimentally under the conditions described. The observed self-assembly pattern may thus imply a substantial energy barrier to switching between isomers, or, more likely, that this configuration forms to maximize molecular density and attractive van der Waals interactions between adjacent alkyl chains. These observations demonstrate that ^RO^NHC^Et^ adsorbs in a flat-lying geometry on the Au(111) surface in full agreement with SERS studies.

To determine the influence of changing the alkyl group (R) from dodecyl to hexyl, we also prepared ^RO^NHC^Et^·H_2_CO_3_ (R = *n*-dodecyl), and deposited it onto Au(111) as described above. Imaging of the resulting surfaces showed large areas with flat-lying NHC_2_Au species (Fig. S17), similar to what is shown in Fig. 8. However, unlike the *n*-hexyl system shown in Fig. 8, the dodecyl groups do not have as high a degree of order. Interactions between flat-lying alkyl chains from adjacent molecules and with the Au(111) surface likely contribute to the stability of the flat lying geometry. NHCs with two alkyl chains on the backbone such as ^(RO)2^NHC^Et^ would appear incapable of forming these types of strongly constrained surface-bound lattices, which may explain the observation of upright binding for these NHCs.

### Thermal properties

**Fig. 9:**
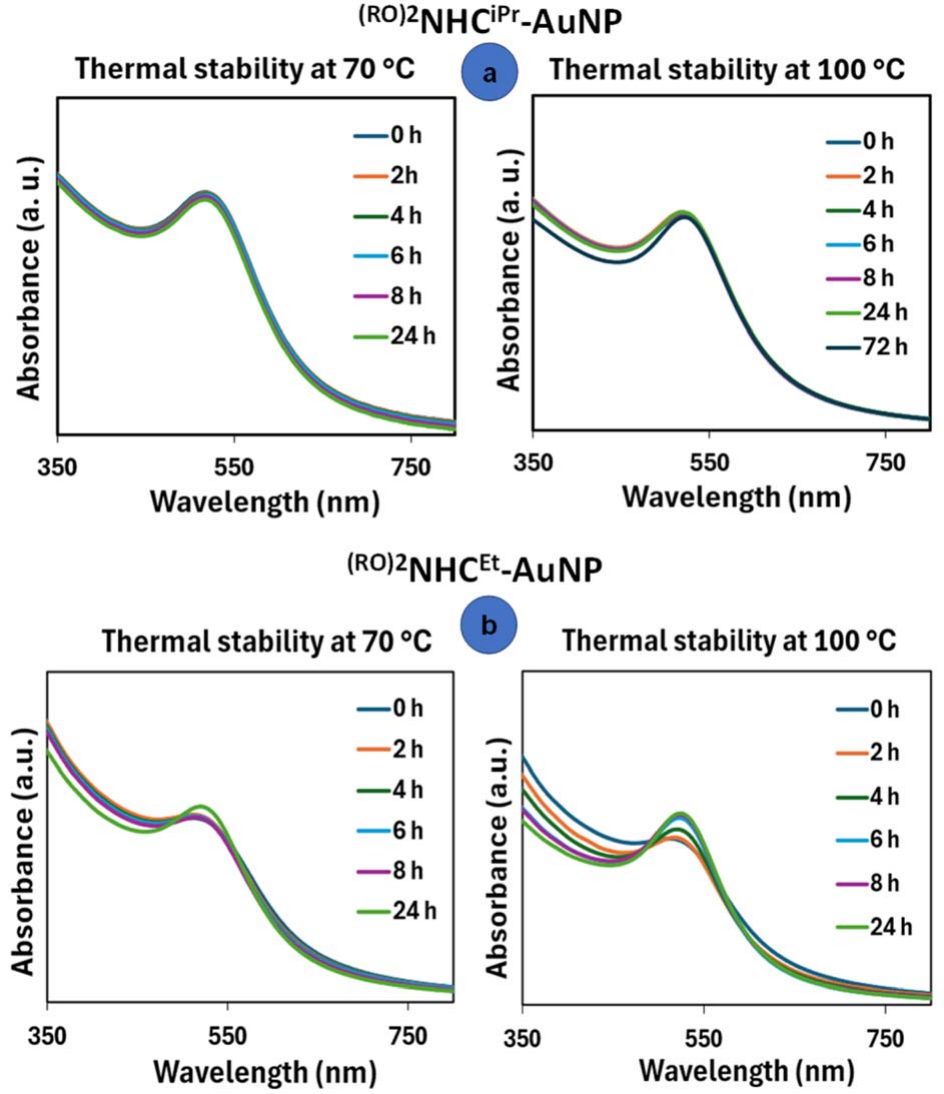
Thermal stability of bottom-up synthesized ^(RO)2^NHC^iPr^-AuNP (top) and ^(RO)2^NHC^Et^-AuNP (bottom) at 70 and 100 °C, R = *n*-dodecyl.

With a collection of NHC-protected NPs in hand, next we investigated the influence of ligands and synthetic procedures on the thermal stability of the resulting NPs. For each preparation, around 1 mg of the NP sample was dissolved in 3 mL of *m*-xylene and heated at 70 and 100 °C for varying durations of time. Changes in the NP composition were assessed by monitoring absorption changes *via* UV-vis spectroscopy. ^(RO)2^NHC^iPr^-AuNP (R = *n*-dodecyl) exhibited no signs of decomposition after heating at 100 °C for 24 hours, with only a minor sharpening of the SPR signal noted when the heating time was extended to 72 hours (Fig. 9a). TEM images (Fig. S18–S21) taken before and after thermal treatment of ^(RO)2^NHC^iPr^-AuNP showed consistently no changes in particle diameter within experimental error (from 4.4 ± 0.4 nm to 5.0 ± 0.4 nm). A similar experiment was carried out to assess the stability of ^(RO)2^NHC^Et^-AuNP (R = *n*-dodecyl). After heating the sample at 100 °C for 24 hours, we observed a sharpening of the SPR signal, indicating NP growth without any shift in position (Fig. 9b). The particle size increased from 3.0 ± 0.3 nm to 6.7 ± 0.7 nm (Fig. S22–S25), indicating lower stability even though SERS studies did not show significant spectroscopic differences between these two ligands.

To compare the effects of backbone and wingtip substituents on the thermal stability of NPs, we next examined ^(RO)2^NHC^iPr^-NP, ^(RO)2^NHC^Et^-NP (R = *n*-dodecyl), ^RO^NHC^iPr^-NP, and ^RO^NHC^Et^-AuNP (R = *n-*hexyl) synthesized *via* the top-down method. Since these NPs were soluble in aqueous media, a 1 : 1 mixture of ethylene glycol and water was used to raise the boiling point above 100 °C. The NHC-AuNPs solutions were then heated at 70 and 100 °C for varying durations. Fig. S26 presents the UV-vis spectra recorded at 70 °C, which show minimal changes in SPR peaks over 48 hours. TEM images (Fig. S27) taken after 48 hours also revealed no significant changes in size distribution. Likewise, the initial and final colours of the NHC-appended AuNP samples (Fig. S28a–c) exhibited no visible differences.

**Fig. 10:**
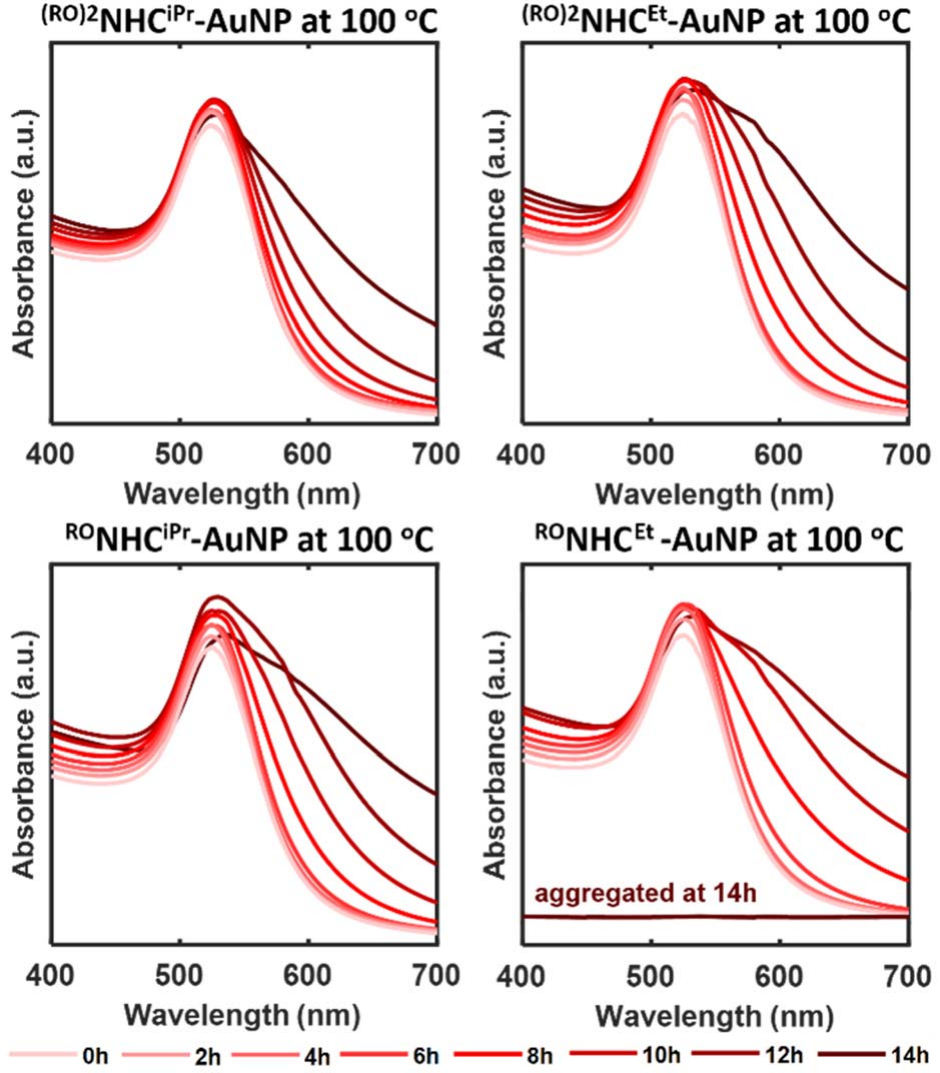
Thermal stability of top-down synthesized ^(RO)2^NHC^iPr^-, ^(RO)2^NHC^Et^-, ^RO^NHC^iPr^-, and ^RO^NHC^Et^-AuNPs at 100 °C.

At 100 °C, however, stability differences emerged (Fig. 10). Among the samples, ^(RO)2^NHC^iPr^-AuNP (R = *n*-dodecyl) demonstrated the highest resistance to aggregation, indicated by minimal peak broadening and less pronounced colour changes (Fig. 10 and S28). In addition, NPs synthesized using the bottom-up method showed higher stability (72 h) than those prepared by the top-down method (18 h). The lower stability of top-down NPs might be attributed to their larger size and the presence of residual citrate ligands on their surface.

If the wingtip is changed to a primary substituent, as in ^(RO)2^NHC^Et^-NP (R = *n*-dodecyl), or a single alkyl substituent is employed, as in ^RO^NHC^iPr^-AuNP (R = *n*-hexyl), greater broadening and deeper colour changes were observed compared to ^(RO)2^NHC^iPr^-AuNP, suggesting lower stability (Fig. 10 and S28). All three NPs eventually aggregated within 18 hours at 100 °C. Finally, ^RO^NHC^Et^-AuNP, with a single *n-*hexyl group and a primary wingtip, showed the lowest stability and aggregated after 14 hours, as evidenced by colour changes at 10, 12, and 14 hours (Fig. S28d–f).

These findings highlight the influence of NHC wingtip and backbone substituents on the thermal stability of the NPs they protect. Interestingly, NPs stabilized by ^(RO)2^NHC^iPr^ and ^RO^NHC^iPr^, featuring isopropyl wingtips, exhibited greater stability than NPs stabilized by NHCs with ethyl wingtips (^(RO)2^NHC^Et^ and ^RO^NHC^Et^). Similarly, NHCs with two alkyl chains, ^(RO)2^NHC^iPr^ and ^(RO)2^NHC^Et^, (R = dodecyl), showed enhanced resistance to aggregation compared to ^RO^NHC^iPr^. These data confirm that the primary factor contributing to thermal stability is the wingtip effect, but that carefully designed backbone functionalization contributes to stability. Clearly, proper selection of both wingtips and long-chain alkyl backbones can substantially enhance the stability of NHC-protected NPs.

Finally, the surface charge of the top-down synthesized AuNPs was evaluated by zeta potential measurements, which showed values of approximately −30 ± 5 mV for all samples (Fig. S30–S33), indicating good colloidal stability in water.^62^ Because of the hydrophobic nature of the bottom-up synthesized AuNPs, we could not carry out this measurement on these samples.

## Summary and conclusions

To conclude, the effect of backbone substituents on the orientation and thermal stability of NHC-stabilized gold nanoparticles is complex and significant. Ligand orientation was assessed using SERS, and experimental spectra were compared to simulated spectra to differentiate between vertical and flat-lying NHC orientations as two extremes. The resulting data illustrate that installing two hydrophobic dodecyloxy chains on the backbone results in primarily vertical configuration for NHCs with small wingtip groups on NPs, even when NHCs without such backbone groups have a strong preference for the flat-lying configuration. However, NHCs with a single alkyl group on the backbone and primary substituents on nitrogen were found by SERS and STM to take up predominantly flat-lying configurations. This suggests that the nature of backbone alkylation can disrupt or enhance the preference for flat-lying orientations. Importantly, if substituents can intercalate sufficiently on the surface, as in the single alkylated systems, the flat-lying orientation is preferred. If two groups are introduced on the backbone, SERS data show that even NHCs with a strong preference for the flat lying configuration will primarily adopt an upright configuration.

The thermal stability of NHC-stabilized nanoparticles was also assessed using UV-vis spectroscopy. Our results illustrate that NHCs bearing two dodecyloxy chains, which result in NHCs binding in an upright configuration, produce robust nanoparticles that remain stable even after 72 hours of continuous heating at 100 °C. This is a considerable stability improvement compared to past NHC architectures. Regardless of the backbone substituents, NHCs containing isopropyl substituents produce more stable NPs. These results add important new information to enable the control of NHC ligand orientation and generation of thermally stable nanoparticles.

## Author contributions

A. N. synthesized all the NHC ligands used for this study. Synthesized and purified NHC protected gold NPs using a bottom-up approach. Performed XPS, UV, TEM, and thermal stability measurements, followed by corresponding evaluations. Wrote, edited, and created figures for the manuscript. S. C. and N. L. D. synthesized gold NPs using a top-down approach. Performed SERS and TEM measurements, followed by corresponding evaluations. Wrote, edited, and created figures for the manuscript. G. H. performed DFT computations and evaluation. E. D. performed STM measurements followed by corresponding evaluations. Wrote, and edited the manuscript. S. H. grew single crystals of gold complexes and performed X-ray analysis and evaluation. Wrote and created figures for the manuscript. M. F. and R. G. performed STM measurements. M. A. synthesized ^HexO^NHC^Et^ bicarbonate salt. K. B. assisted in the synthesis of NHC ligands. C. M. C, J. P. C., L. J., and A. B. M. performed conceptualization, supervision, editing, and reviewing the manuscript and data analysis/interpretation.

## Conflicts of interest

There are no conflicts to declare.

## Supplementary Material

SC-017-D5SC05986K-s001

SC-017-D5SC05986K-s002

## Data Availability

CCDC 2410266 and 2410267 contain the supplementary crystallographic data for this paper.^63*a*,*b*^ The data supporting this article have been included as part of the supplementary information (SI). Supplementary information: synthetic protocols, characterization data, nanoparticle characterization, crystallographic data, and DFT calculations. See DOI: https://doi.org/10.1039/d5sc05986k.
