# Supplementary material for: Beyond wingtips: backbone alkylation affects the orientation of N-heterocyclic carbenes on gold nanoparticles
Source: Chem Sci. 2026 Jan 12;17(13):6717–26. doi: 10.1039/d5sc05986k (PMC12892494; doi:10.1039/d5sc05986k)
Supplement: SC-017-D5SC05986K-s001 [file SC-017-D5SC05986K-s001.pdf]

Supplementary Information (SI) for Chemical Science.
This journal is © The Royal Society of Chemistry 2026

# Supporting Information

# Beyond Wingtips: Backbone Alkylation Affects the Orientation of N-Heterocyclic Carbenes on Gold Nanoparticles

Ahmadreza Nezamzadeh[a,b], Shayanta Chowdhury[d], Gaohe Hu[f], Nathaniel L. Dominique[d], Emmett Desroche[c], Sakiat Hossain[a,b], Mark D. Aloisio[a,b], Michael Furlan, Ryan Groome, , Kayla Boire[a], Alastair B. McLean[c,]*, Lasse Jensen[f,]*, Jon P. Camden[d,]*, and Cathleen M. Crudden[a,b,e,]*

[a] Department of Chemistry, Queen's University, Kingston, Ontario K7L 3N6, Canada
[b] Carbon to Metal Coating Institute, Queen's University, Kingston, Ontario K7L 3N6, Canada
[c] Queen's University, Department of Physics, Engineering Physics and Astronomy, Stirling Hall, Kingston, Ontario, Canada.
[d] Department of Chemistry and Biochemistry, University of Notre Dame, Notre Dame, Indiana 46556, United States
[e] Institute of Transformative Bio-Molecules (WPI-ITbM), Nagoya University, Chikusa, Nagoya 464-8602, Japan
[f] Department of Chemistry, The Pennsylvania State University, 104 Benkovic Building, University Park, Pennsylvania 16802, USA

## S1. Contents

S2. Computational details ........ 3
S3. Experimental Section ........ 3
S.3.1. Reagents ........ 3
S.3.2. Piranha solution safety ........ 3
S.3.3. Characterization Methods ........ 4
S.3.3.1. NMR ........ 4
S.3.3.2. UV−vis ........ 4
S.3.3.3. TEM ........ 4
S.3.3.4. STM ........ 4
S.3.3.5. ESI-MS and LDI-MS ........ 5
S.3.3.6. XPS ........ 5
S.3.3.7. SERS ........ 5
S.3.3.8. TGA ........ 6
S.3.3.9. Zeta potential measurements ........ 6

S.3.4. Synthetic procedures ........ 6
S.3.4.1. General procedure for N-alkylation of benzimidazole derivatives ........ 6
S.3.4.2. Synthesis of 5,6-bis(dodecyloxy)-1,3-diisopropylbenzimidazolium bromide ........ 6
S.3.4.3. Synthesis of 5,6-bis(dodecyloxy)-1,3-diethylbenzimidazolium bromide ........ 6
S.3.4.4. 1,3-Diisopropyl-5-(hexyloxy)-1*H*-benzo[*d*]imidazol-3-ium bromide. ........ 7
S.3.4.5. 1,3-Diethyl-5-(hexyloxy)-1*H*-benzo[*d*]imidazol-3-ium bromide. ........ 7
S.3.4.6. General Procedure for the Synthesis of Gold Complexes ........ 7
S.3.4.7. Synthesis of 5,6-bis(dodecyloxy)-1,3-diisopropylbenzoimidazol-2-ylidene gold(I) bromide $^{(RO)2}NHC^{iPr}$-AuBr. 8
S.3.4.8. Synthesis of 5,6-bis(dodecyloxy)-1,3-diethylbenzoimidazol-2-ylidene gold(I) bromide (RO)2NHCEt-AuBr. 8
S.3.4.9. 5-(Hexyloxy)-1,3-diisopropylbenzoimidazol-2-ylidene gold(I) bromide $^{RO}NHC^{iPr}$-AuBr. ........ 8
S.3.4.10. 5-(Hexyloxy)-1,3-diethylbenzoimidazol-2-ylidene gold(I) bromide $^{RO}NHC^{Et}$-AuBr. ........ 9
S.3.4.11. General Procedure for the Synthesis of Gold NPs via the Bottom-Up Approach ........ 9
S.3.4.12. Synthesis of $^{(RO)2}NHC^{iPr}$-AuNP. ........ 9
S.3.4.13. Synthesis of (RO)2NHCEt-AuNP. ........ 9
S.3.4.14. General Procedure for the Synthesis of $^{(RO)2}NHC^{iPr}$-, $^{(RO)2}NHC^{Et}$-, $^{RO}NHC^{iPr}$-, $^{RO}NHC^{Et}$-AuNPs via the Top-Down Approach ........ 9
S.3.5. Crystal data and structure refinement parameters details ........ 10
S.3.6. Mass analysis of bottom-up synthesized $^{(RO)2}NHC^{Et}$-AuNP and $^{(RO)2}NHC^{iPr}$-AuNP. ........ 11
S.3.7. XPS spectra of $^{(RO)2}NHC^{iPr}$-AuBr and $^{(RO)2}NHC^{Et}$-AuBr complexes. ........ 12
S.3.8. XPS spectra of bottom-up synthesized $^{(RO)2}NHC^{iPr}$-AuNP and $^{(RO)2}NHC^{Et}$-AuNP. ........ 14
S.3.9. Thermogravimetric analysis of NHC–gold complexes and bottom-up synthesized gold nanoparticles (AuNPs). 16
S.3.10. SER spectra for the upright and flat $NHC^{iPr}$ configurations on $Au_2$ and $Au_{58}$ clusters ........ 20
S.3.11. TEM images of (a) citrate-capped gold NPs, (b) top-down synthesized $^{(RO)2}NHC^{iPr}$-AuNP, and (c) top-down synthesized $^{(RO)2}NHC^{Et}$-AuNP. ........ 21
S.3.12. XPS spectra of top-down synthesized $^{(RO)2}NHC^{iPr}$-AuNP and $^{(RO)2}NHC^{Et}$-AuNP. ........ 22
S.3.13. Orientation of $^{(MeO)2}NHC^{iPr}$ on gold flat surfaces ........ 22
S.3.14. XPS spectra of top-down synthesized $^{RO}NHC^{iPr}$-AuNP and $^{RO}NHC^{Et}$-AuNP. ........ 24
S.3.15. STM Data ........ 25
S.3.16. TEM images of bottom-up synthesized $^{(RO)2}NHC^{iPr}$-AuNP and $^{(RO)2}NHC^{Et}$-AuNP. ........ 27
S.3.17. Thermal stability data of top-down synthesized AuNPs. ........ 31
S.3.18. Comparative study on the thermal stability of bottom-up synthesized $^{(RO)2}NHC^{iPr}$-AuNP. ........ 34
S.3.19. Zeta potential measurement data for top-down synthesized $^{(RO)2}NHC^{iPr}$-, $^{(RO)2}NHC^{Et}$-, $^{RO}NHC^{iPr}$-, and $^{RO}NHC^{Et}$-AuNPs 35

S.3.20. NMR Spectra ........ 39
S.3.21. Bond Lengths and Bond Angles of NHC gold complexes ........ 55
S4. References ........ 60

# S2. Computational details

For all Density functional theory (DFT) calculations, the Becke-Perdew (BP86) exchange-correlation functional[1,2] with dispersion correction Grimme3 BJDAMP was used.[3] The triple-ζ Slater type (TZP) basis set with small frozen cores from the ADF basis set library was used. To account for relativistic effects, zeroth-order regular approximation (ZORA) was used.[4] Full geometry optimizations were performed. The vibrational frequencies and normal modes were calculated within the harmonic approximation. AOResponse module from ADF engine was used for polarizability calculations at the static limit with the Adiabatic Local Density Approximation (ALDA).[5] The Raman intensity was calculated based on squared polarizability component derivatives with respect to the normal mode displacements. The structure of the systems were plotted with PyMOL.[6]

# S3. Experimental Section

## S.3.1. Reagents

Chloro(dimethylsulfide)gold(I) ($Me_2SAuCl$) [7] and 5,6-bis(dodecyloxy)benzimidazole[8] were prepared following the published procedures. 5-(Hexyloxy)-1*H*-benzo[*d*]imidazole was synthesized using a procedure similar to that employed for the synthesis of 5-(dodecyloxy)-1*H*-benzo[d]imidazole.[9] Iron powder, 325 Mesh (98%) was purchased from Oakwood Chemical. 1-Bromododecane, ammonium chloride ($NH_4Cl$), nitric acid ($HNO_3$, ACS reagent, 70%), and sulfuric acid ($H_2SO_4$) were purchased from Fisher Scientific. Catechol, 2-bromopropane, potassium carbonate ($K_2CO_3$), sodium borohydride ($NaBH_4$) were purchased from Sigma–Aldrich. Ethanol (99.5%) was purchased from Commercial Alcohols.

## S.3.2. Piranha solution safety

Piranha solution is a mixture of concentrated sulfuric acid ($H_2SO_4$) and hydrogen peroxide ($H_2O_2$). It is a highly reactive cleaning agent used to remove organic residues. Due to its extreme corrosiveness and potential for violent reactions, strict safety measures must be followed. Always add hydrogen peroxide to sulfuric acid slowly to minimize exothermic reactions. Wear appropriate PPE, including acid-resistant gloves, a lab coat, and safety goggles. Use only glass containers and avoid organic materials, as spontaneous ignition can occur. Never store Piranha solution in sealed containers due to gas evolution risks. Dispose of it properly by dilution and neutralization with sodium bicarbonate, following institutional hazardous waste guidelines. In case of exposure, flush with copious amounts of water and seek medical attention immediately. Adhering to these precautions ensures a safer laboratory environment.

## S.3.3. Characterization Methods

### S.3.3.1. NMR

$^1$H and $^{13}$C nuclear magnetic resonance (NMR) spectra were recorded on Bruker AV500 or AV600 spectrometers at 25 °C. $^1$H chemical shifts were referenced to the residual protons of the deuterated solvent (δ = 7.26 ppm for $CDCl_3$; δ = 3.34 ppm for $CD_3OD$; $^{13}$C chemical shifts were referenced to the $CDCl_3$ signal at δ = 77.00 ppm and $CD_3OD$ signal at δ = 50.00 ppm. The following abbreviations are used to describe NMR signals: s (singlet), d (doublet), t (triplet), quint (quintet), ap (apparent), br (broad). Some of the signals of Cp protons show an unresolved fine structure; these signals are indicated as multiplets. Coupling constants obtained from $^1$H NMR spectra are associated with an error, but still reported to the first decimal point (the digital resolution in $^1$H NMR spectra is 0.2-0.3 Hz). Assignments for newly synthesized compounds were supported by additional NMR experiments (HSQC, HMBC, COSY). NMR data was processed using Bruker TopSpin Software.

### S.3.3.2. UV−vis

UV–vis absorbance spectra were recorded using a Cary 3500 Multizone UV–vis spectrophotometer using 1 $cm^3$ quartz cuvettes. UV–vis absorbance spectra were displayed using Origin 2023 software

### S.3.3.3. TEM

Transmission electron microscopy (TEM) images were acquired using a Talos F200i scanning transmission electron microscope operated at an acceleration voltage of 40−200 kV and equipped with a 16-megapixel camera. Small amount of gold nanoparticles (NPs) was dissolved in dichloromethane (HPLC grade) and filtered using a PTFE syringe filter with a pore size of 0.22 μm. Dichloromethane solution was drop-casted onto a copper TEM grid and dried on air overnight.

### S.3.3.4. STM

STM images were acquired on single crystal gold (111) surfaces cleaned and ordered in ultrahigh vacuum (UHV) using standard procedures: argon-ion sputtering and annealing. NHCs were deposited onto room-temperature surfaces by heating a bench-stable benzimidazolium hydrogen carbonate in vacuo, which generated free NHCs. Constant-current topographical STM images were collected with the surfaces cooled to 77 K using a CreaTec LT-STM with a mechanically cut Pt/Ir tip. Relatively small bias voltages, typically in the range 20-200\,mV, were used to minimize the dependence of topographic images on the overlayer density of states (DOS)

### S.3.3.5. ESI-MS and LDI-MS

Electrospray ionization mass spectrometry (ESI-MS) analysis was recorded on Agilent 6545XT AdvanceBio Q-TOF mass spectrometer equipped with a Dual Agilent JetStream source. Acetonitrile-dichloromethane (1:1 v/v) solutions of samples (ca. 0.1 mg/mL) were directly infused at a flow rate of 60 μL/h. The source, operating in positive mode, and typical parameters used for the measurements were as follows: nebulizer (20 psi), drying gas temperature (300°C), drying gas flow rate (8 L/min), sheath gas temperature (300°C), sheath gas flow (11 L/min), capillary voltage (3,500 V), fragmentor voltage (150 V) and skimmer voltage (65 V). The instrument was first calibrated with ESI-L low concentration tuning mix (Agilent Technologies) under the same parameters as an

external reference. Data were acquired and analyzed using Agilent MassHunter (11.0) and Qualitative Analysis (10.0) software respectively.

One Bruker UltrafleXtreme MALDI-ToF mass spectrometer was used for all Laser desorption ionization mass spectrometry (LDI-MS) measurements of NHC functionalized materials. The mass spectrometer was operated in positive ion, reflectron mode and is equipped with a 355 nm frequency tripped ND:YAG laser. Calibration of the mass spectrometer was performed according to the method of Havel[10] using red phosphorous. Bottom-up gold nanoparticle samples were analyzed either on a gold mirror or on a stainless-steel metal target plate (Bruker). Experimental LDI-MS data was processed in MATLAB using the *msbackadj* function and smoothed using a Savitsky-Golay filter.

### S.3.3.6. XPS

X-ray photoelectron spectroscopy (XPS) measurements were performed using Kratos Axis Nova ultrahigh vacuum (UHV) surface analysis instrument using AlKα X-rays (1486.6 eV). For NPs, dichloromethane solutions of the samples were drop-casted on the glass chips and dried at ambient conditions. For gold complexes, supported samples were mounted on an aluminum sample holder with double sided copper tape. The sample holder was stored in the sample entry chamber under high vacuum (~10–8 Torr) overnight before transferring to the acquisition chamber (ultra-high vacuum, ~10–9 Torr). Survey spectra were collected at pass energy of 160 eV, along with high-resolution element scan on all major element lines at 20 eV pass energy. Acquired data was processed and displayed using CasaXPS software. In all cases, the C 1s peak corresponding to aliphatic carbon was used for charge correction of spectra (284.8 eV).

### S.3.3.7. SERS

For surface-enhanced Raman spectroscopy (SERS) analysis, the top-down NHC-AuNPs were treated with 200 microliters of aqueous 1 M NaBr for every 1 mL of gold colloids. For all surface-enhanced Raman spectroscopy (SERS) measurements, a custom-made Raman microscope was used.[11–13] A 633 nm HeNe laser (ThorLabs, Newton, NJ) was directed into an inverted microscope (Nikon, Melville, NY). The laser was focused onto the sample using a 20x objective (Nikon), which was also used to collect scattered light. Scattered light was then filtered through a Rayleigh rejection filter (Semrock, Lake Forest, IL), directed into a spectrometer (Princeton Instruments Acton), and spectra were recorded using a Princeton Instruments CCD detector with liquid nitrogen cooling (Princeton Instruments, Trenton, NJ).

All SERS spectra were background subtracted in MATLAB using *msbackadj* and averaged for at least three measurements for each sample. For SERS measurements of bottom-up NHC-AuNPs, the nanoparticles were resuspended in dichloromethane and drop-cast in 3 or 5 microliter aliquots onto the gold mirror. Note that bottom-up **$^{(RO)2}$NHC$^{Et}$-AuNP** was first annealed at 70 °C for 24 hours prior to SERS measurement. For SERS measurements of NHC functionalized gold mirrors, 20 microliters of Lee and Meisel gold colloids were drop-cast onto the gold mirror and allowed to dry in open air. SERS "hot spots" form in the regions between the AuNPs and the gold mirror, facilitating SERS analysis.[14–16]

#### S.3.3.8. TGA

Thermogravimetric analysis (TGA) experiments were performed using TGA Q500 with platinum crucibles. A constant heating rate of 10 °C/min and gas purging ($N_2$) at a flow rate of 60 mL/min were used.

#### S.3.3.9. Zeta potential measurements

Zeta potential measurements were carried out using a Zetasizer Nano ZS (Malvern Instruments Ltd., UK). Nanoparticle samples were dispersed in water (pH = 4), and measurements were conducted at room temperature.

### S.3.4. Synthetic procedures

#### S.3.4.1. General procedure for N-alkylation of benzimidazole derivatives

In a 75 mL pressure tube, substituted benzimidazoles, $K_2CO_3$, acetonitrile (15 mL), and the respective alkyl bromide were added. The flask was sealed and stirred at 90 °C for 72 h. Then excess alkyl bromide, solvent and volatiles were evaporated in vacuo. The residual solid was triturated and sonicated in diethyl ether (2 × 4 mL), which was then decanted off. Subsequent drying under vacuum afforded the desired product as an off-white powder.

#### S.3.4.2. Synthesis of 5,6-bis(dodecyloxy)-1,3-diisopropylbenzimidazolium bromide.

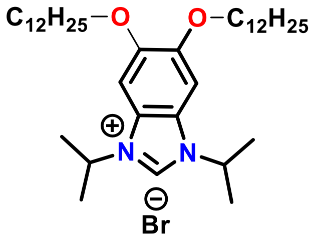


Prepared from the general procedure above using 5,6-bis(dodecyloxy)benzimidazole (1.60 g, 3.28 mmol, 1 equiv.), $K_2CO_3$ (0.545 g, 3.94 mmol, 1.2 equiv.), and 2-bromopropane (3.5 mL, 35 mmol, 10.7 equiv.). Reaction afforded a white-colored solid (1.85 g, 86% yield). $^1$H NMR ($CDCl_3$, 500 MHz): δ 11.12 (s, 1H, NCHN), 7.01 (s, 2H, Ar-H), 5.03 (sep, $^3J_{HH}$ = 6.6 Hz, 1H, $CHMe_2$), 4.05 (t, $^3J_{HH}$ = 6.7 Hz, 4H, $OCH_2$), 1.89 (quint, $^3J_{HH}$ = 6.8 Hz, 4H, $CH_2$), 1.82 [d, $^3J_{HH}$ = 6.6 Hz, 12H, $CH(CH_3)_2$], 1.51 (quint, $^3J_{HH}$ = 6.8 Hz, 4H, $CH_2$), 1.43-1.18 (m, 32H, $CH_2$), 0.88 (t, $^3J_{HH}$ = 6.6 Hz, 6H, $CH_3$) ppm. $^{13}C\{^1H\}$ NMR ($CDCl_3$, 126 MHz): δ 150.2, 138.2, 124.7, 96.5, 70.1, 51.9, 31.9, 29.7, 29.7, 29.6 (two carbon peaks overlapped), 29.4 (two carbon peaks overlapped), 29.0, 26.0, 22.7, 22.3, 14.1 ppm. ESI-MS $[M]^+$: calcd 571.5203 for $C_{37}H_{67}N_2O_2^+$; found 571.7206

#### S.3.4.3. Synthesis of 5,6-bis(dodecyloxy)-1,3-diethylbenzimidazolium bromide.

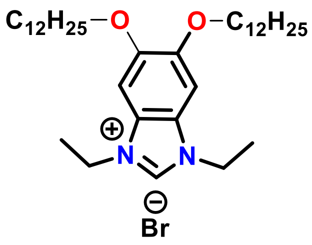


Prepared from the general procedure above using 5,6-bis(dodecyloxy)benzimidazole (1.0 g, 2.0 mmol, 1 equiv.), $K_2CO_3$ (0.340 g, 2.46 mmol, 1.2 equiv.), and bromoethane (1.5 mL, 20 mmol, 10.0 equiv.). Reaction afforded a white-colored solid (1.08 g, 87% yield). $^1$H NMR ($CDCl_3$, 500 MHz): δ 11.07 (s, 1H, NCHN), 6.99 (s, 2H, Ar-H), 4.58 (q, $^3J_{HH}$ = 7.3 Hz, 4H, $CH_2CH_3$), 4.05 (t, $^3J_{HH}$ = 6.7 Hz, 4H, $OCH_2$), 1.88 (quin , $^3J_{HH}$ = 6.8 Hz, 4H, $CH_2$), 1.69 (t, $^3J_{HH}$ = 7.3 Hz, 6H, $CH_2CH_3$), 1.50 (quint, $^3J_{HH}$ = 6.8 Hz, 4H, $CH_2$), 1.37 (quint, $^3J_{HH}$ = 6.8 Hz, 4H, $CH_2$),1.34-1.20 (m, 28H, $CH_2$), 0.88 (t, $^3J_{HH}$ = 6.7 Hz, 6H, $CH_3$) ppm.$^{13}C\{^1H\}$ NMR ($CDCl_3$, 126 MHz): δ 150.5, 139.4, 125.0, 95.6, 70.1, 42.6, 31.9, 29.7, 29.6, 29.6

(two carbon peaks overlapped), 29.4 (two carbon peaks overlapped), 29.0, 26.0, 22.7, 14.9, 14.1 ppm. ESI-MS $[M]^+$: calcd 543.4884 for $C_{35}H_{63}N_2O_2^+$; found 543.4302

### S.3.4.4. 1,3-Diisopropyl-5-(hexyloxy)-1*H*-benzo[*d*]imidazol-3-ium bromide.

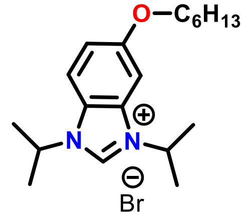


Prepared from the general procedure above using 5-(hexyloxy)-1*H*-benzo[*d*]imidazole (2.0 g, 9.2 mmol), $K_2CO_3$ (1.90 g, 13.7 mmol), and 2-bromopropane (8.5 mL, 90 mmol). Reaction afforded a white-colored solid (3.10 g, 88% yield). $^1$H NMR ($CDCl_3$, 500 MHz): δ 11.17 (s, 1H, NCHN), 7.63 (d, $^3J_{HH}$ = 8.9 Hz, 1H, Ar-H), 7.19 (d, $^3J_{HH}$ = 8.6 Hz, 1H, Ar-H), 7.08 (br, 1H, Ar-H), 5.14 (sep, $^3J_{HH}$ = 6.45 Hz, 1H, CHMe$_2$), 5.05 (sep, $^3J_{HH}$ = 6.45 Hz, 1H, CHMe$_2$), 4.03 (t, 2H, OCH$_2$), 1.88-1.77 (m, 12H of CH(CH$_3$)$_2$ overlapped with 2H of CH$_2$), 1.47 (m, 2H, CH$_2$), 1.34 (m, 4H, CH$_2$), 0.89 (m, 3H, CH$_3$) ppm. $^{13}$C{$^1$H} NMR ($CDCl_3$, 126 MHz): δ 158.6, 139.7, 132.0, 124.7, 117.0, 114.4, 96.6, 69.2, 52.2, 51.8, 31.4, 28.9, 25.6, 22.5, 22.2, 22.1, 13.9 ppm. ESI-MS $[M]^+$: calcd 303.2436 for $C_{19}H_{31}N_2O^+$; found 303.3507.

### S.3.4.5. 1,3-Diethyl-5-(hexyloxy)-1*H*-benzo[*d*]imidazol-3-ium bromide.

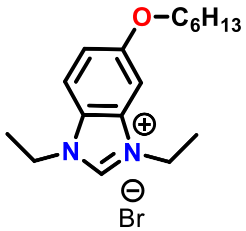


Prepared from the general procedure above using 5-(hexyloxy)-1*H*-benzo[*d*]imidazole (2.0 g, 9.2 mmol), $K_2CO_3$ (1.50 g, 11.0 mmol), and bromoethane (6.8 mL, 92 mmol). Reaction afforded a white-colored solid (3.0 g, 97% yield). $^1$H NMR ($CDCl_3$, 500 MHz): δ 11.3 (s, 1H, NCHN), 7.58 (d, $^3J_{HH}$ = 9.0 Hz, 1H, Ar-H), 7.20 (d, $^3J_{HH}$ = 9.0 Hz, 1H, Ar-H), 7.04 (s, 1H, Ar-H), 4.61 (q, $^3J_{HH}$ = 7.2 Hz, 4H, Ar-H, CH$_2$CH$_3$), 4.03 (t, $^3J_{HH}$ = 6.4 Hz, 2H, OCH$_2$), 1.81 (qint , $^3J_{HH}$ = 6.8 Hz, 2H, CH$_2$), 1.69 [t, $^3J_{HH}$ = 7.2 Hz, 6H, CH$_2$CH$_3$], 1.47 (m, 2H, CH$_2$), 1.33 (m, 4H, CH$_2$), 0.89 (t, $^3J_{HH}$ = 6.5 Hz, 3H, CH$_3$) ppm. $^{13}$C{$^1$H} NMR ($CDCl_3$, 126 MHz): δ 159.0, 141.2, 132.3, 125.12, 117.5, 113.6, 95.7, 69.2, 42.8, 42.6, 31.4, 28.9, 25.6, 22.5, 14.9, 14.8, 13.9 ppm. ESI-MS $[M]^+$: calcd 275.2123 for $C_{17}H_{27}N_2O^+$; found 275.3043.

### S.3.4.6. General Procedure for the Synthesis of Gold Complexes

A glass pressure tube was charged with the appropriate benzimidazolium salt, ($Me_2S$)AuCl, $K_2CO_3$, and acetone (10 mL). For **$^{(RO)2}$NHC$^{iPr}$-AuBr** and **$^{(RO)2}$NHC$^{Et}$-AuBr**, the mixture was heated in a 60 °C oil bath for 6 h. For **$^{RO}$NHC$^{iPr}$-AuBr** and **$^{RO}$NHC$^{Et}$-AuBr,** the mixture was heated in a 70 °C oil bath for 3 h. Then the reaction cooled to room temperature. After cooling, the solvent was removed in vacuo to afford the crude residue. The crude residue was redissolved in DCM and filtered through Celite to remove any inorganic salts. The filtrate was reduced and then precipitated in petroleum ether to afford the pure complex as a white powder.

### S.3.4.7. Synthesis of 5,6-bis(dodecyloxy)-1,3-diisopropylbenzoimidazol-2-ylidene gold(I) bromide $^{(RO)2}$NHC$^{iPr}$-AuBr.

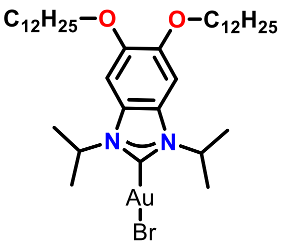


Reagent 5,6-bis(dodecyloxy)-1,3-diisopropylbenzimidazolium bromide (100 mg, 0.153 mmol), $Me_2SAuCl$ (45 mg, 0.152 mmol), and $K_2CO_3$ (63 mg, 0.46 mmol) were reacted to afford **$^{(RO)2}$NHC$^{iPr}$-AuBr** (114 mg) as an off-white solid in 88 % yield after purification. $^1H$ NMR ($CDCl_3$, 500 MHz): δ 7.03 (s, 2H, Ar-H), 5.40 (sep, $^3J_{HH}$ = 6.6 Hz, 2H, C<u>H</u>Me$_2$), 4.02 (t, $^3J_{HH}$ = 6.7 Hz, 4H, $OCH_2$), 1.85 (qunt , $^3J_{HH}$ = 6.8 Hz, 4H, $CH_2$), 1.70 [d, $^3J_{HH}$ = 6.6 Hz, 12H, CH(C<u>H</u>$_3$)$_2$], 1.51 (quint, $^3J_{HH}$ = 6.8 Hz, 4H, $CH_2$), 1.42-1.18 (m, 32H, $CH_2$), 0.88 (t, $^3J_{HH}$ = 6.6 Hz, 6H, $CH_3$) ppm. $^{13}C\{^1H\}$ NMR ($CDCl_3$, 126 MHz): δ 177.1, 147.8, 126.3, 98.3, 70.4, 53.8, 31.9, 29.7, 29.7, 29.6 (two carbon peaks overlapped), 29.4, 29.4, 29.3, 26.1, 22.7, 21.8, 14.1 ppm. ESI-MS [M]$^+$: calcd 1337.9914 for $C_{74}H_{132}AuN_4O_4^+$; found 1337.9618. Note: While $^{13}C$ NMR and crystallographic data confirm the formation of the **$^{(RO)2}$NHC$^{iPr}$-AuBr** complex, mass spectrometry conditions induce its conversion to the $[(NHC)_2Au]^+$complex.

### S.3.4.8. Synthesis of 5,6-bis(dodecyloxy)-1,3-diethylbenzoimidazol-2-ylidene gold(I) bromide (RO)2NHCEt-AuBr.

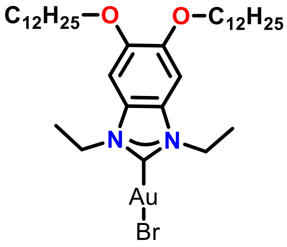


Reagent 5,6-bis(dodecyloxy)-1,3-diethylbenzimidazolium bromide (100 mg, 0.160 mmol), $Me_2SAuCl$ (52 mg, 0.176 mmol), and $K_2CO_3$ (66 mg, 0.48 mmol) were reacted to afford gold complex $^{(RO)2}$NHC$^{Et}$-AuBr (95 mg) as an off-white solid in 72 % yield after purification. $^1H$ NMR ($CDCl_3$, 500 MHz): δ 6.89 (s, 2H, Ar-H), 5.45 (q, $^3J_{HH}$ = 6.7 Hz, 4H, C<u>H</u>$_2$CH$_3$), 4.03 (t, $^3J_{HH}$ = 6.7 Hz, 4H, $OCH_2$), 1.86 (quint, $^3J_{HH}$ = 6.8 Hz, 4H, $CH_2$), 1.52 [m, 6H, CH$_2$C<u>H</u>$_3$, overlapped with the next peak], 1.51 (quint, $^3J_{HH}$ = 6.8 Hz, 4H, $CH_2$), 1.42-1.18 (m, 32H, $CH_2$), 0.88 (t, $^3J_{HH}$ = 6.6 Hz, 6H, $CH_3$) ppm. $^{13}C\{^1H\}$ NMR ($CDCl_3$, 126 MHz): δ 178.3, 148.6, 126.6, 99.6, 70.4, 43.7, 31.9, 29.7, 29.6, 29.6 (two carbon peaks overlapped), 29.4, 29.3, 29.2, 26.0, 22.7, 15.5, 14.0 ppm. ESI-MS [M]$^+$: calcd 1281.9288 for $C_{70}H_{124}AuN_4O_4^+$; found 1282.0456. Note: While $^{13}C$ NMR and crystallographic data confirm the formation of the **$^{(RO)2}$NHC$^{Et}$-AuBr** complex, mass spectrometry conditions induce its conversion to the $[(NHC)_2Au]^+$complex.

### S.3.4.9. 5-(Hexyloxy)-1,3-diisopropylbenzoimidazol-2-ylidene gold(I) bromide $^{RO}$NHC$^{iPr}$-AuBr.

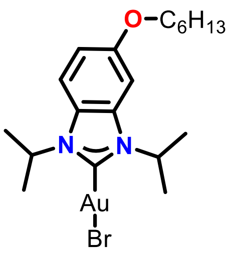


Reagent 1,3-diisopropyl-5-(hexyloxy)-1*H*-benzo[*d*]imidazol-3-ium bromide (100 mg, 0.260 mmol), $Me_2SAuCl$ (85 mg, 0.29 mmol), and $K_2CO_3$ (108 mg, 0.781 mmol) were reacted to afford gold complex **$^{RO}$NHC$^{iPr}$-AuBr** (120 mg) as an off-white solid in 80 % yield after purification. $^1H$ NMR ($CDCl_3$, 500 MHz): δ 7.50 (d, $^3J_{HH}$ = 8.9 Hz, 1H, Ar-H), 7.05 (br, 1H, Ar-H), 6.96 (d, $^3J_{HH}$ = 8.6 Hz, 1H, Ar-H), 5.40 (m, 2H, C<u>H</u>Me$_2$), 4.00 (t, $^3J_{HH}$ = 6.2 Hz, 2H, $OCH_2$), 1.82 (m, 2H, $CH_2$), 1.70 [d, $^3J_{HH}$ = 6.7 Hz, 12H, CH(C<u>H</u>$_3$)$_2$], 1.49 (m, 2H, $CH_2$), 1.35 (m, 4H, $CH_2$), 0.91 (t, 3H, $CH_3$) ppm. $^{13}C\{^1H\}$ NMR ($CDCl_3$, 126 MHz): δ 179.8, 157.4, 134.3, 127.5, 114.4, 113.8, 98.8, 70.0, 55.2, 54.7, 32.5, 30.1, 26.7, 23.6, 22.7, 22.6, 15.0 ppm. ESI-MS [M]$^+$: calcd 801.4382 for $C_{38}H_{60}AuN_4O_2^+$; found 801.5024. Note: While $^{13}C$ NMR confirms the formation of the **$^{RO}$NHC$^{iPr}$-AuBr** complex, mass spectrometry conditions induce its conversion to the $[(NHC)_2Au]^+$complex.

### S.3.4.10. 5-(Hexyloxy)-1,3-diethylbenzoimidazol-2-ylidene gold(I) bromide $^{RO}$NHC$^{Et}$-AuBr.

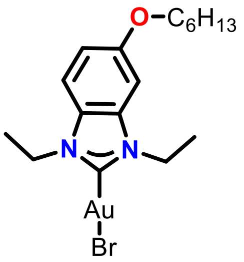


Reagent 1,3-diethyl-5-(hexyloxy)-1*H*-benzo[*d*]imidazol-3-ium bromide (100 mg, 0.281 mmol), $Me_2SAuCl$ (91.0 mg, 0.309 mmol), and $K_2CO_3$ (116 mg, 0.839 mmol) were reacted to afford gold complex **$^{RO}$NHC$^{Et}$-AuBr** (115 mg) as an off-white solid in 75 % yield after purification. $^1$H NMR ($CDCl_3$, 500 MHz): δ 7.33 (d, $^3J_{HH}$ = 9.0 Hz, 1H, Ar-H), 7.02 (dd, $^3J_{HH}$ = 9.0 Hz, $^4J_{HH}$ = 1.8 Hz, 1H, Ar-H), 6.96 (d, , $^4J_{HH}$ = 1.8, 1H, Ar-H), 4.47 (m, 4H, $C\underline{H}_2CH_3$), 4.01 (t, $^3J_{HH}$ = 6.5 Hz, 2H, $OCH_2$), 1.82 (m, 2H, $CH_2$), 1.52 [t, $^3J_{HH}$ = 7.3 Hz, 6H, $CH_2C\underline{H}_3$, overlapped with the next peak], 1.51 (m, 2H, $CH_2$), 1.35 (m, 4H, $CH_2$), 0.91 (t, $^3J_{HH}$ = 6.5 Hz, 3H, $CH_3$) ppm. $^{13}C\{^1H\}$ NMR ($CDCl_3$, 126 MHz): δ 180.9, 158.3, 134.6, 128.0, 114.7, 112.9, 96.7, 70.1, 44.8, 44.6, 32.5, 30.1, 26.7, 23.5, 16.5, 16.3, 15.0 ppm. ESI-MS [M]$^+$: calcd 745.3756 for $C_{34}H_{52}AuN_4O_2^+$; found 745.4306. Note: While $^{13}$C NMR confirms the formation of the **$^{RO}$NHC$^{Et}$-AuBr** complex, mass spectrometry conditions induce its conversion to the $[(NHC)_2Au]^+$complex.

### S.3.4.11. General Procedure for the Synthesis of Gold NPs via the Bottom-Up Approach

A 20 mL vial containing a stir bar and the gold complex was charged with 3 mL of DCM. To the stirring solution, a thoroughly sonicated solution of $NaBH_4$ in 99% ethanol (1 mL) was added. The reaction was allowed to stir at room temperature for 20 hours, where color change from pale yellow to dark red was observed. After this time, 20 mL of water was added to the mixture, and the nanoparticles (NPs) were extracted with DCM (2 × 10 mL). The combined extracts were concentrated using a rotary evaporator, maintaining the temperature below 25 °C. The resulting red solid was dissolved in 5.0 mL of diethyl ether and filtered through a PTFE syringe filter with a pore size of 0.22 µm. The solvent was then removed under vacuum, and the resulting NPs were washed with ethanol (3 × 3 mL) to yield pure dark red NPs.

### S.3.4.12. Synthesis of $^{(RO)2}$NHC$^{iPr}$-AuNP.

Reagent 5,6-bis(dodecyloxy)-1,3-diisopropylbenzoimidazol-2-ylidene gold(I) bromide (20 mg, 0.023 mmol) and $NaBH_4$ (4.4 mg, 0.11 mmol) were reacted to afford 1-AuNP as dark red solid.

### S.3.4.13. Synthesis of (RO)2NHCEt-AuNP.

Reagent 5,6-bis(dodecyloxy)-1,3-diethylbenzoimidazol-2-ylidene gold(I) bromide (20 mg, 0.024 mmol) and $NaBH_4$ (4.6 mg, 0.12 mmol) were reacted to afford 2-AuNP as dark red solid.

### S.3.4.14. General Procedure for the Synthesis of $^{(RO)2}$NHC$^{iPr}$-, $^{(RO)2}$NHC$^{Et}$-, $^{RO}$NHC$^{iPr}$-, $^{RO}$NHC$^{Et}$-AuNPs via the Top-Down Approach

Top-down NHC-functionalized AuNPs were prepared according to the Camden and Jenkins method.[17] First Lee and Meisel[18] gold colloids were synthesized and the resulting particles characterized using TEM (Fig. S11a). The resulting particle size of 21±4 nm diameter is in excellent agreement with previous reports.[19,20] These citrate-capped AuNPs were then treated with 1 microliter of 10 mM NHC-Au complex in dichloromethane for every 1 mL of gold

nanoparticles. The NHC-treated gold colloids were also characterized with TEM (Fig. S11b-c), and no changes in size or morphology were observed after the ligand exchange.

## S.3.5. Crystal data and structure refinement parameters details

**Table 1**: Crystal data and structure refinement parameters details of $^{(RO)2}$**NHC$^{iPr}$-AuNP** and $^{(RO)2}$**NHC$^{Et}$-AuNP**.

| Identification code | $^{(RO)2}$**NHC$^{iPr}$-AuNP** | $^{(RO)2}$**NHC$^{Et}$-AuNP** |
|---|---|---|
| CCDC no | 2410266 | 2410267 |
| Empirical formula | $C_{37}H_{66}AuBrN_2O_2$ | $C_{35}H_{62}AuBrN_2O_2$ |
| Formula weight | 847.79 | 819.74 |
| Temperature/K | 298.00 | 298.00 |
| Crystal system | monoclinic | monoclinic |
| Space group | $P2_1/c$ | $P2_1/n$ |
| a/Å | 10.6121(5) | 10.6050(3) |
| b/Å | 11.5625(7) | 32.8095(10) |
| c/Å | 32.624(2) | 11.2301(3) |
| α/° | 90 | 90 |
| β/° | 97.069(3) | 91.4620(10) |
| γ/° | 90 | 90 |
| Volume/Å$^3$ | 3972.6(4) | 3906.18(19) |
| Z | 4 | 4 |
| $\rho_{calc}$g/cm$^3$ | 1.417 | 1.394 |
| μ/mm$^{-1}$ | 4.740 | 4.818 |
| F(000) | 1728.0 | 1664.0 |
| Crystal size/mm$^3$ | 0.13 × 0.12 × 0.07 | 0.15 × 0.1 × 0.08 |
| Radiation | MoKα (λ = 0.71073) | MoKα (λ = 0.71073) |
| 2Θ range for data collection/° | 3.74 to 37.916 | 3.834 to 52.72 |
| Index ranges | $-9 \le h \le 9, -10 \le k \le 10, -29 \le l \le 29$ | $-13 \le h \le 11, -40 \le k \le 40, -14 \le l \le 13$ |
| Reflections collected | 53212 | 84631 |
| Independent reflections | 3174 [$R_{int}$ = 0.1826, $R_{sigma}$ = 0.0603] | 7934 [$R_{int}$ = 0.0772, $R_{sigma}$ = 0.0352] |
| Data/restraints/parameters | 3174/35/172 | 7934/15/248 |
| Goodness-of-fit on F$^2$ | 1.092 | 1.020 |
| Final R indexes [I>=2σ (I)] | $R_1$ = 0.0802, $wR_2$ = 0.2019 | $R_1$ = 0.0607, $wR_2$ = 0.1562 |
| Final R indexes [all data] | $R_1$ = 0.1166, $wR_2$ = 0.2329 | $R_1$ = 0.1024, $wR_2$ = 0.1836 |
| Largest diff. peak/hole / e Å$^{-3}$ | 1.88/-1.17 | 1.19/-0.77 |

## S.3.6. Mass analysis of bottom-up synthesized $^{(RO)2}NHC^{Et}$-AuNP and $^{(RO)2}NHC^{iPr}$-AuNP.

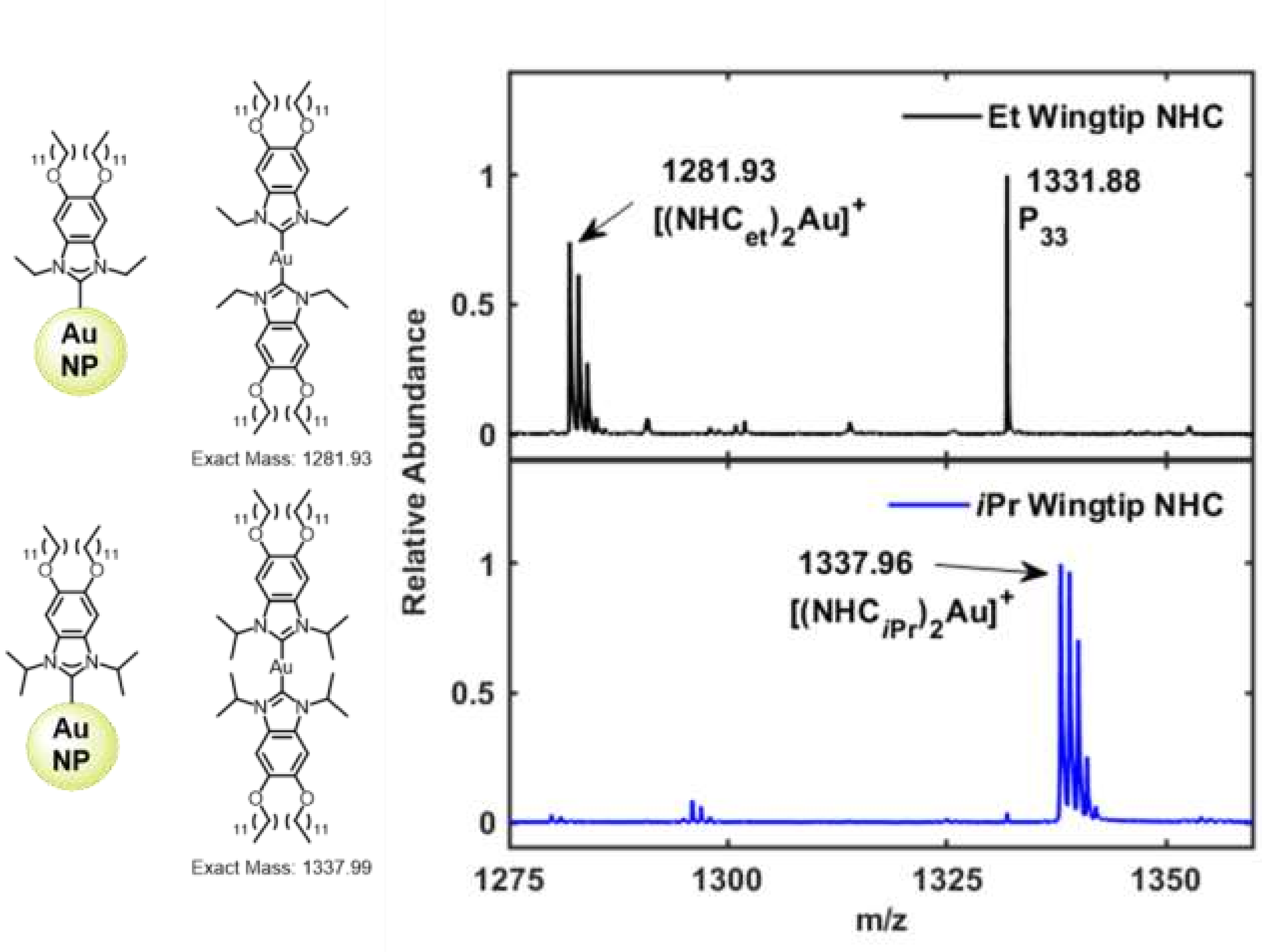


**Fig. S1.** LDI-MS of **$^{(RO)2}NHC^{Et}$-AuNP** (top) and **$^{(RO)2}NHC^{iPr}$-AuNP** (bottom) prepared according to a bottom-up protocol. Note that an internal standard of red phosphorous was used for these measurements, which gives rise to the $P_{33}$ cluster at 1331.88 *m/z*.

## S.3.7. XPS spectra of $^{(RO)2}$NHC$^{iPr}$-AuBr and $^{(RO)2}$NHC$^{Et}$-AuBr complexes.

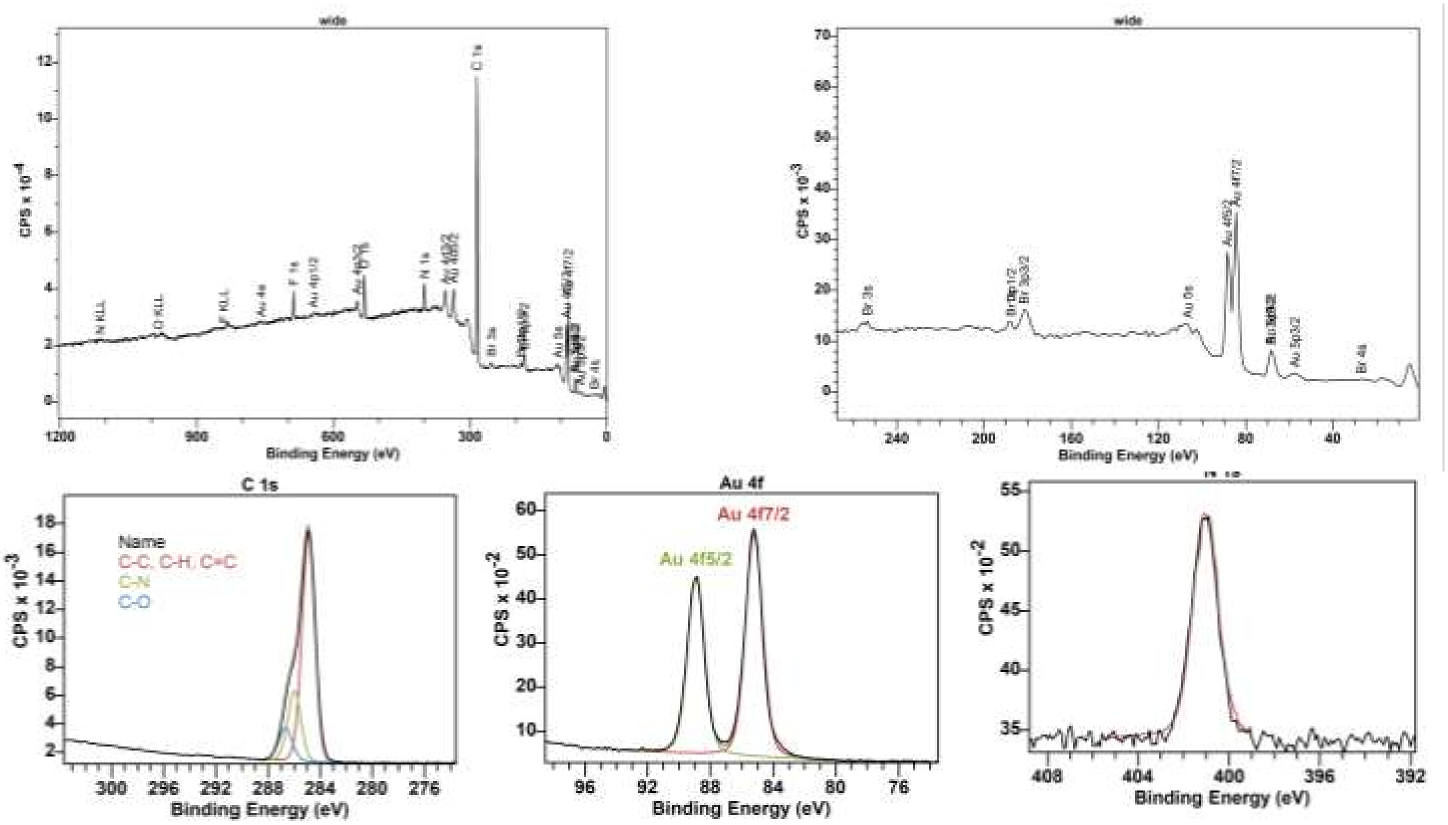


**Fig. S2.** XPS spectra of **$^{(RO)2}$NHC$^{iPr}$-AuBr**.

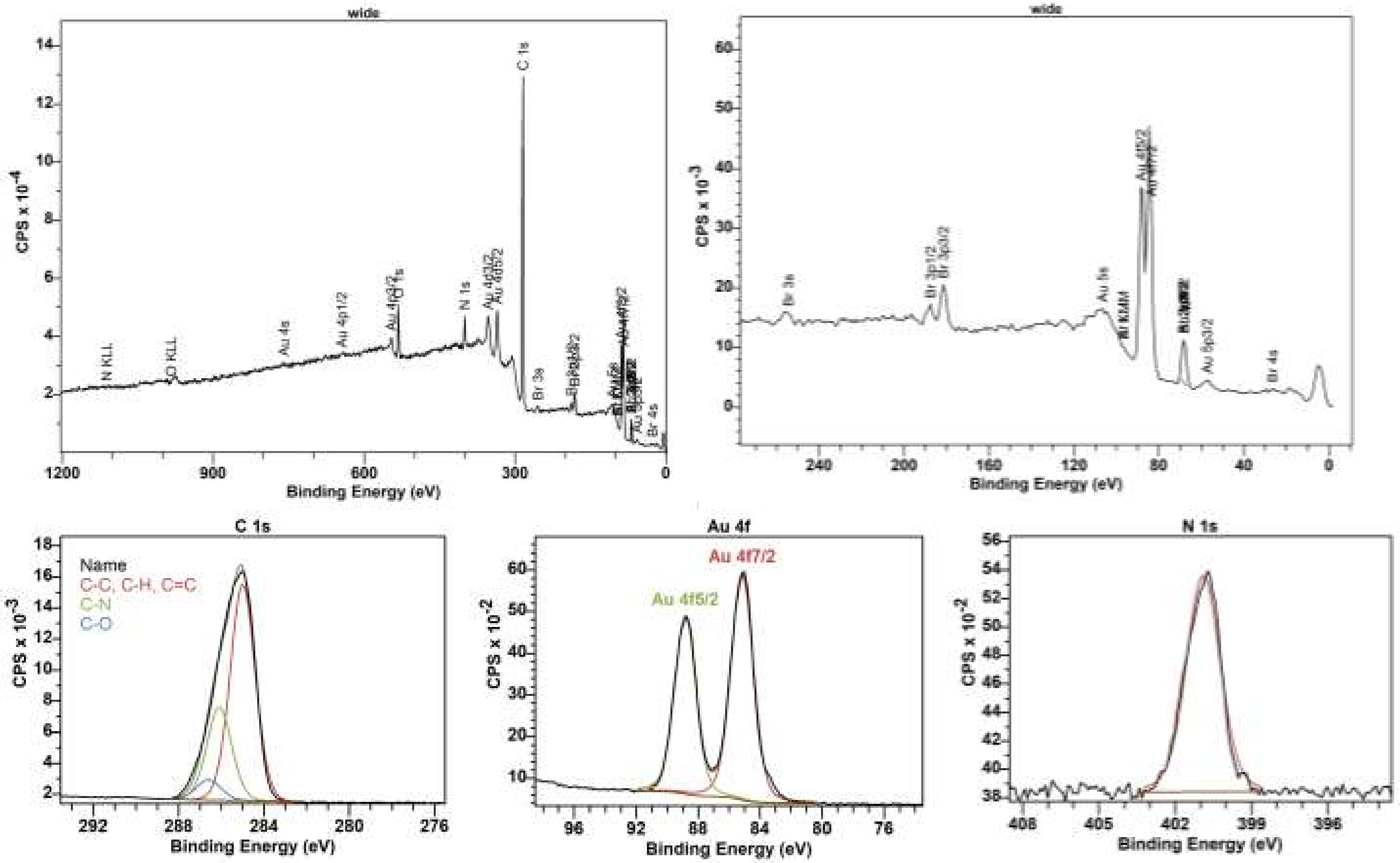


**Fig. S3.** XPS spectra of $^{(RO)2}$**NHC$^{Et}$-AuBr**.

## S.3.8. XPS spectra of bottom-up synthesized $^{(RO)2}$NHC$^{iPr}$-AuNP and $^{(RO)2}$NHC$^{Et}$-AuNP.

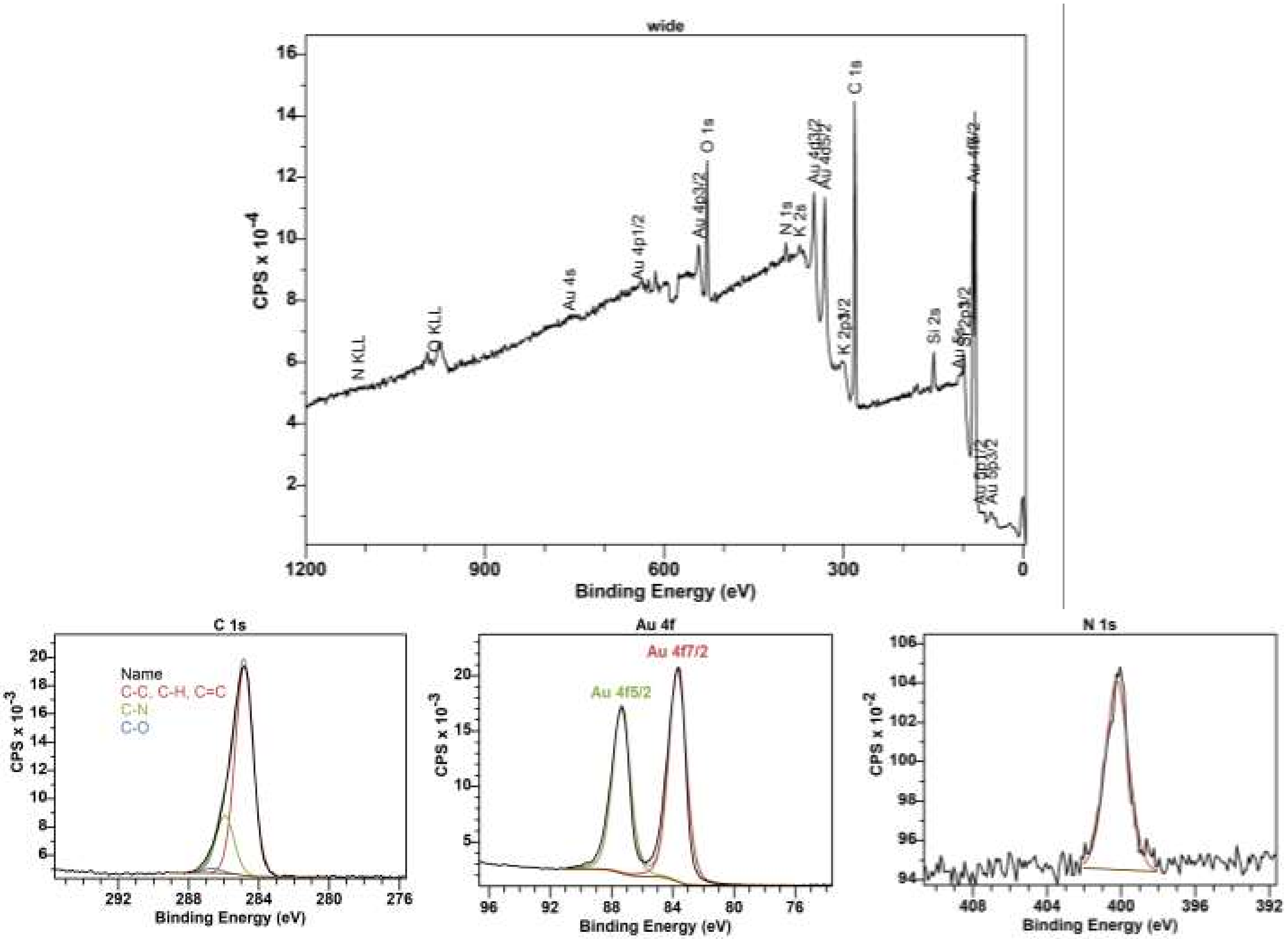


**Fig. S4.** XPS spectra of bottom-up synthesized **$^{(RO)2}$NHC$^{iPr}$-AuNP**.

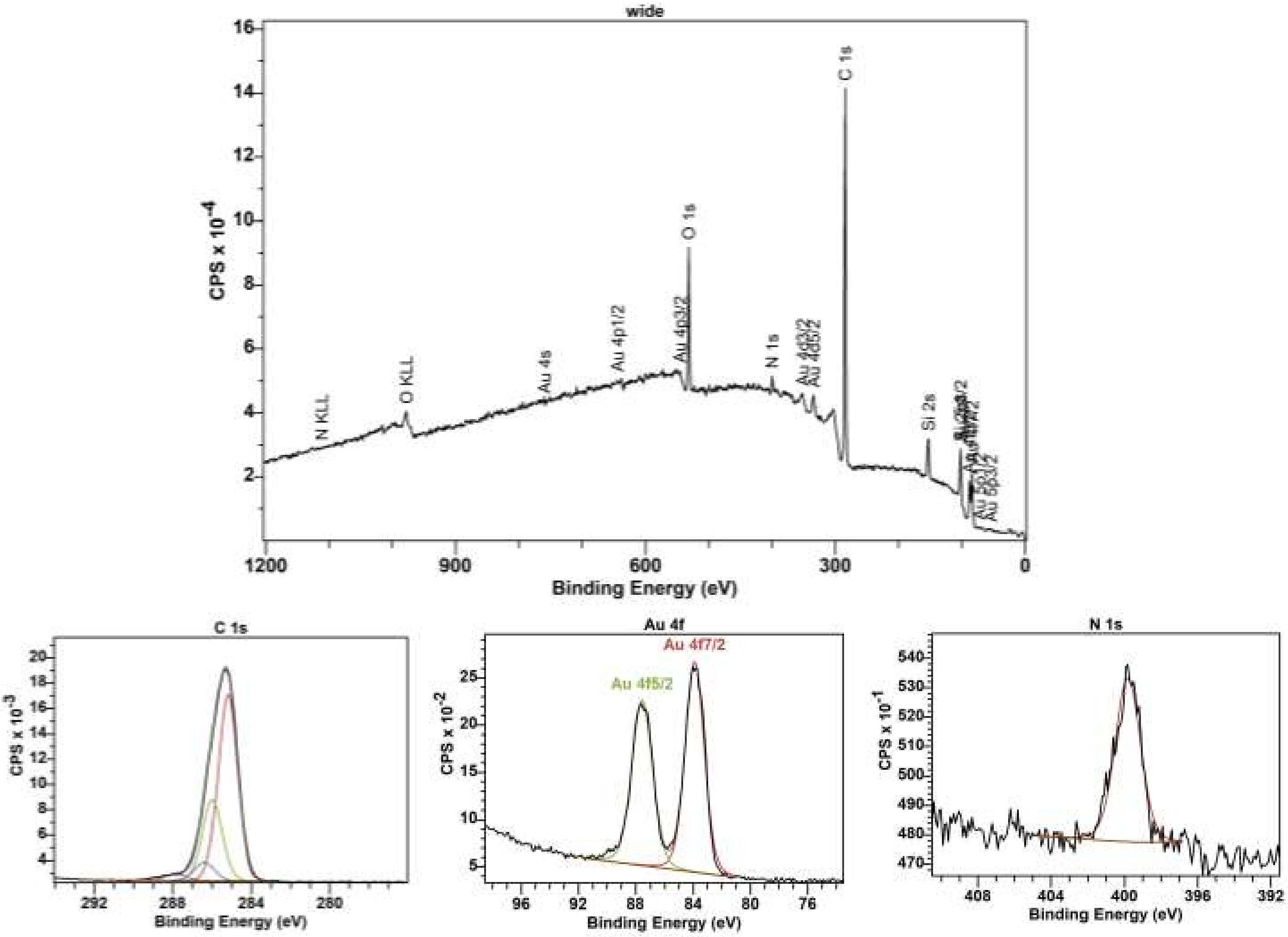


**Fig. S5.** XPS spectra of bottom-up synthesized $^{(RO)2}$**NHC**$^{Et}$**-AuNP**.

## S.3.9. Thermogravimetric analysis of NHC salts and bottom-up synthesized gold nanoparticles (AuNPs).

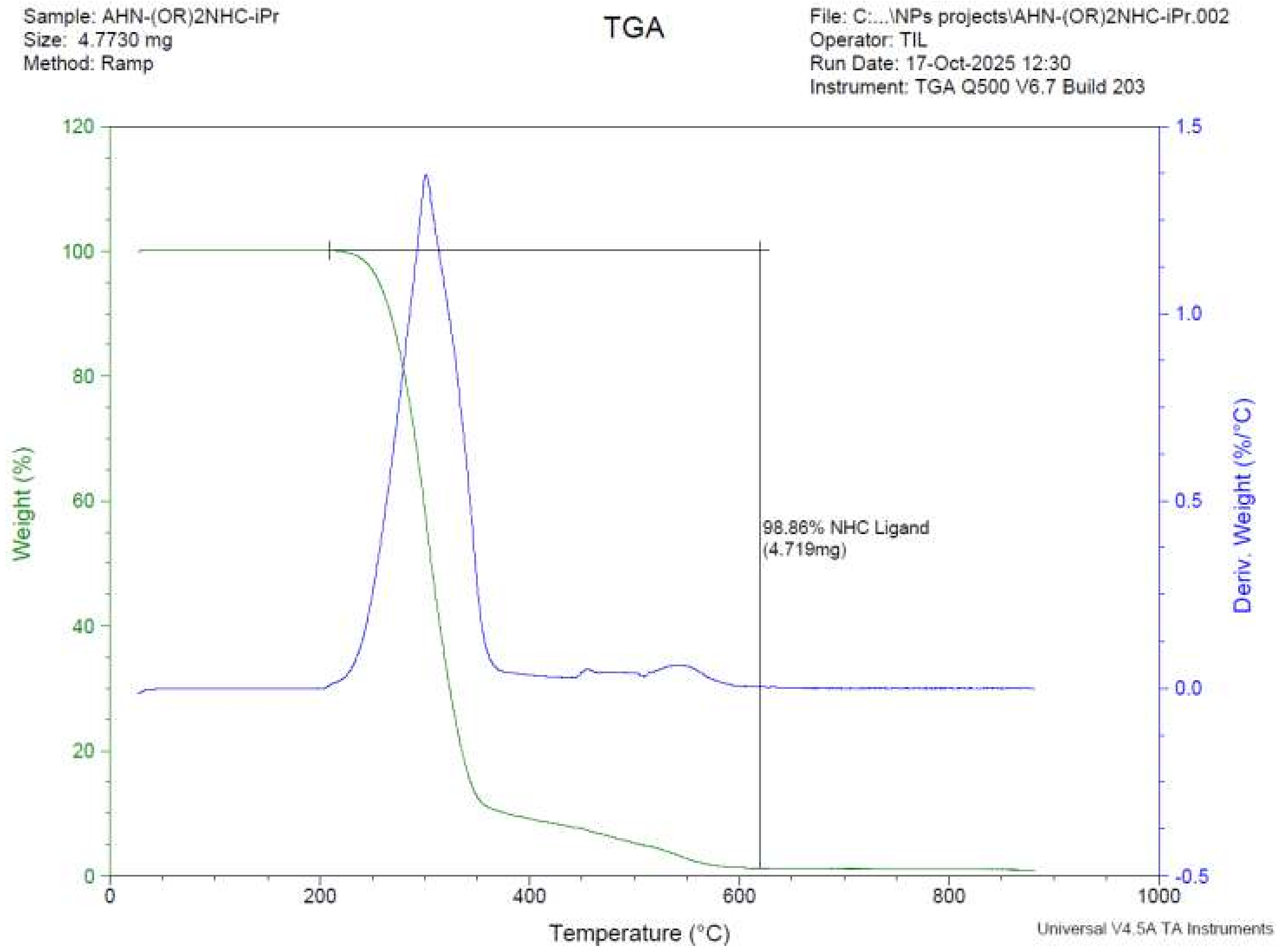


**Fig. S6.** Thermogravimetric Analysis (TGA) of **$^{(RO)2}$NHC$^{iPr}$·HBr**.

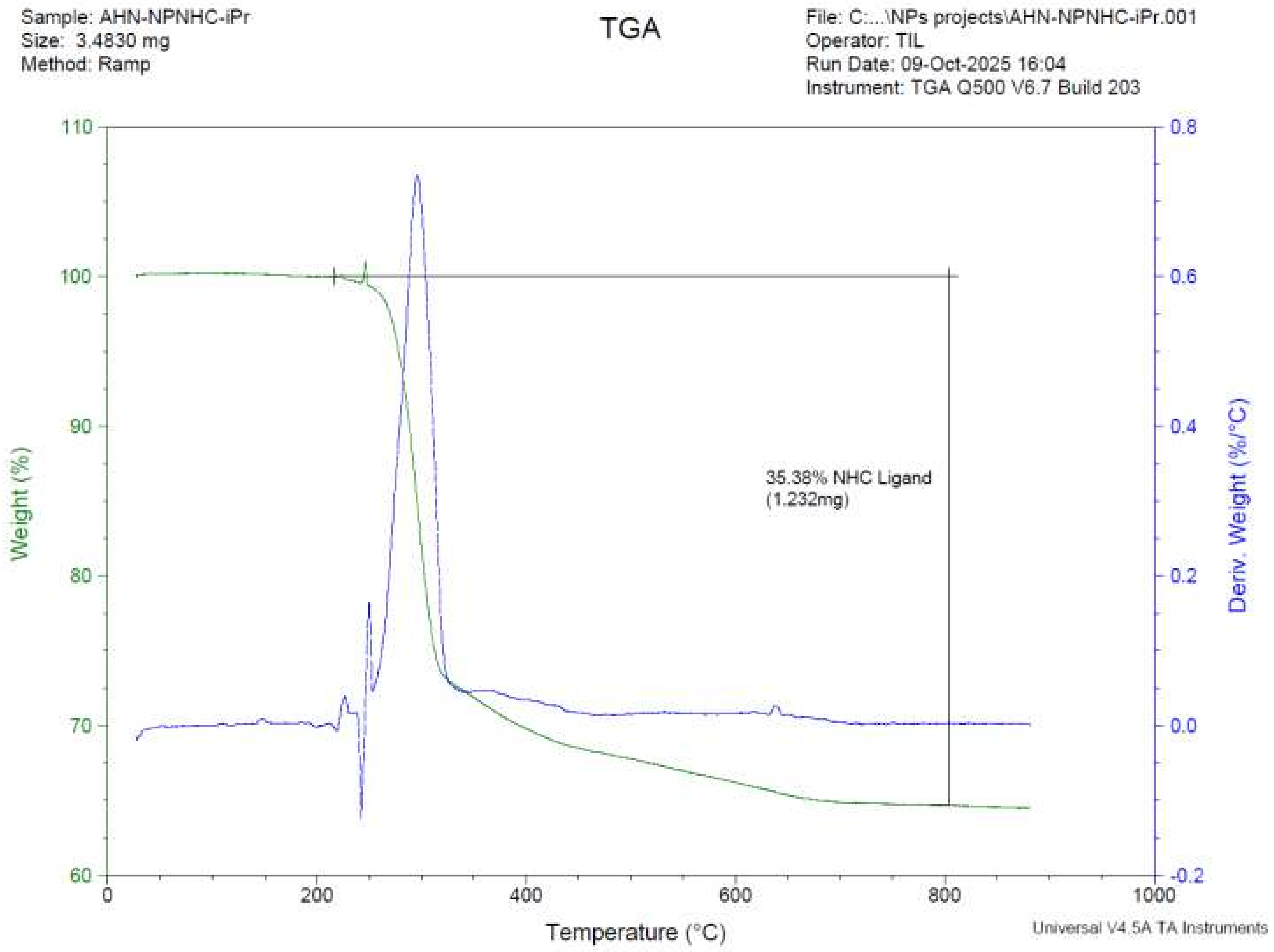


**Fig. S7.** Thermogravimetric Analysis (TGA) of bottom-up synthesized **$^{(RO)2}$NHC$^{iPr}$-AuNP**.

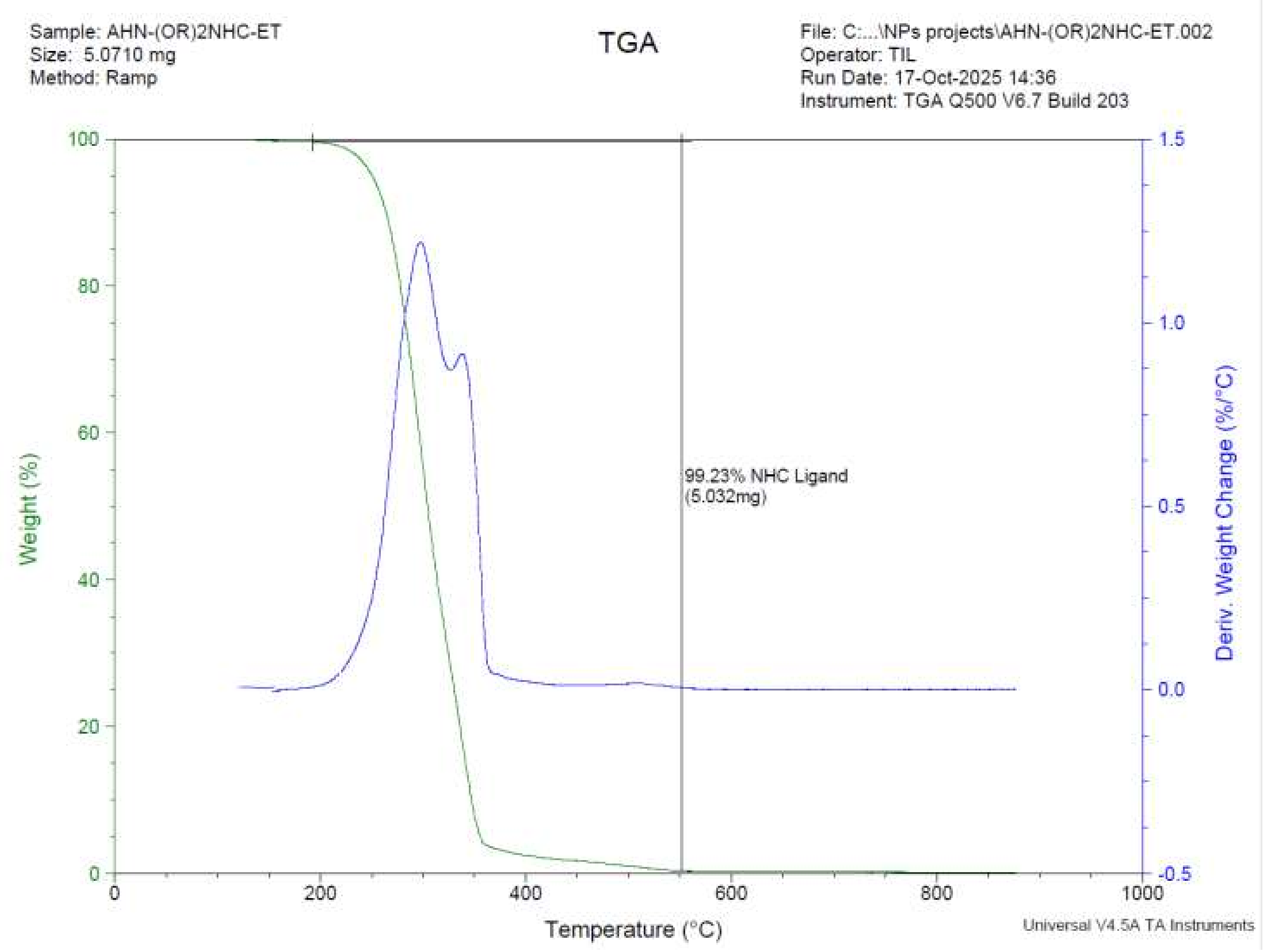


**Fig. S8.** Thermogravimetric Analysis (TGA) of **$^{(RO)2}$NHC$^{Et}$·HBr**.

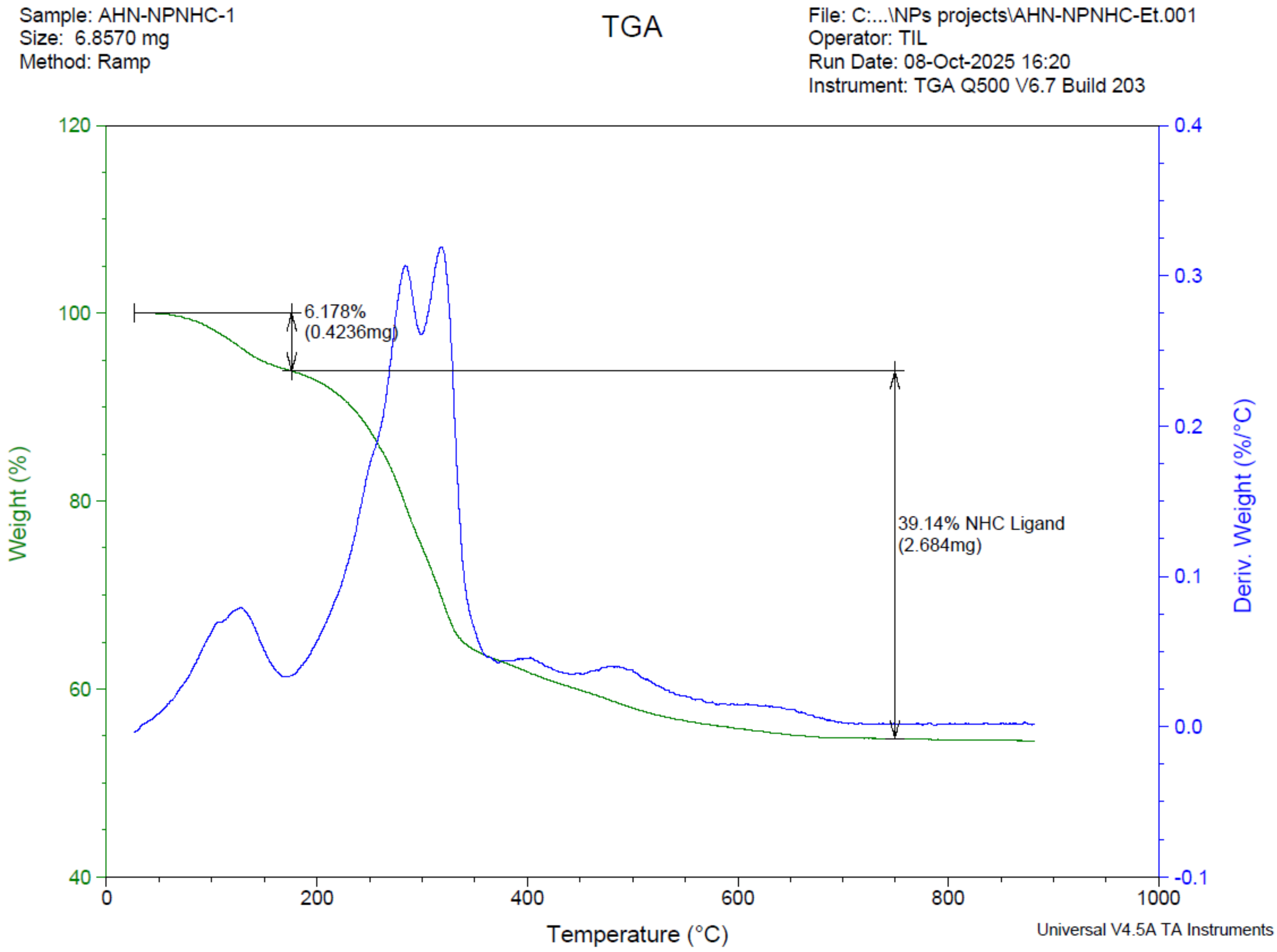


**Fig. S9.** Thermogravimetric Analysis (TGA) of bottom-up synthesized $^{(RO)2}$**NHC$^{Et}$-AuNP**.

## S.3.10. SER spectra for the upright and flat NHC$^{iPr}$ configurations on $Au_2$ and $Au_{58}$ clusters

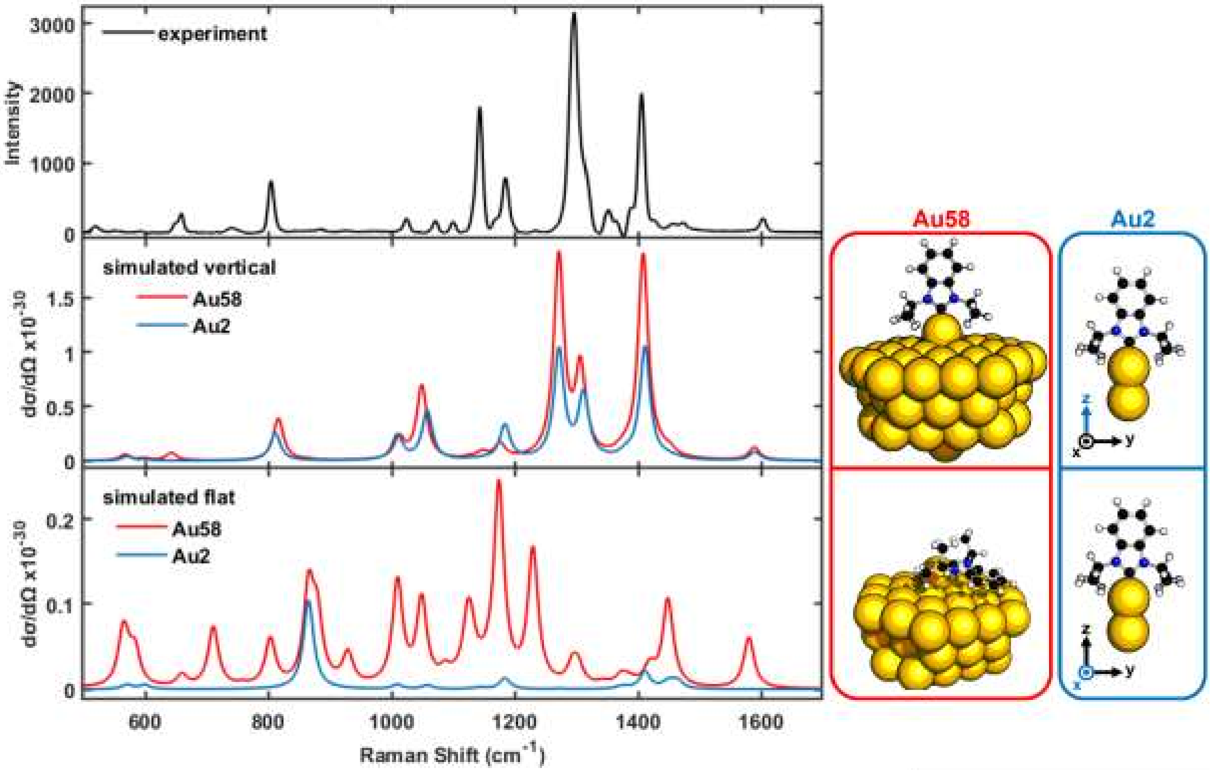


**Fig. S10.** A comparison of the SER spectra for the upright and flat NHC$^{iPr}$ configurations on $Au_2$ and $Au_{58}$ clusters.

## S.3.11. TEM images of (a) citrate-capped gold NPs, (b) top-down synthesized $^{(RO)2}$NHC$^{iPr}$-AuNP, and (c) top-down synthesized $^{(RO)2}$NHC$^{Et}$-AuNP.

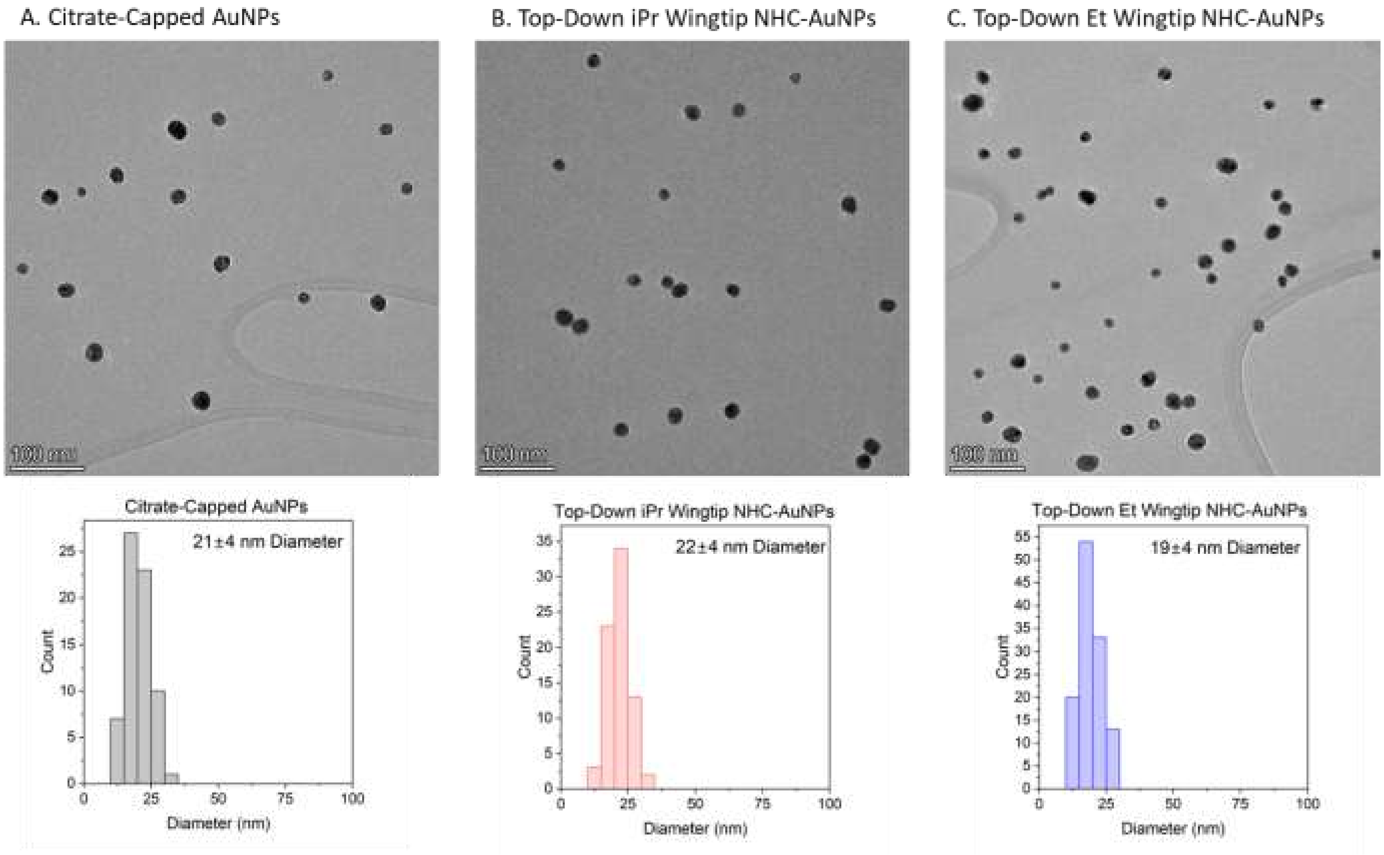


**Fig. S11.** TEM images of (a) citrate-capped gold NPs, (b) top-down synthesized **$^{(RO)2}$NHC$^{iPr}$-AuNP**, and (c) top-down synthesized **$^{(RO)2}$NHC$^{Et}$-AuNP** and their accompanying size distribution histograms with the average ± standard deviation. The nanoparticle diameter was calculated by first using imageJ to measure the particle area, then calculating the diameter assuming circular nanoparticles. No differences in nanoparticle size or morphology were observed between these three groups within experimental error.

## S.3.12. XPS spectra of top-down synthesized $^{(RO)2}$NHC$^{iPr}$-AuNP and $^{(RO)2}$NHC$^{Et}$-AuNP.

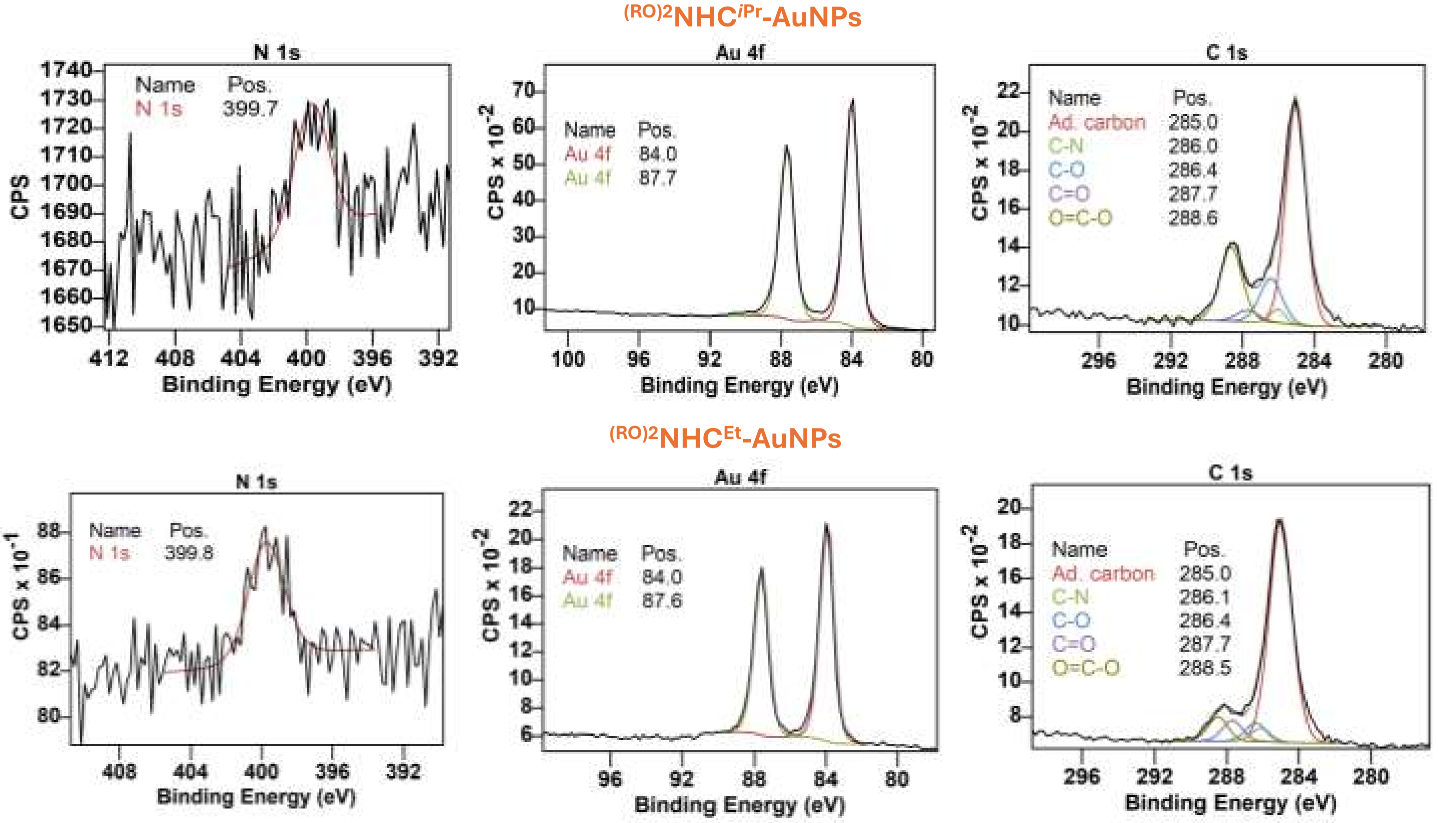


**Fig. S12.** XPS spectra of **$^{(RO)2}$NHC$^{iPr}$-AuNP** (top) and **$^{(RO)2}$NHC$^{Et}$-AuNP** (bottom) showing the N 1s and Au 4f regions

## S.3.13. Orientation of $^{(MeO)2}$NHC$^{iPr}$ on gold flat surfaces

To confirm that the SER signatures we observed were not a consequence of the NHC deposition procedure, we used SERS to compare the orientation of **$^{(MeO)2}$NHC$^{iPr}$**, which is structurally analogous to **$^{(RO)2}$NHC$^{iPr}$**, on gold NP and gold flat surfaces. To do so, **$^{(MeO)2}$NHC$^{iPr}$•$H_2CO_3$** salt and depositing this onto a flat gold surface using a well-established literature procedure.[11,21–26] Gold mirrors were prepared according to a previously reported procedures described elsewhere.[11,24,27] Briefly, approximately 5 nm of chromium and then 100 nm of gold were deposited onto a piranha acid (caution, extremely dangerous, never store) etched glass slide using a Kurt J. Lesker Physical Vapor Deposition System (Nano36, Kurt J. Lesker, Jefferson Hills, PA).

To deposit the **$^{(MeO)2}$NHC$^{iPr}$•$H_2CO_3$** onto the gold mirror, a modified literature protocol was followed.[11] 25 by 25 mm Gold mirrors were cut into quarters and rinsed with ultrapure water (>18 MOhms) and reagent alcohol (VWR). Then, 20 microliters of 10 mM NHC bicarbonate salt in methanol (Sigma Aldrich, Emprove) was deposited onto the gold mirror substrate, which was allowed to dry under ambient conditions. Then, the mirrors were annealed in

a vacuum oven (National Appliance) at 104 degrees Celsius and <5 Torr. After annealing, the samples were removed from the oven and rinsed twice with reagent alcohol. Fig. S13 compares the SER signatures for top-down and bottom-up preparations of **$^{(RO)2}$NHC$^{iPr}$-AuNP** with those prepared from bicarbonate salt deposition. In each case, the vibrational signatures are qualitatively the same, illustrating that comparable NHC surface structures form regardless of the deposition procedure.

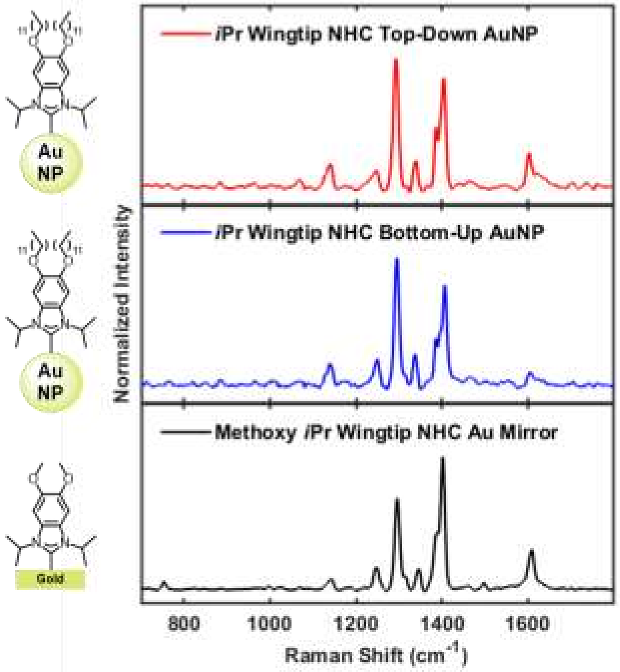


**Fig. S13.** SER spectra of (a) top-down synthesized **$^{(RO)2}$NHC$^{iPr}$-AuNP** (top), (b) bottom-up synthesized **$^{(RO)2}$NHC$^{iPr}$-AuNP** (middle), and (c) **$^{(MeO)2}$NHC$^{iPr}$** deposited on a gold mirror substrate (bottom).

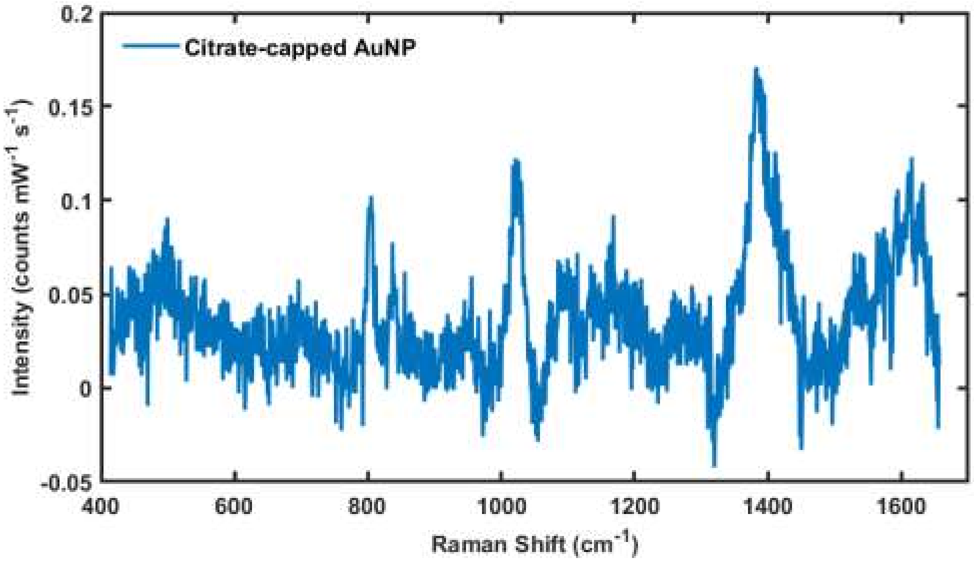


**Fig. S14.** SER spectra of Citrate-capped AuNP

## S.3.14. XPS spectra of top-down synthesized $^{RO}$NHC$^{iPr}$-AuNP and $^{RO}$NHC$^{Et}$-AuNP.

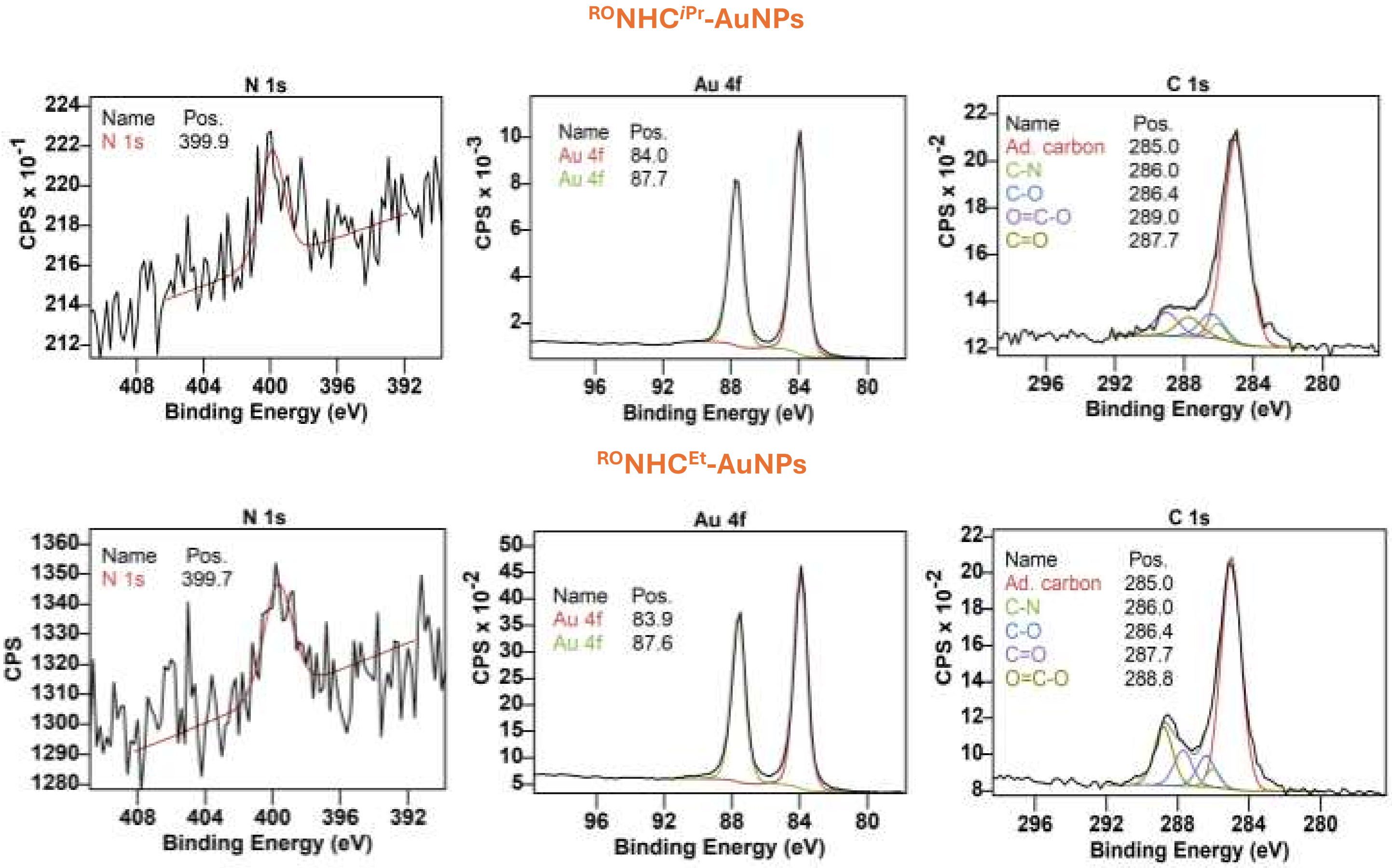


**Fig. S15.** XPS spectra of **$^{RO}$NHC$^{iPr}$-AuNP** (top) and **$^{RO}$NHC$^{Et}$-AuNP** (bottom) showing the N 1s and Au 4f regions

# S.3.15. STM Data

Our analysis of the observed lattice was performed using larger-area, drift-corrected STM images (Fig. S16a). By applying image thresholding techniques, we extracted the mean positions of the central bright protrusions corresponding to Au adatoms. From these coordinates, we analyzed pairwise distance and angle distributions to quantify the relative displacements within the adatom lattice. To determine angles relative to the crystallographic directions of the Au(111) substrate, we compared the orientation of the overlayer to atomically resolved STM images of the clean Au(111) surface. Based on this analysis, we typically assign a matrix model for the overlayer by best matching the measured distances and angles to ideal point adsorption sites on a pristine (111) surface. However, here a precise prescription of the overlayer's registration with the surface is prevented by i) the underlying herringbone reconstruction which persists, evident by an ~20 pm contrast modulation still observed in STM (Fig. S16b), and ii) slight deviations in positions by seemingly random changes in NHC complex isomers along the $[1\bar{1}0]$ direction (Fig. S16a). Instead, the approximate lattice parameters of the adatom lattice, extracted from the data shown in Fig. S14c are given.

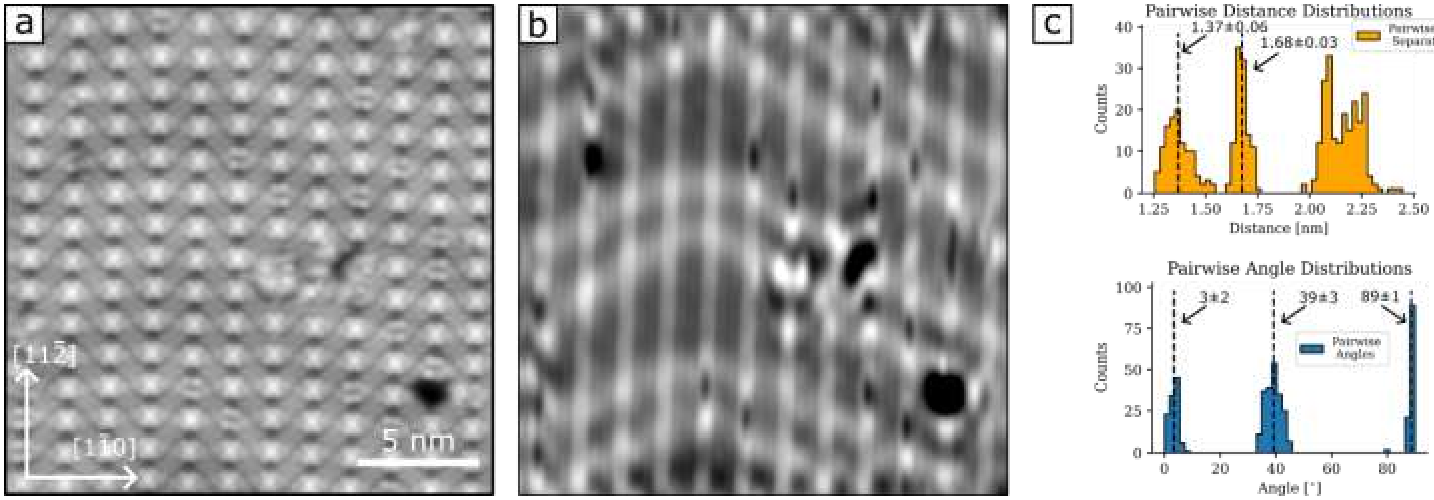


**Fig. S16.** **$^{RO}$NHC$^{Et}$** overlayer characterization. a) Larger scale STM images of the **$^{RO}$NHC$^{Et}$** complex self-assembly phase, from which statistics on adatom positions were extracted (20 nm x 20 nm, 20 pA, 100 mV). b) Same as in a, with higher spatial frequency components filtered out to illustrate the contrast modulation due to the underlying surface reconstruction. c) [top]: pairwise distance distribution of the mean adatom positions extracted form (a). Bin width of the histogram is chosen to match the physical pixel size of the image. [bottom]: pairwise angle distributions between adatom positions. Angles given are counterclockwise relative to the [1$\bar{1}$0] direction. For both histograms, dashed vertical lines represent the mean position of each distinct peak.

Additional STM experiments were conducted to further understand how alkyl chain length may affect the adsorption geometries of NHCs on planar surfaces. To do so, overlayers of **$^{RO}$NHC$^{Et}$** (R = n-dodecyl), were prepared on Au(111), resulting in Fig. S17. Again, we observe the formation of flat-lying bis-(NHC)$_2$Au complexes from **$^{RO}$NHC$^{Et}$** evinced by the characteristic bright protusions corresponding the the central Au atom, flanked on either side by three lobed structures corresponding to the benzimidazole backbone and ethyl wingtip groups. Details attributable to the alkyl chain groups are also resolved. While the exact atomic arrangement of the chains is not resolved, we suspect the chains may slightly buckle in and out of the surface plane. This provides a plausible explanation for the bright defects observed and is supported by the ~1.5 nm chain length extracted from line profile measurements. Furthermore, additional steric interactions between the longer chains likely results in the lack of

translational symmetry observed in the pseudo-ordered domains.

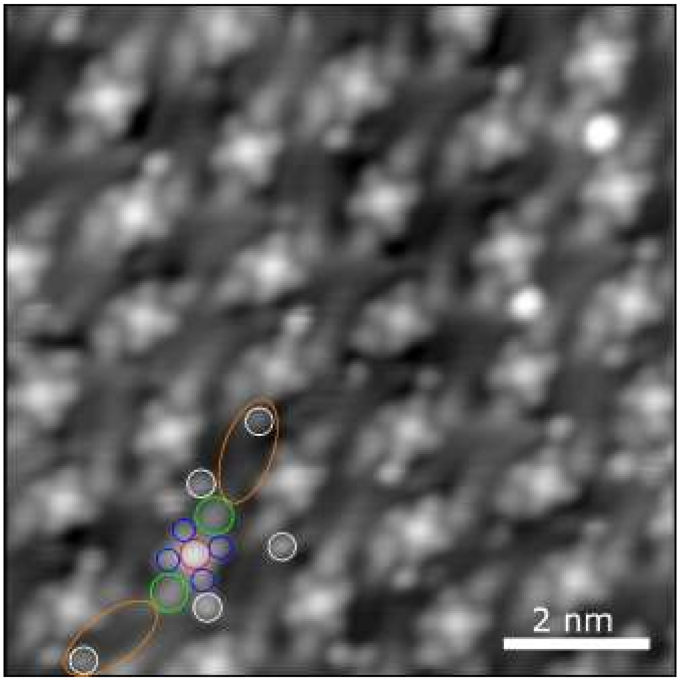


**Fig. S17.** Constant current STM image of the $^{\mathbf{RO}}\mathbf{NHC}^{\mathbf{Et}}$ (R = n-dodecyl) overlayer formed following the vapor phase deposition onto Au(111) (10 nm × 10 nm, 100 mV, 20 pA). The annotated red, blue and green circles indicate the position of an Au adatom, wingtip groups, and benzimidazole backbones respectively. Orange ellipses and white circles respectively indicate alkyl chains and bright defects attributed to out of plane buckling.

## S.3.16. TEM images of bottom-up synthesized $^{(RO)2}$NHC$^{iPr}$-AuNP and $^{(RO)2}$NHC$^{Et}$-AuNP.

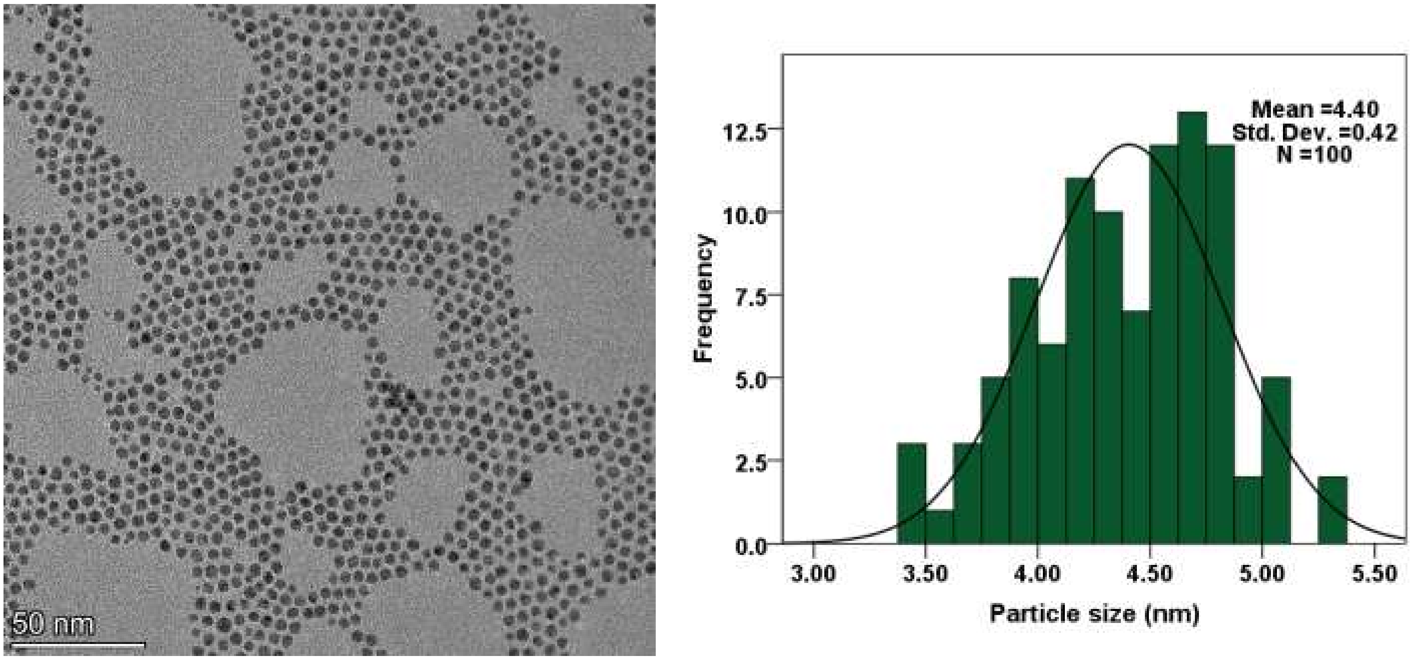


**Fig. S18.** TEM image of bottom-up synthesized **$^{(RO)2}$NHC$^{iPr}$-AuNP** obtained at r.t.

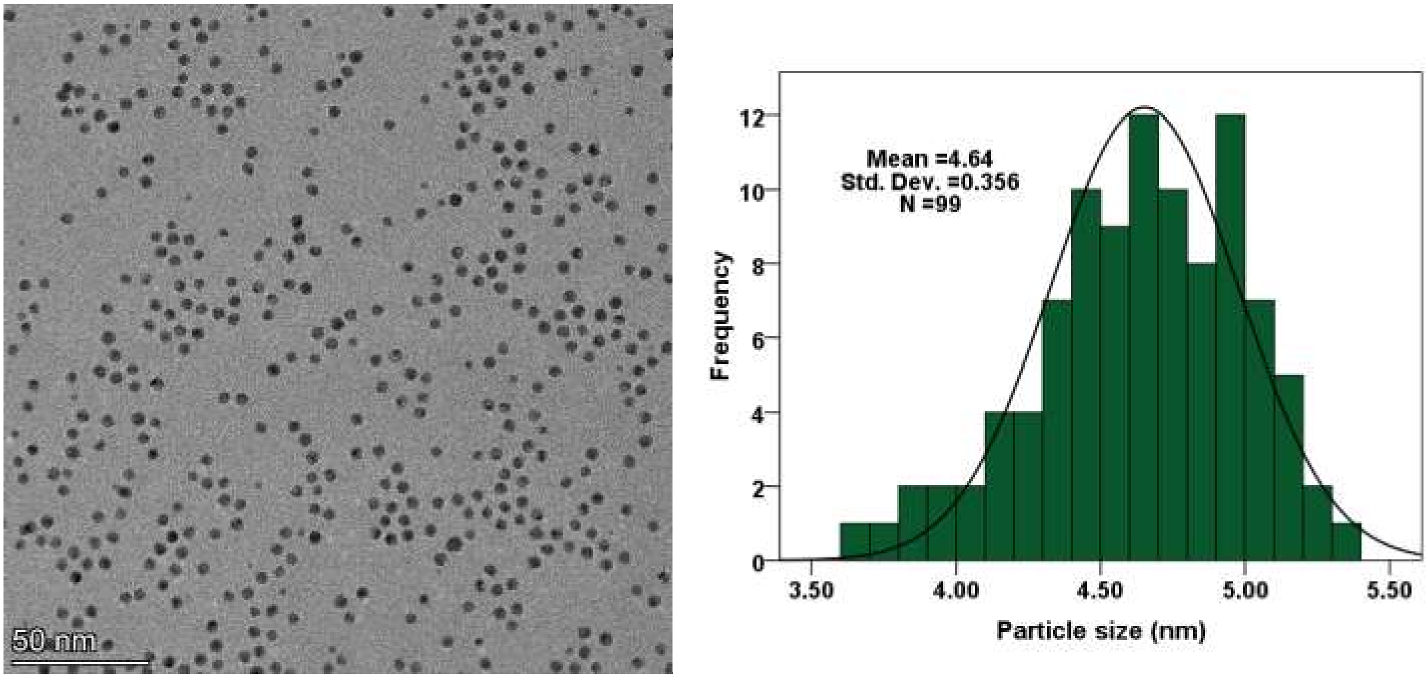


**Fig. S19.** TEM image of bottom-up synthesized **$^{(RO)2}$NHC$^{iPr}$-AuNP** after heating at 70 °C.

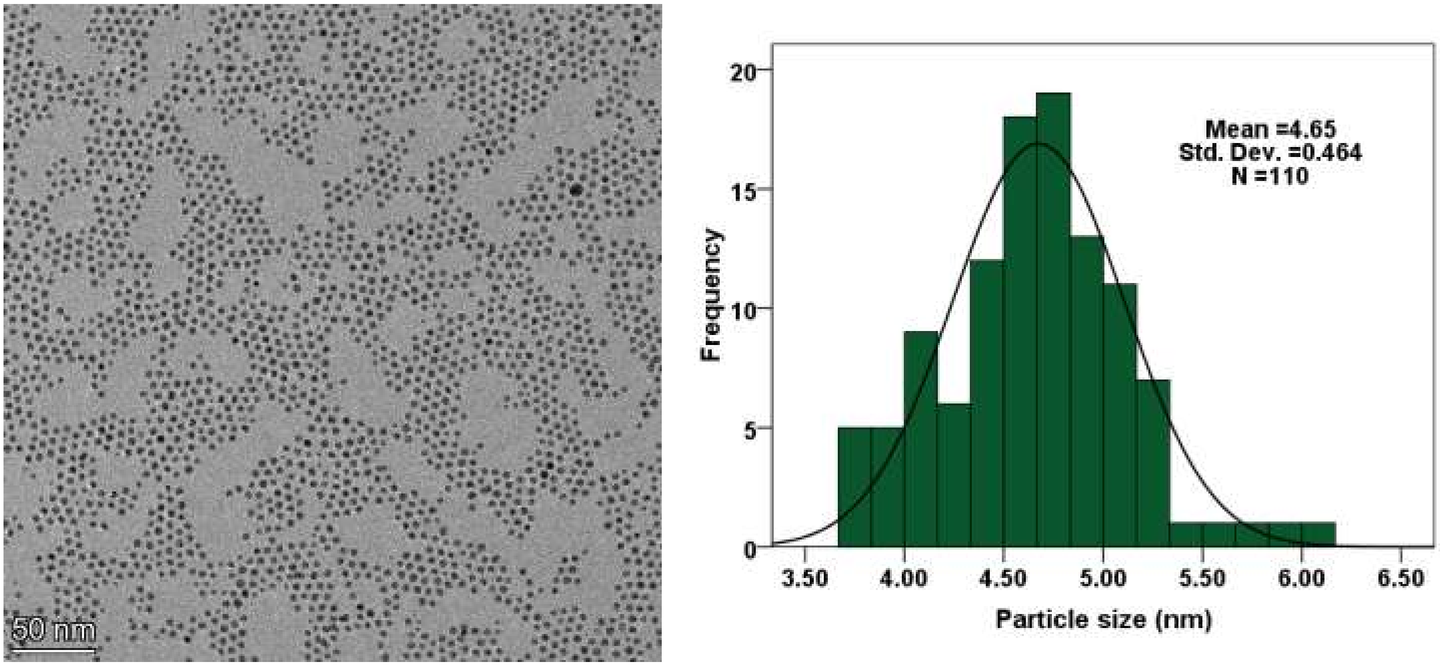


**Fig. S20.** TEM image of bottom-up synthesized $^{(RO)2}$**NHC**$^{iPr}$**-AuNP** after heating at 100 ℃ for 24 h.

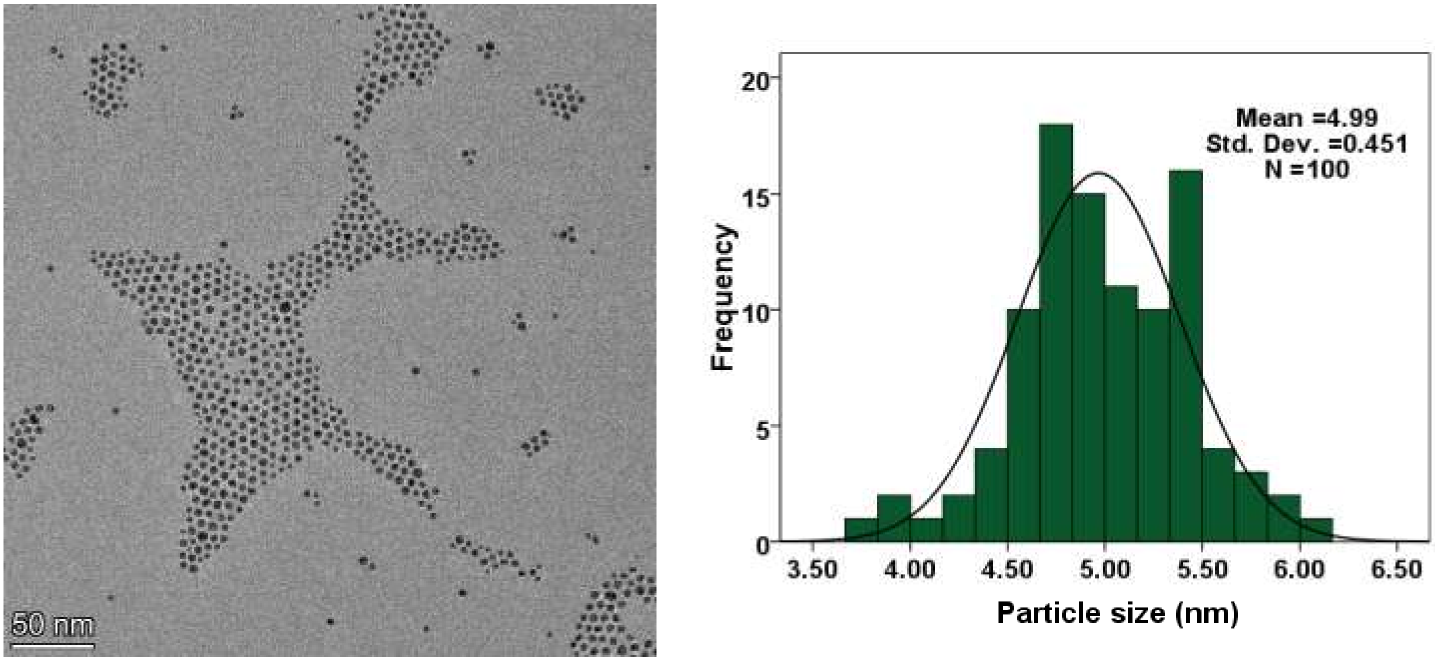


**Fig. S21.** TEM image of bottom-up synthesized $^{(RO)2}$**NHC**$^{iPr}$**-AuNP** after heating at 100 ℃ for 72 h.

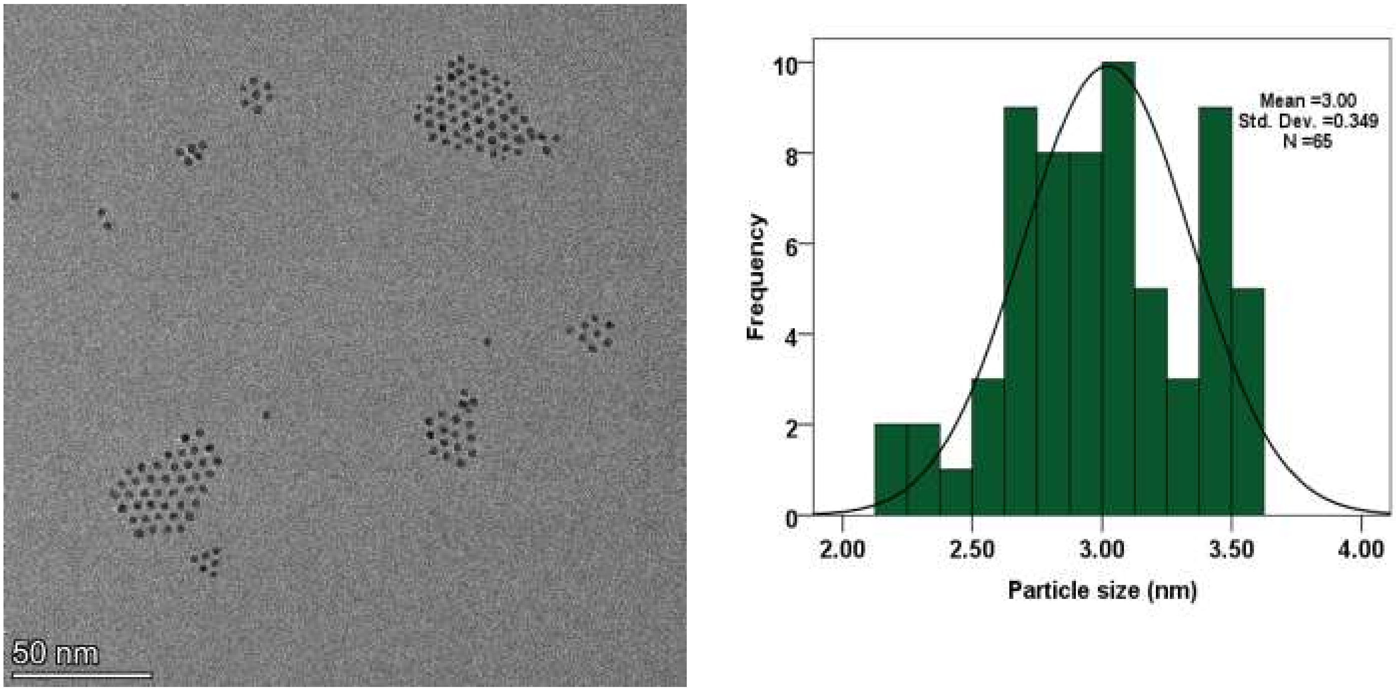


**Fig. S22.** TEM image of bottom-up synthesized **$^{(RO)2}$NHC$^{Et}$-AuNP** obtained at r.t.

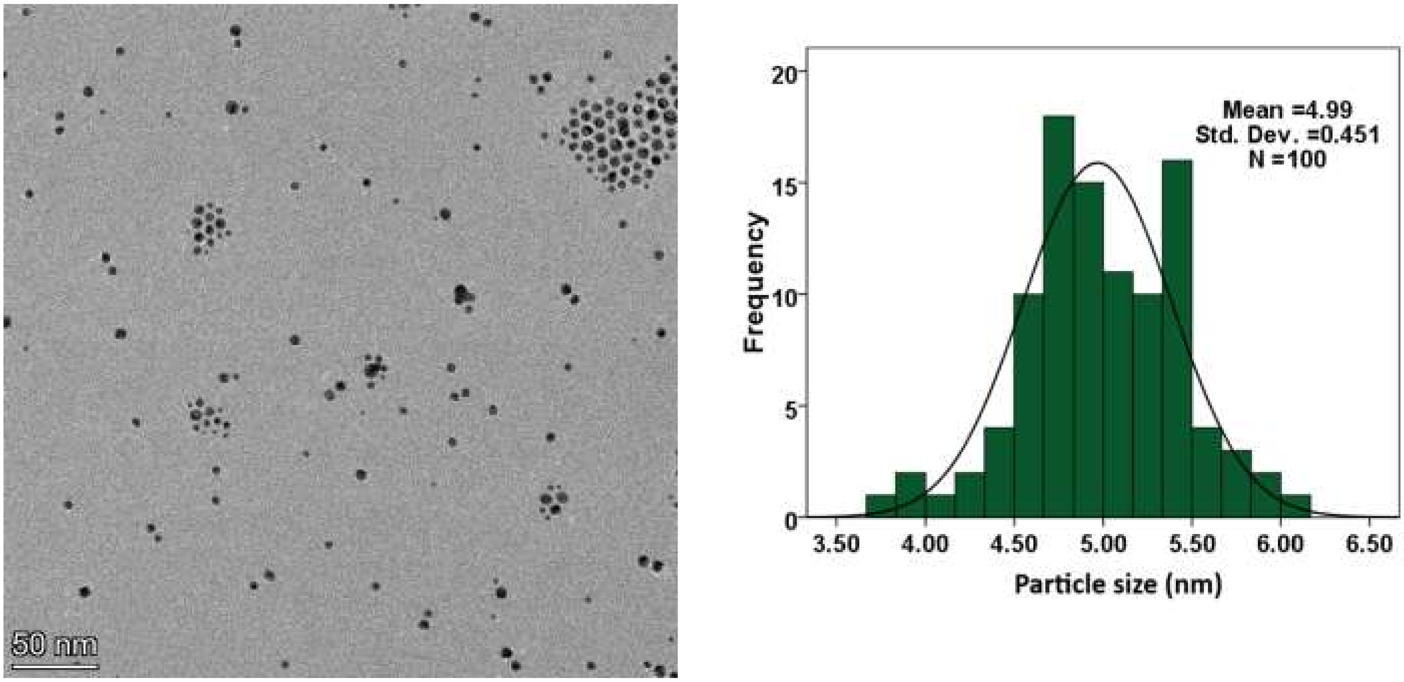


**Fig. S23.** TEM image of bottom-up synthesized **$^{(RO)2}$NHC$^{Et}$-AuNP** after heating at 70 °C.

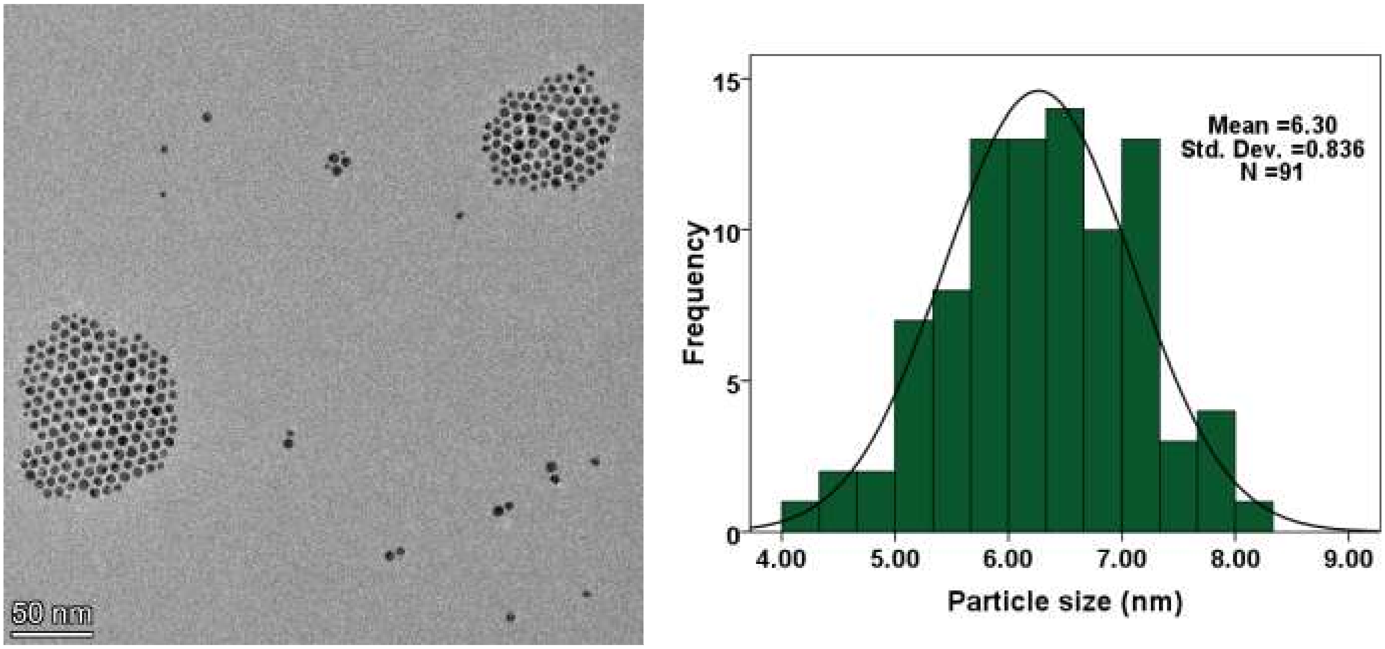


**Fig. S24.** TEM image of bottom-up synthesized **$^{(RO)2}$NHC$^{Et}$-AuNP** after heating at 100 ℃ for 24 h.

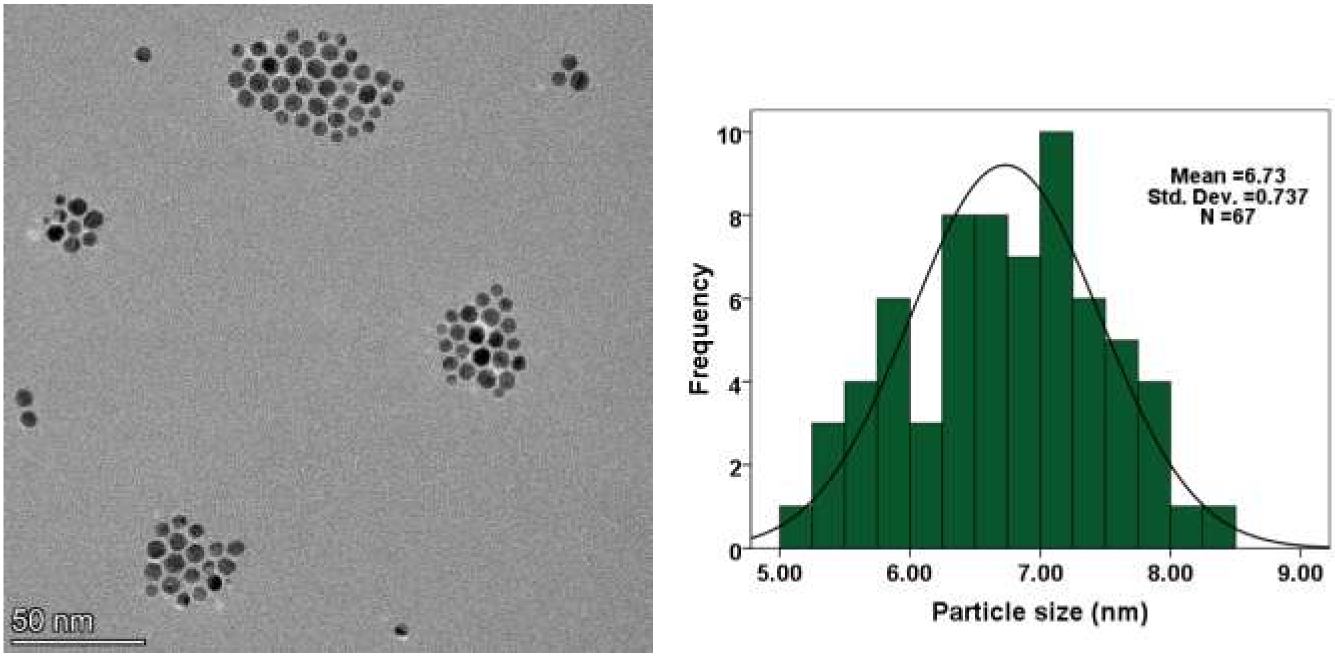


**Fig. S25.** TEM image of bottom-up synthesized **$^{(RO)2}$NHC$^{Et}$-AuNP** after heating at 100 ℃ for 72 h.

## S.3.17. Thermal stability data of top-down synthesized AuNPs.

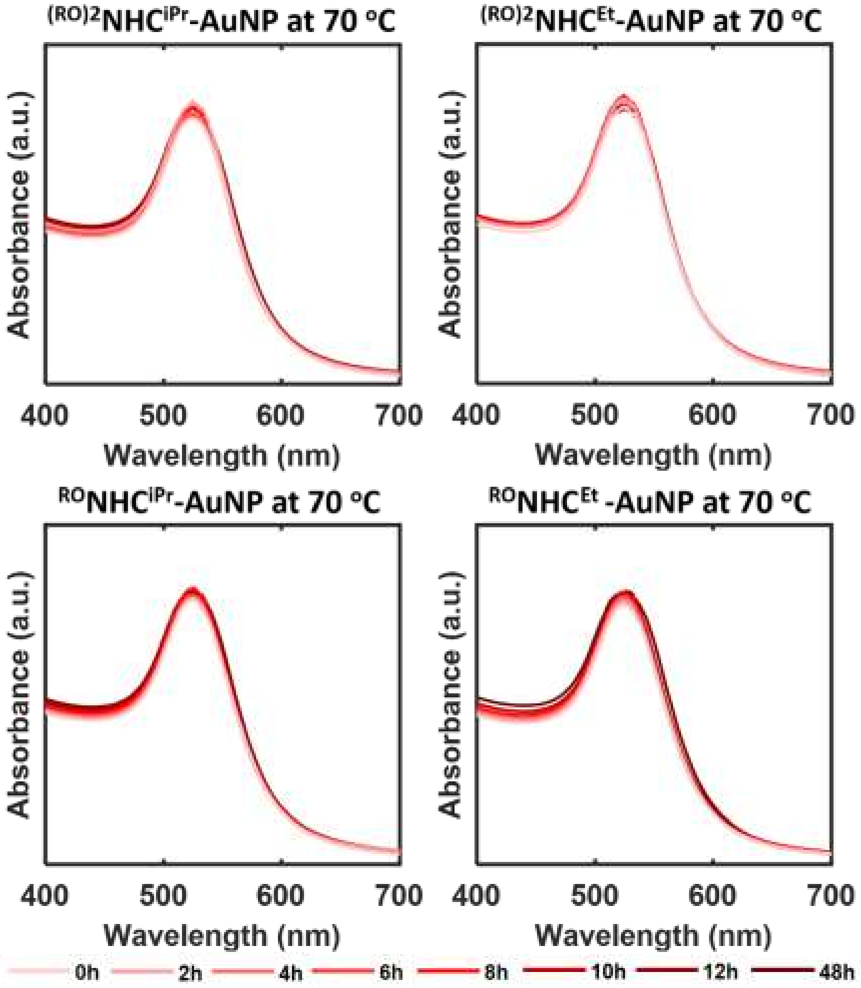


**Fig. S26.** Thermal stability of top-down synthesized **$^{(RO)2}$NHC$^{iPr}$-**, **$^{(RO)2}$NHC$^{Et}$-**, **$^{RO}$NHC$^{iPr}$-**, and **$^{RO}$NHC$^{Et}$-AuNPs** at 70 °C.

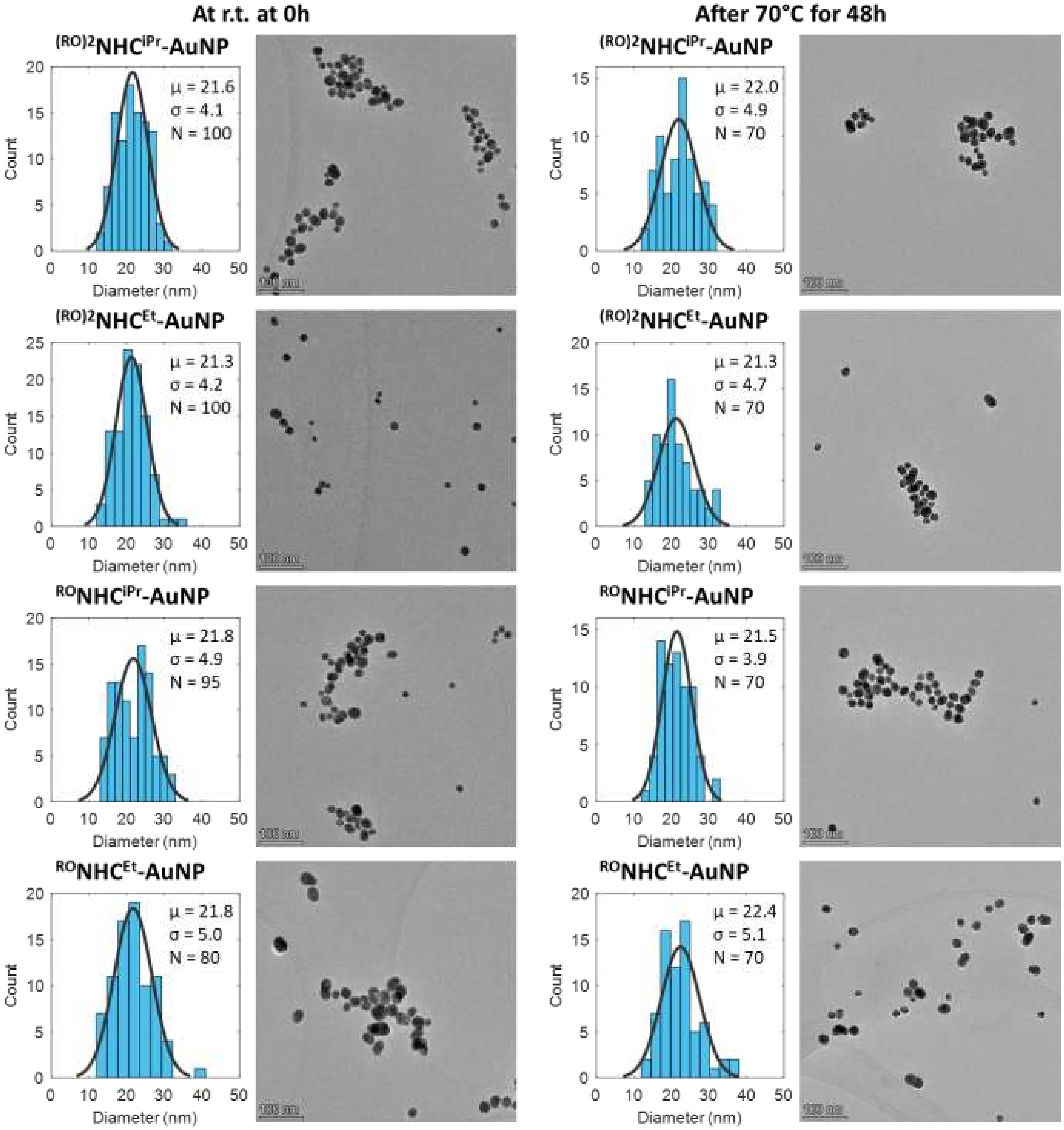


**Fig. S27**. TEM images of top-down synthesized **$^{(RO)2}$NHC$^{iPr}$-**, **$^{(RO)2}$NHC$^{Et}$-**, **$^{RO}$NHC$^{iPr}$-**, and **$^{RO}$NHC$^{Et}$-AuNPs** before and after heating at 70 °C for 48 h, and their corresponding size distribution histograms with the mean (μ) and standard deviations (σ). No differences in nanoparticle size or morphology were observed between them within experimental error.

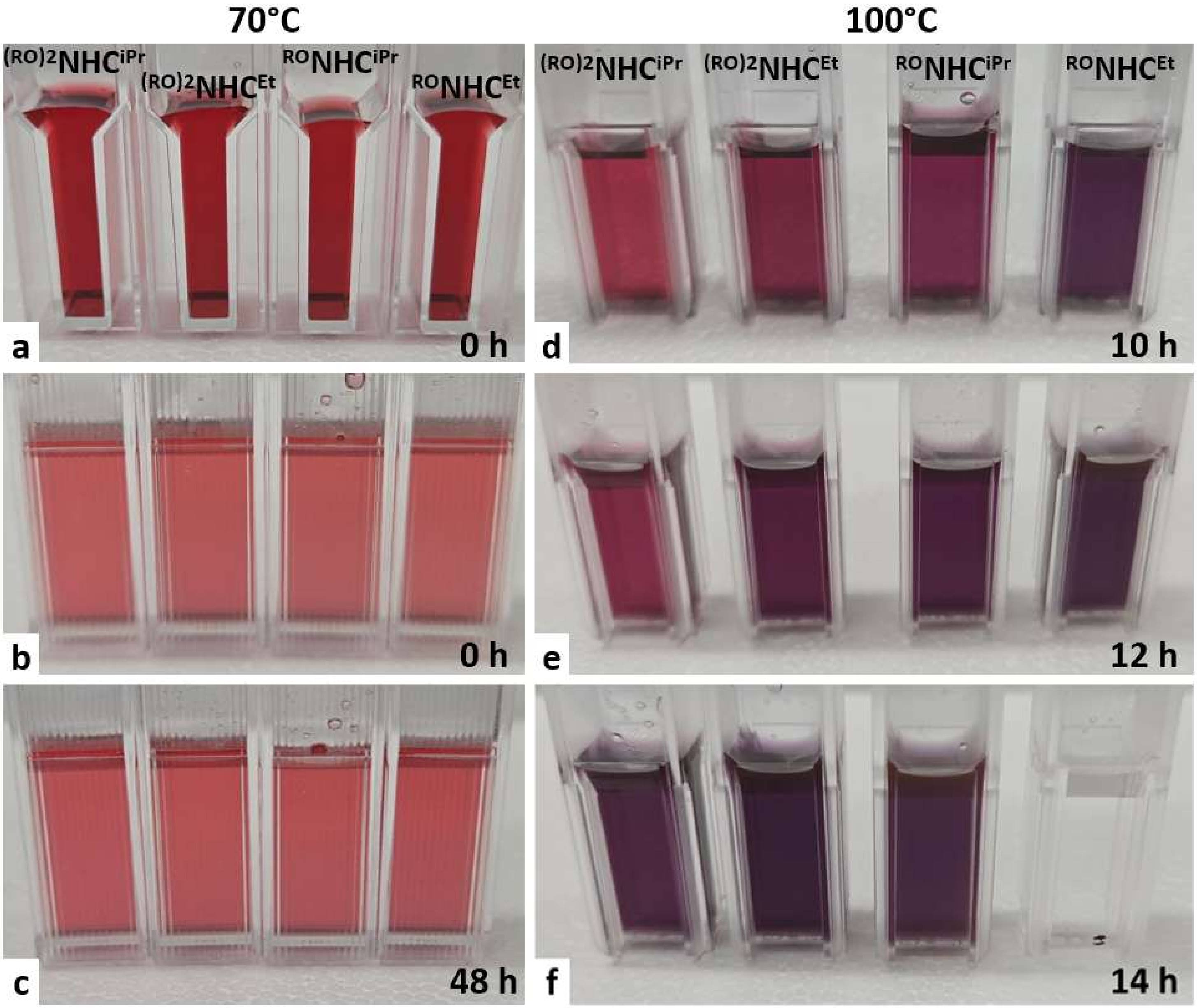


**Fig. S28.** Solutions of top-down synthesized **$^{(RO)2}$NHC$^{iPr}$-**, **$^{(RO)2}$NHC$^{Et}$-**, **$^{RO}$NHC$^{iPr}$-**, and **$^{RO}$NHC$^{Et}$-AuNPs** in 1.5 mL cuvettes, labelled in the figure. Initial color at 0 hour (a), same cuvettes turned 90° clockwise to make the colors more discernible (b). Changes after heating them in a 70 °C oil bath for 48 hours (c), and a 100 °C oil bath for 10 hours (d), 12 hours (e), and 14 hours (f).

## S.3.18. Comparative study on the thermal stability of bottom-up synthesized $^{(RO)2}$NHC$^{iPr}$-AuNP.

Considering the high stability of **$^{(RO)2}$NHC$^{iPr}$-AuNP**, we compared this NP with previously reported NHC-supported NPs **$^{R}$NHC$^{iPr}$-AuNP** (Fig. S27). This NHC contains two alkyl chains attached at one point to the heterocycle. These samples were heated at 130 °C in *m*-xylene. For **$^{(RO)2}$NHC$^{iPr}$-AuNP**, this led to a complete disappearance of the SPR band in the UV−Vis spectrum and the formation of an insoluble precipitate within 4 hours, while our previously studied **$^{R}$NHC$^{iPr}$-AuNP** remained stable at this temperature for 3 days.[28] The presence of two electron donor oxygen atoms in **$^{(RO)2}$NHC$^{iPr}$-AuNP** may contribute to its lower stability. These results indicate that **$^{(RO)2}$NHC$^{iPr}$-AuNP** has greater stability than **$^{(RO)2}$NHC$^{Et}$-AuNP**, and both have significantly greater stability than dodecyl sulfide-protected gold NPs (**$^{R2S}$NHC-AuNP**, Fig. S29), which decomposed in toluene at 90 °C after just 4 hours.[28]

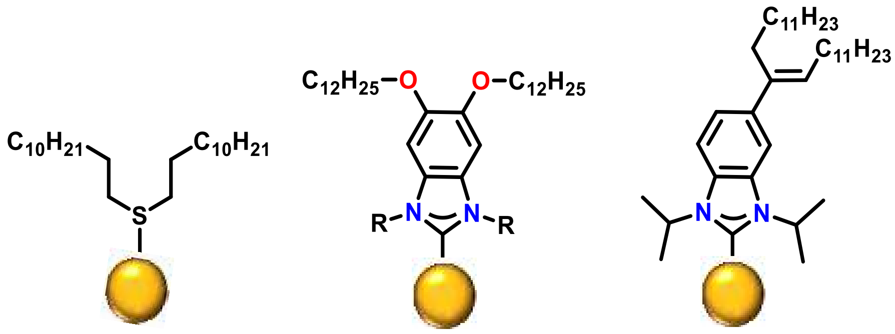


$^{R2S}$NHC-AuNP    R= = *i*Pr   $^{(RO)2}$NHC$^{iPr}$-AuNP,   $^{R}$NHC$^{iPr}$-AuNP

R= = Et   $^{(RO)2}$NHC$^{Et}$-AuNP

**Fig. S29.** AuNP supported by sulfide and NHC ligands.

## S.3.19. Zeta potential measurement data for top-down synthesized $^{(RO)2}$NHC$^{iPr}$-, $^{(RO)2}$NHC$^{Et}$-, $^{RO}$NHC$^{iPr}$-, and $^{RO}$NHC$^{Et}$-AuNPs

| | | | |
|---|---|---|---|
| **Sample Name:** | NHC-A 2 | | |
| **SOP Name:** | Ahmadreza AuNP.sop | | |
| **File Name:** | AuNP.dts | **Dispersant Name:** | Water |
| **Record Number:** | 49 | **Dispersant RI:** | 1.330 |
| **Date and Time:** | October 8, 2025 11:57:28 AM | **Viscosity (cP):** | 0.8872 |
| | | **Dispersant Dielectric Constant:** | 78.5 |

| | | | |
|---|---|---|---|
| **Temperature (°C):** | 25.1 | **Zeta Runs:** | 12 |
| **Count Rate (kcps):** | 307.8 | **Measurement Position (mm):** | 2.00 |
| **Cell Description:** | Clear disposable zeta cell | **Attenuator:** | 10 |

| | | | **Mean (mV)** | **Area (%)** | **St Dev (mV)** |
|---|---|---|---|---|---|
| **Zeta Potential (mV):** | -30.0 | **Peak 1:** | -30.0 | 100.0 | 4.98 |
| **Zeta Deviation (mV):** | 4.98 | **Peak 2:** | 0.00 | 0.0 | 0.00 |
| **Conductivity (mS/cm):** | 0.679 | **Peak 3:** | 0.00 | 0.0 | 0.00 |

**Result quality :** Good

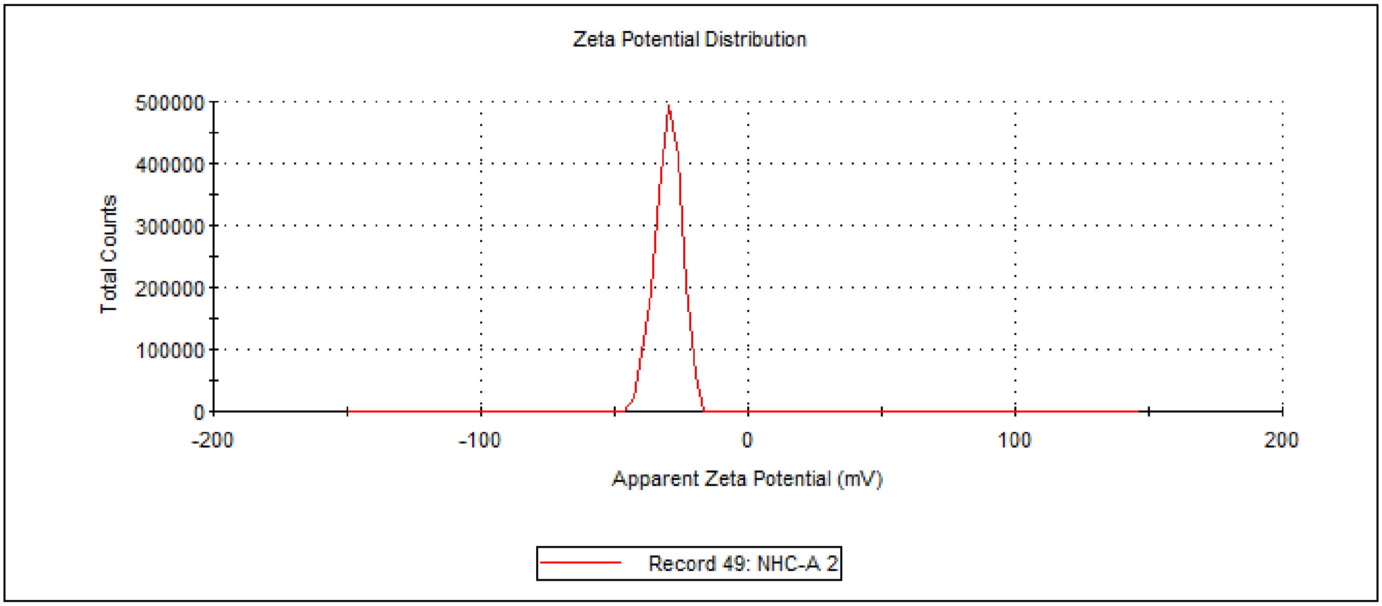


**Fig. S30.** Zeta potential measurement of top-down synthesized $^{(RO)2}$NHC$^{iPr}$-AuNPs.

**Sample Name:** NHC-B 1
**SOP Name:** Ahmadreza AuNP.sop
**File Name:** AuNP.dts
**Record Number:** 43
**Date and Time:** October 8, 2025 11:46:09 AM

**Dispersant Name:** Water
**Dispersant RI:** 1.330
**Viscosity (cP):** 0.8872
**Dispersant Dielectric Constant:** 78.5

---

**Temperature (°C):** 25.1
**Count Rate (kcps):** 144.8
**Cell Description:** Clear disposable zeta cell

**Zeta Runs:** 12
**Measurement Position (mm):** 2.00
**Attenuator:** 10

---

| | | | Mean (mV) | Area (%) | St Dev (mV) |
|---|---|---|---|---|---|
| **Zeta Potential (mV):** | -29.3 | **Peak 1:** | -29.3 | 100.0 | 5.11 |
| **Zeta Deviation (mV):** | 5.11 | **Peak 2:** | 0.00 | 0.0 | 0.00 |
| **Conductivity (mS/cm):** | 0.678 | **Peak 3:** | 0.00 | 0.0 | 0.00 |

**Result quality :** Good

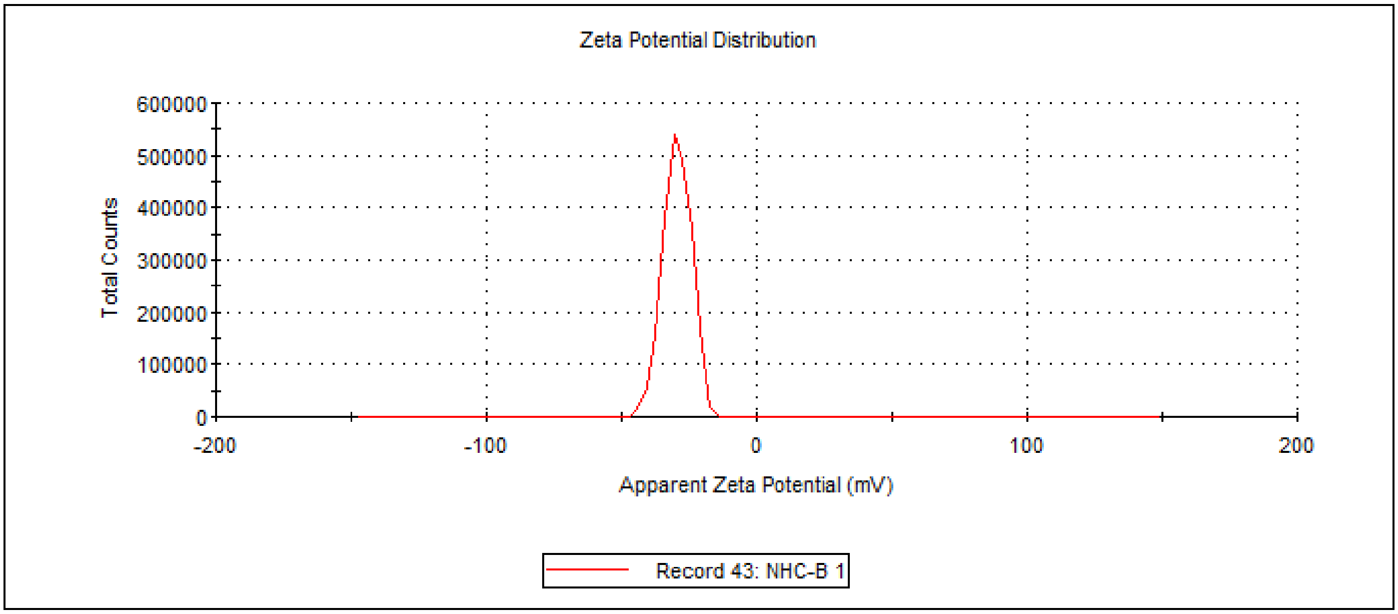


**Fig. S31.** Zeta potential measurement of top-down synthesized $^{(RO)2}$NHC$^{Et}$-AuNPs.

| | | | |
|---|---|---|---|
| **Sample Name:** | NHC-C 1 | | |
| **SOP Name:** | Ahmadreza AuNP.sop | | |
| **File Name:** | AuNP.dts | **Dispersant Name:** | Water |
| **Record Number:** | 40 | **Dispersant RI:** | 1.330 |
| **Date and Time:** | October 8, 2025 11:42:44 AM | **Viscosity (cP):** | 0.8872 |
| | | **Dispersant Dielectric Constant:** | 78.5 |

| | | | |
|---|---|---|---|
| **Temperature (°C):** | 24.9 | **Zeta Runs:** | 12 |
| **Count Rate (kcps):** | 133.1 | **Measurement Position (mm):** | 2.00 |
| **Cell Description:** | Clear disposable zeta cell | **Attenuator:** | 9 |

| | | | **Mean (mV)** | **Area (%)** | **St Dev (mV)** |
|---|---|---|---|---|---|
| **Zeta Potential (mV):** | -28.5 | **Peak 1:** | -28.5 | 100.0 | 4.60 |
| **Zeta Deviation (mV):** | 4.60 | **Peak 2:** | 0.00 | 0.0 | 0.00 |
| **Conductivity (mS/cm):** | 0.677 | **Peak 3:** | 0.00 | 0.0 | 0.00 |

**Result quality :** Good

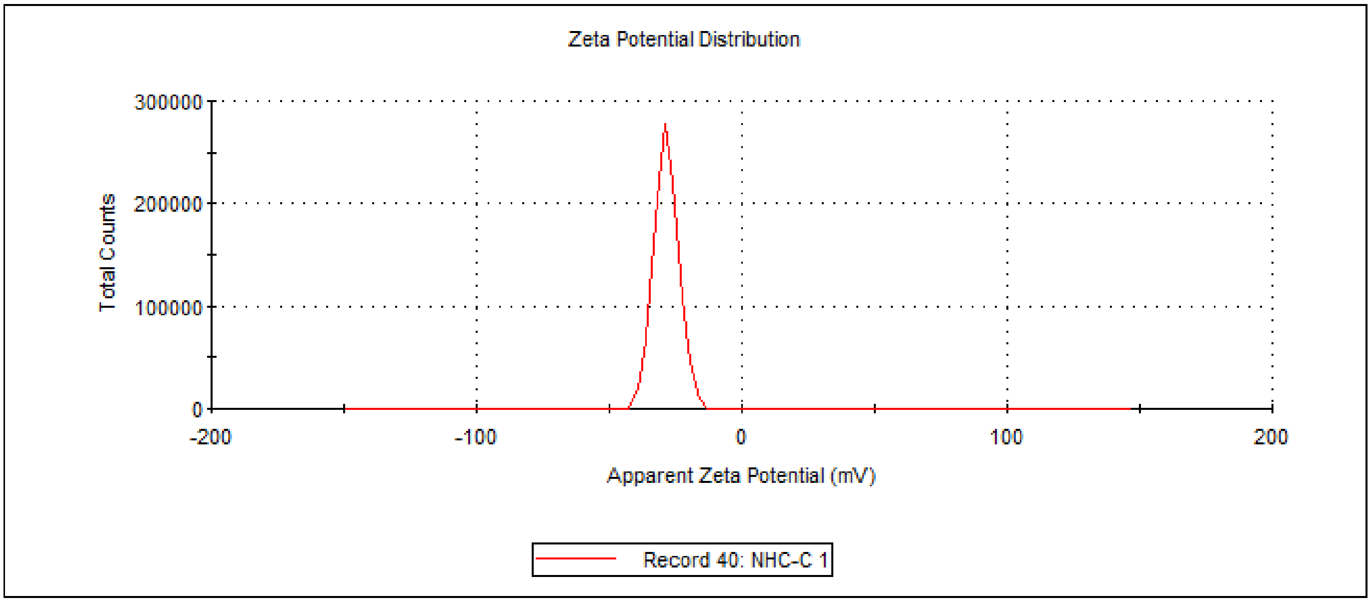


**Fig. S32.** Zeta potential measurement of top-down synthesized $^{RO}NHC^{iPr}$-AuNPs.

| | | | |
|---|---|---|---|
| **Sample Name:** | NHC-D 1 | | |
| **SOP Name:** | Ahmadreza AuNP.sop | | |
| **File Name:** | AuNP.dts | **Dispersant Name:** | Water |
| **Record Number:** | 37 | **Dispersant RI:** | 1.330 |
| **Date and Time:** | October 8, 2025 11:39:03 AM | **Viscosity (cP):** | 0.8872 |
| | | **Dispersant Dielectric Constant:** | 78.5 |

| | | | |
|---|---|---|---|
| **Temperature (°C):** | 25.0 | **Zeta Runs:** | 12 |
| **Count Rate (kcps):** | 108.6 | **Measurement Position (mm):** | 2.00 |
| **Cell Description:** | Clear disposable zeta cell | **Attenuator:** | 9 |

| | | | **Mean (mV)** | **Area (%)** | **St Dev (mV)** |
|---|---|---|---|---|---|
| **Zeta Potential (mV):** | -27.6 | **Peak 1:** | -27.6 | 100.0 | 5.04 |
| **Zeta Deviation (mV):** | 5.04 | **Peak 2:** | 0.00 | 0.0 | 0.00 |
| **Conductivity (mS/cm):** | 0.673 | **Peak 3:** | 0.00 | 0.0 | 0.00 |

**Result quality :** Good

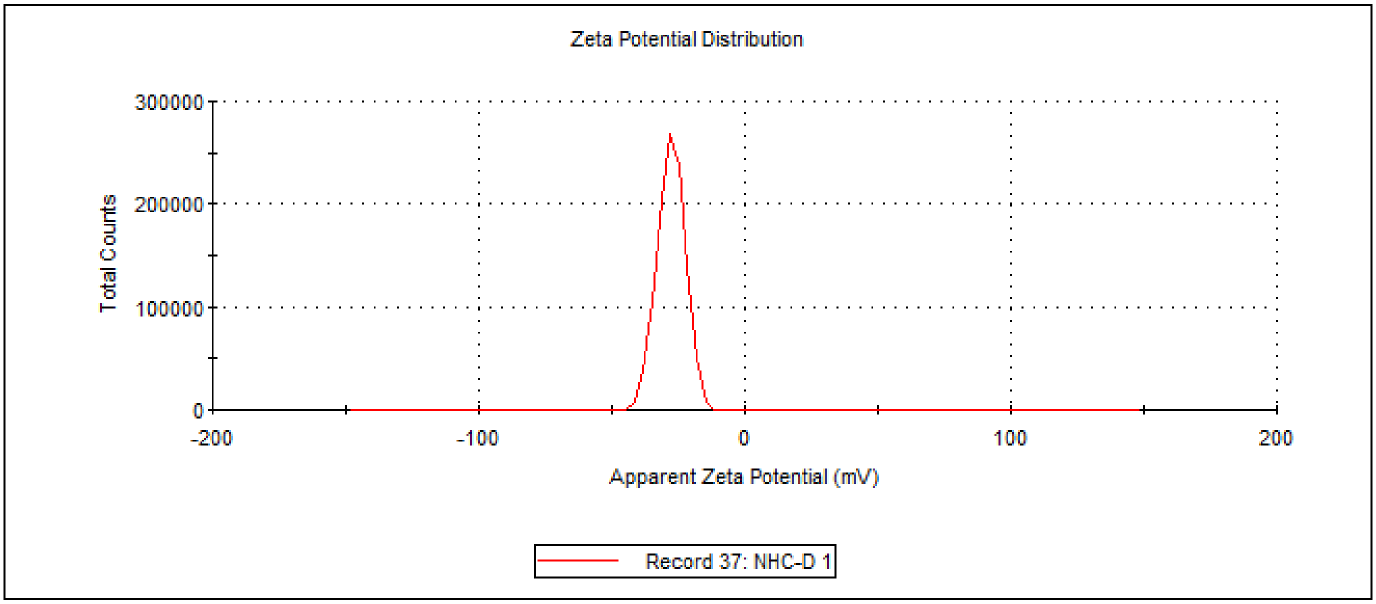


**Fig. S33.** Zeta potential measurement of top-down synthesized $^{RO}$NHC$^{Et}$-AuNPs.

## S.3.20. **NMR Spectra**

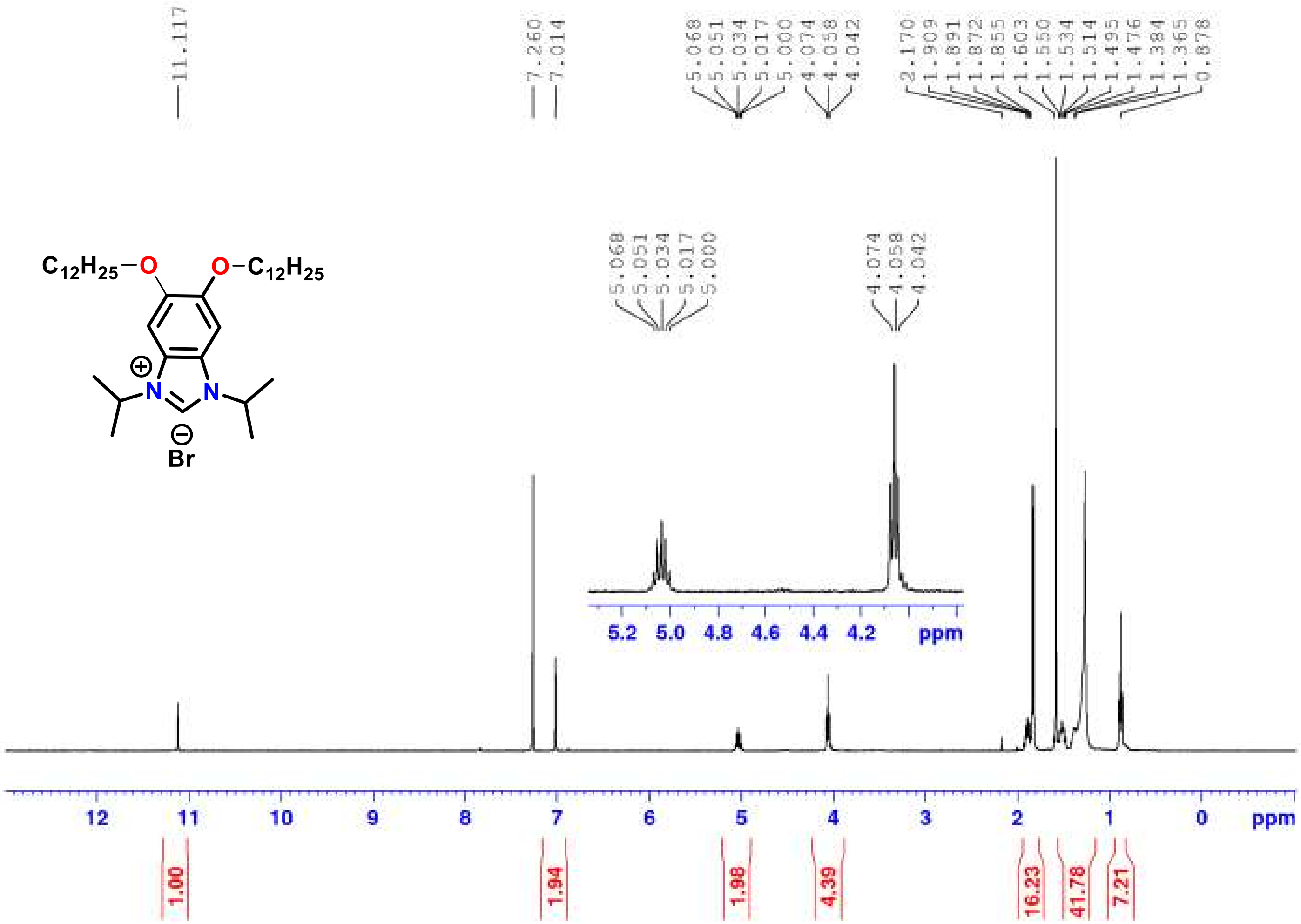


**Fig. S34.** $^{1}$H NMR (500 MHz; $CDCl_3$) spectrum of 5,6-bis(dodecyloxy)-1,3-diisopropylbenzimidazolium bromide.

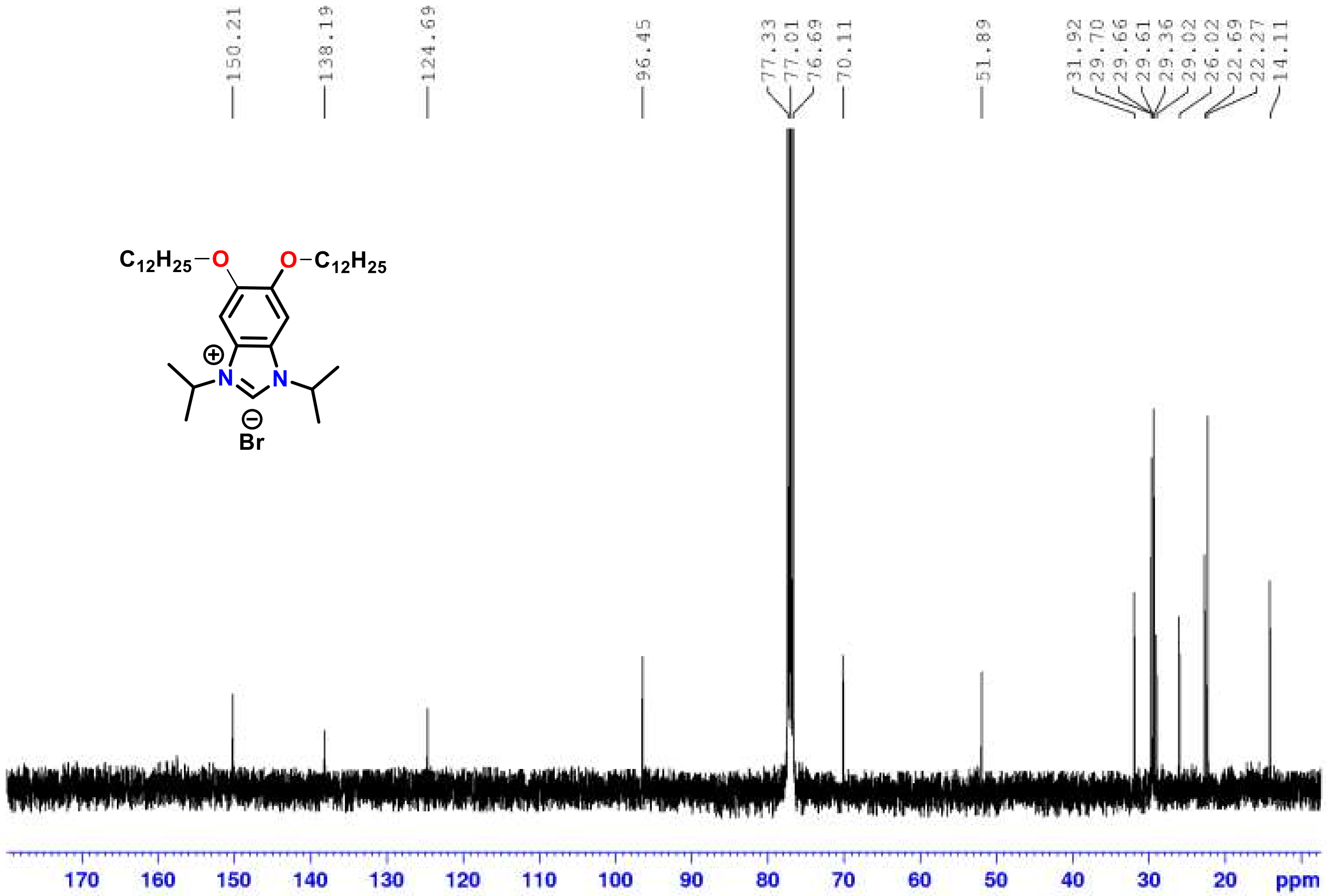


**Fig. S35.** $^{13}$C NMR (126 MHz; $CDCl_3$) spectrum of 5,6-bis(dodecyloxy)-1,3-diisopropylbenzimidazolium bromide.

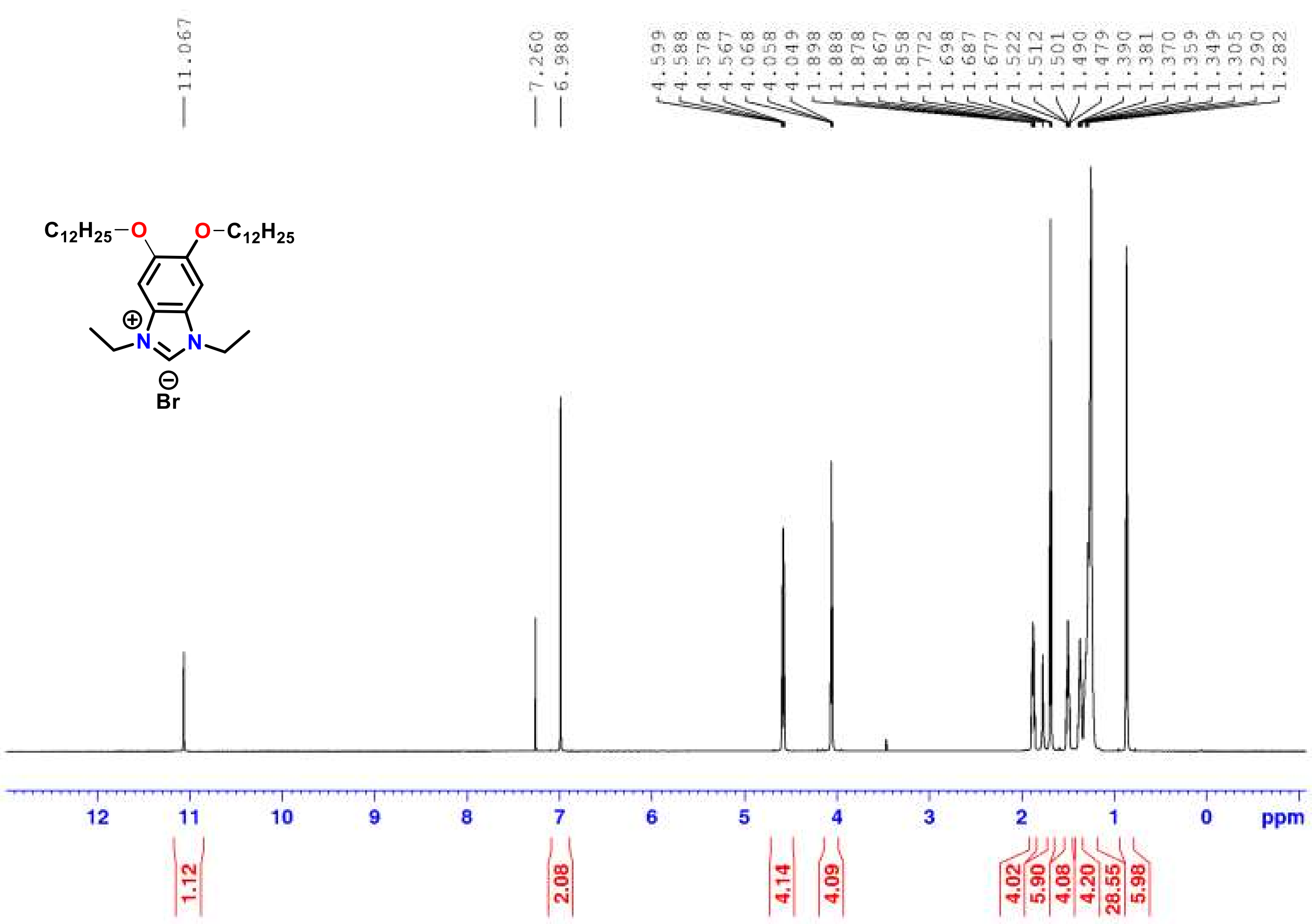


**Fig. S36.** $^{1}H$ NMR (500 MHz; $CDCl_3$) spectrum of 5,6-bis(dodecyloxy)-1,3-diethylbenzimidazolium bromide.

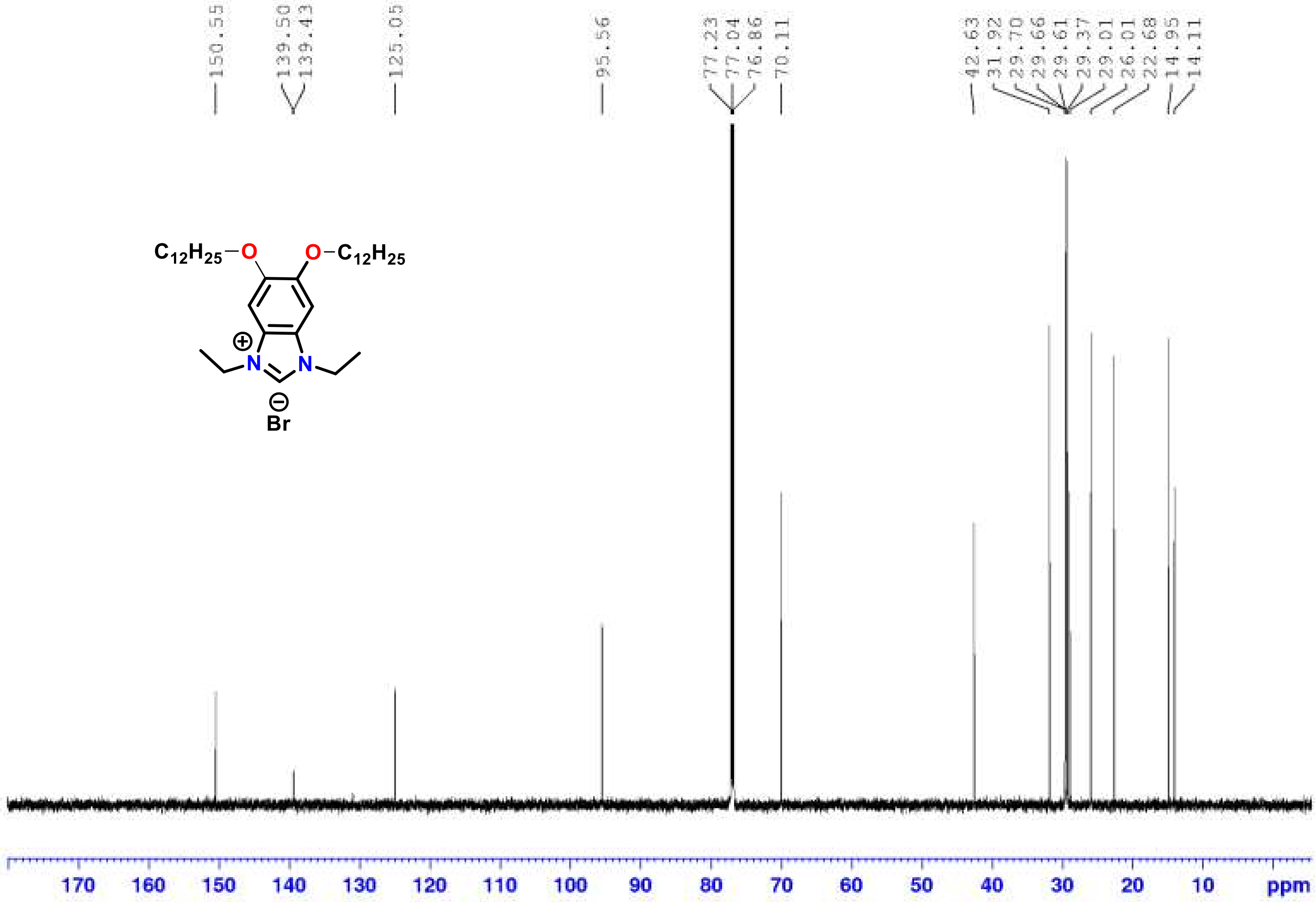


**Fig. S37.** $^{13}C$ NMR (126 MHz; $CDCl_3$) spectrum of 5,6-bis(dodecyloxy)-1,3-diethylbenzimidazolium bromide.

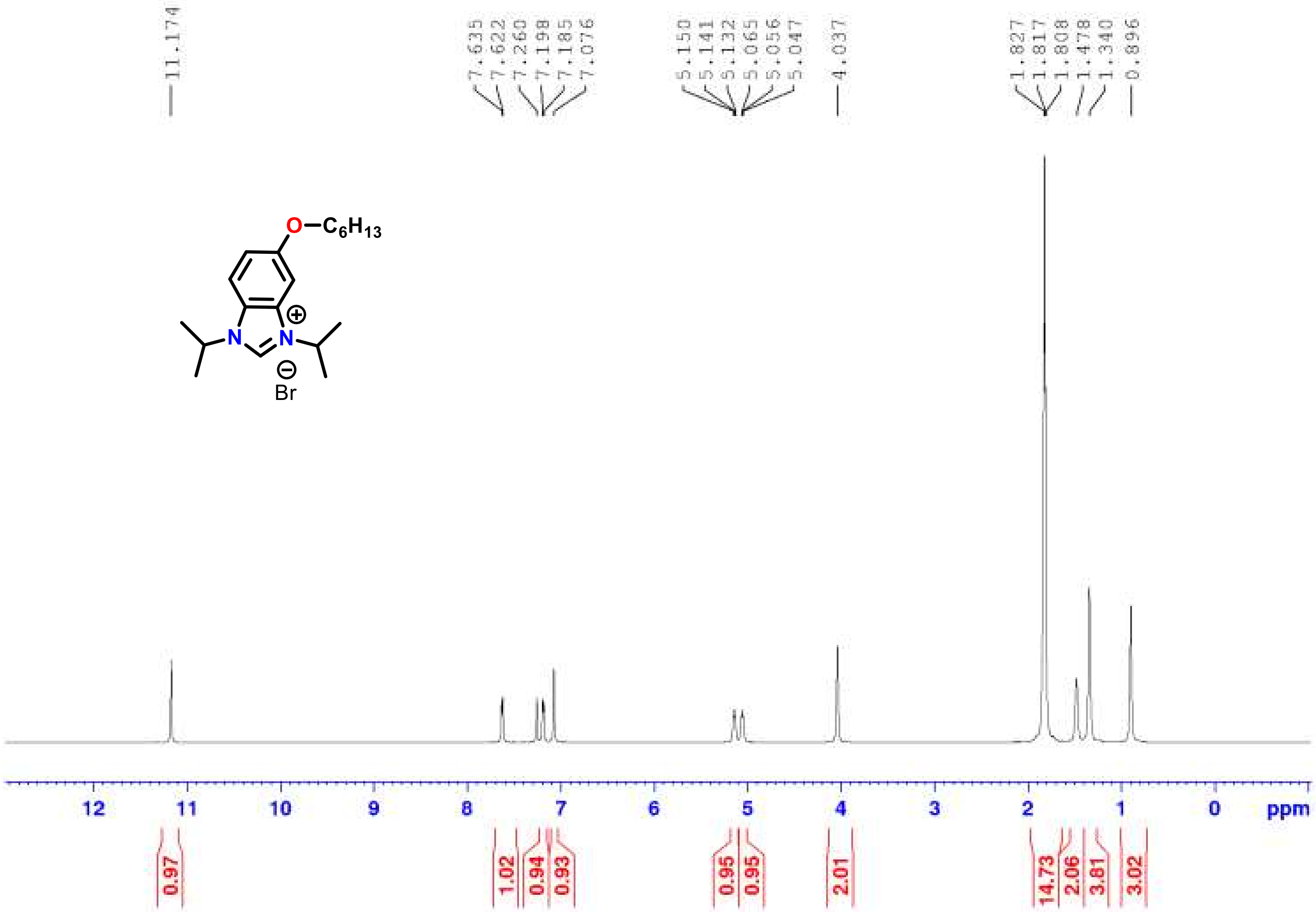


**Fig. S38.** $^{1}$H NMR (500 MHz; $CDCl_3$) spectrum of 1,3-diisopropyl-5-(hexyloxy)-1*H*-benzo[*d*]imidazol-3-ium bromide.

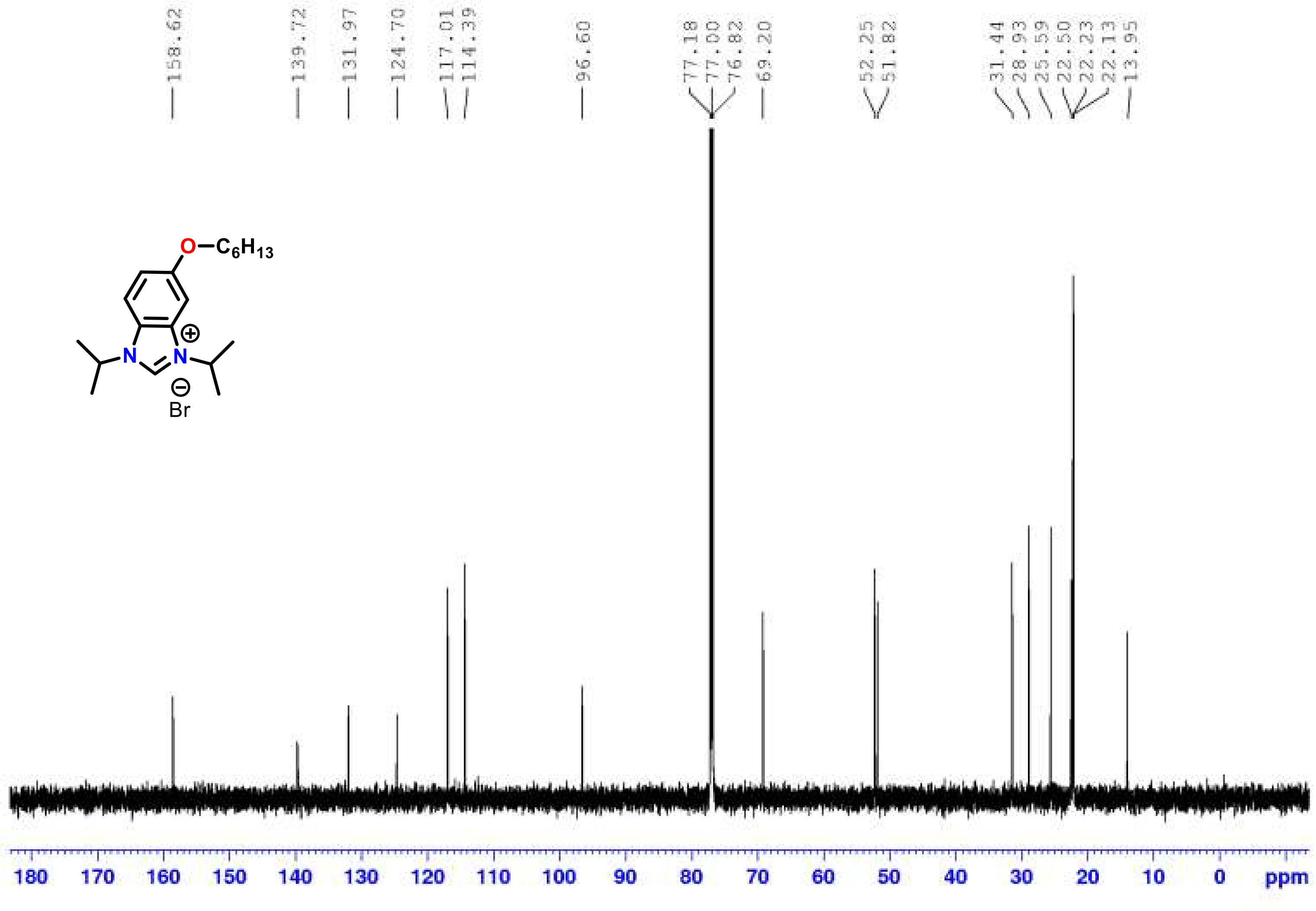


**Fig. S39.** $^{13}$C NMR (126 MHz; $CDCl_3$) spectrum of 1,3-diisopropyl-5-(hexyloxy)-1*H*-benzo[*d*]imidazol-3-ium bromide.

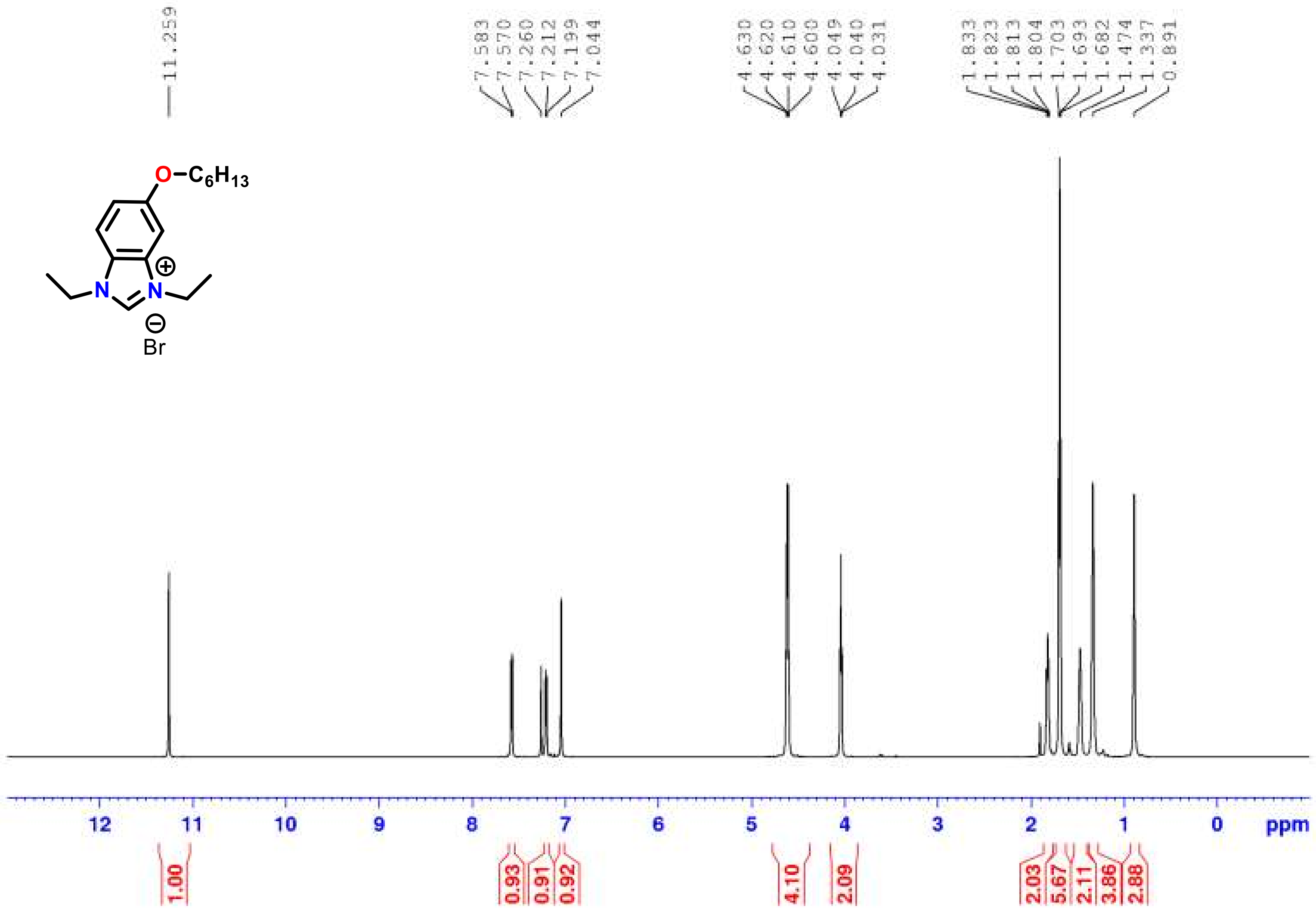

**Fig. S40.** $^{1}$H NMR (500 MHz; $CDCl_3$) spectrum of 1,3-diethyl-5-(hexyloxy)-1*H*-benzo[*d*]imidazol-3-ium bromide.

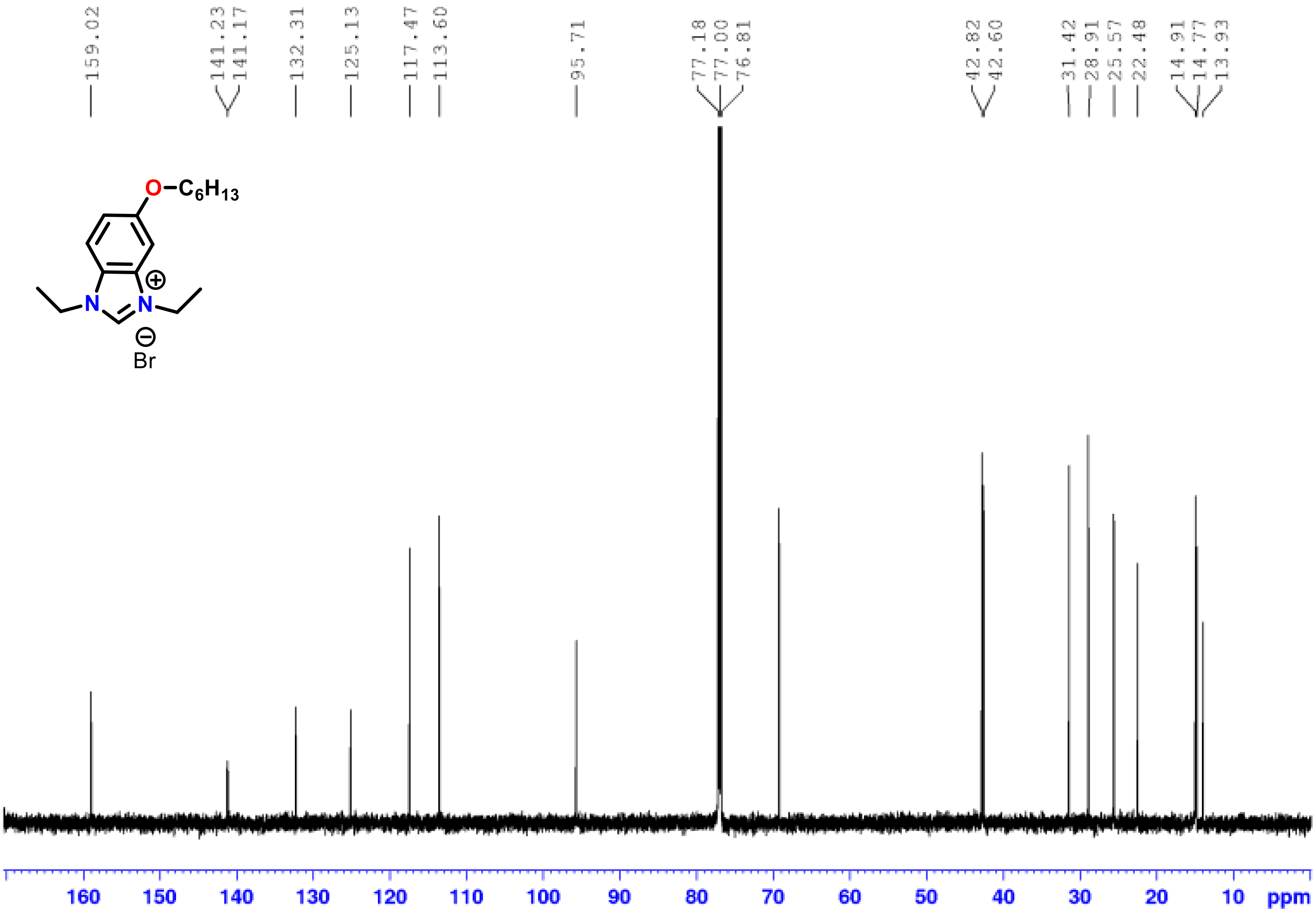


**Fig. S41.** $^{13}C$ NMR (126 MHz; $CDCl_3$) spectrum of 1,3-diethyl-5-(hexyloxy)-1*H*-benzo[*d*]imidazol-3-ium bromide.

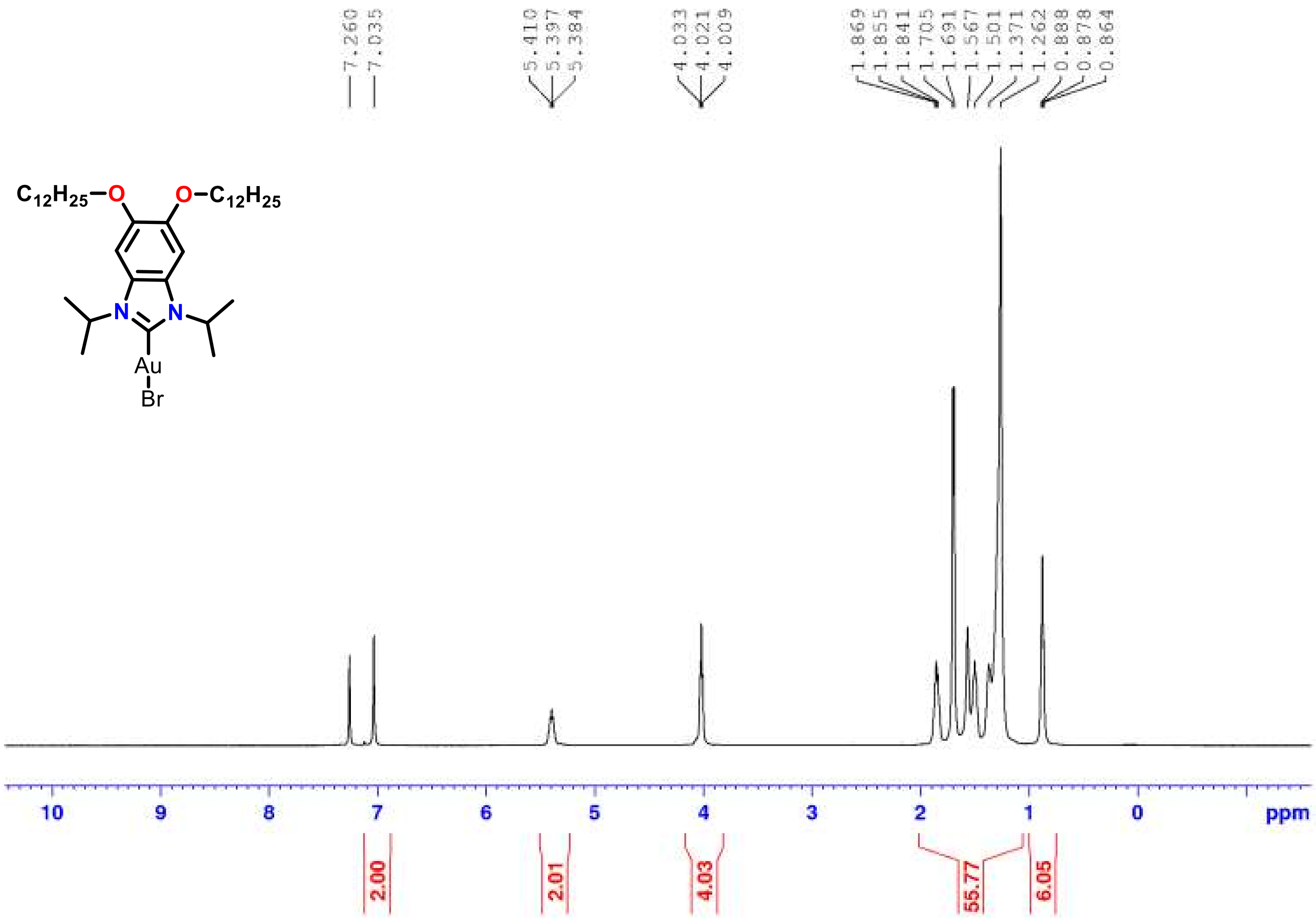


**Fig. S42.** $^1$H NMR (500 MHz; $CDCl_3$) spectrum of **$^{(RO)2}$NHC$^{iPr}$-AuBr**.

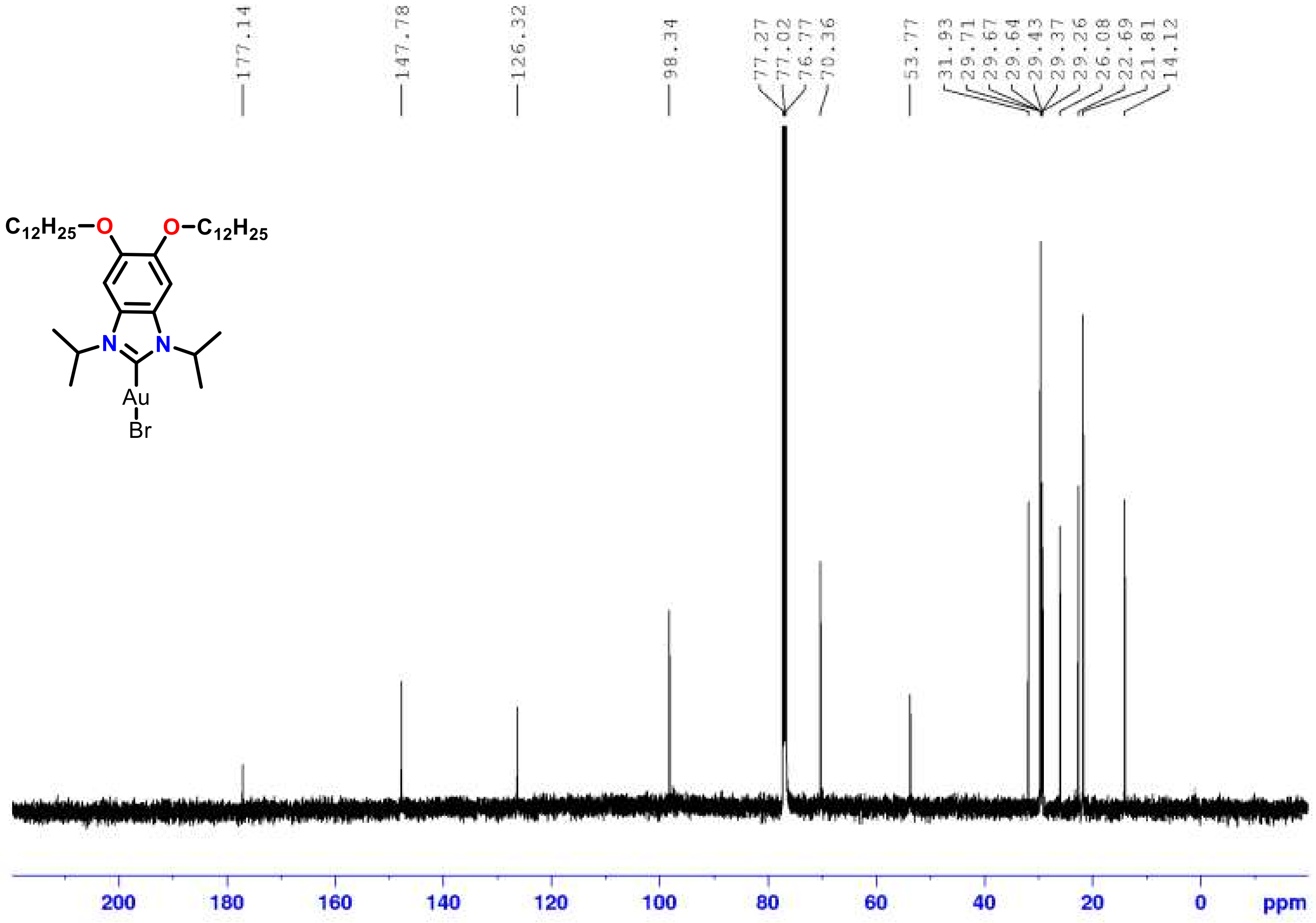


**Fig. S43.** $^{13}$C NMR (126 MHz; $CDCl_3$) spectrum of **$^{(RO)2}$NHC$^{iPr}$-AuBr**.

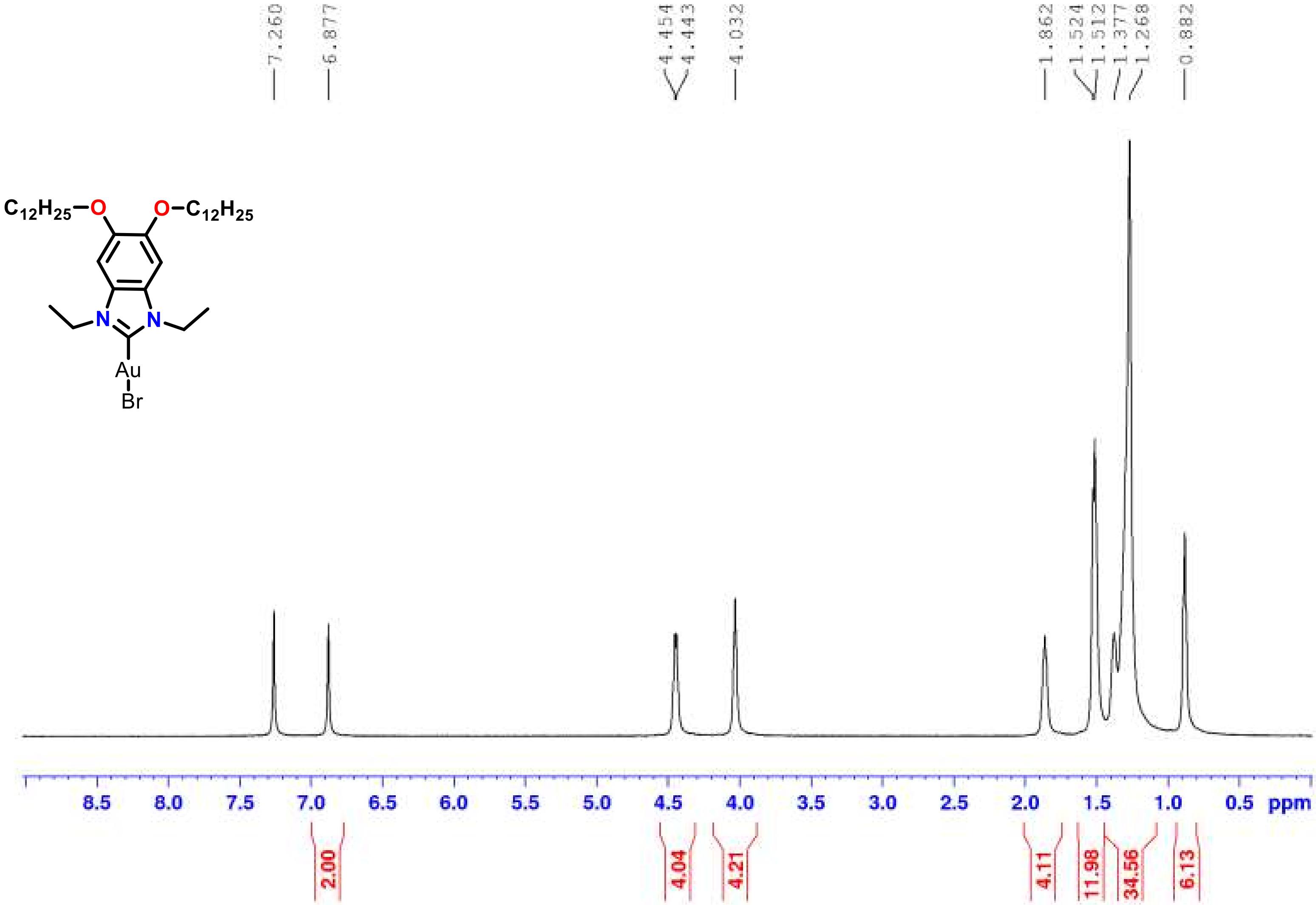


**Fig. S44.** $^{1}$H NMR (500 MHz; $CDCl_3$) spectrum of **$^{(RO)2}$NHC$^{Et}$-AuBr**.

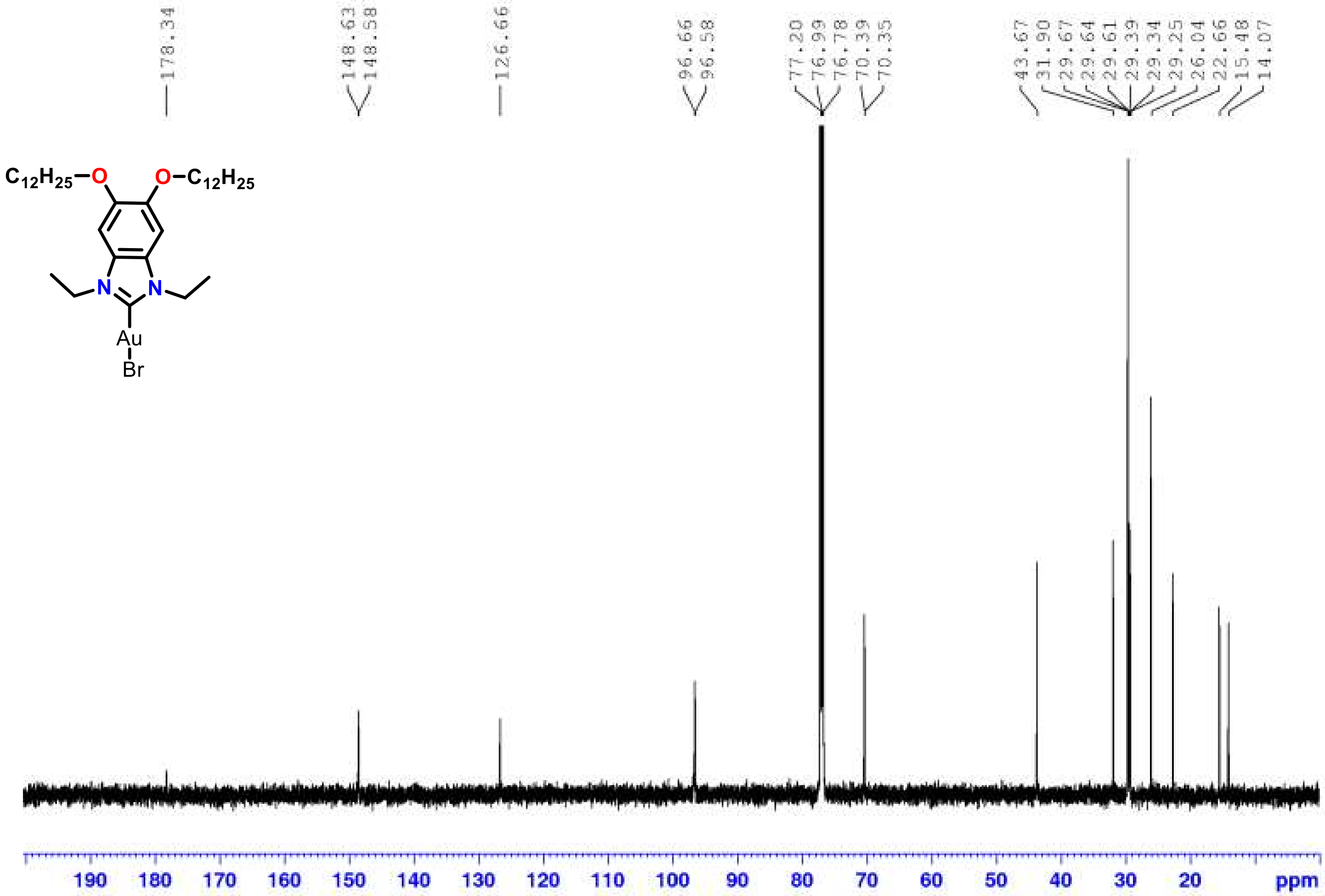


**Fig. S45.** $^{13}$C NMR (126 MHz; $CDCl_3$) spectrum of **$^{(RO)2}$NHC$^{Et}$-AuBr**.

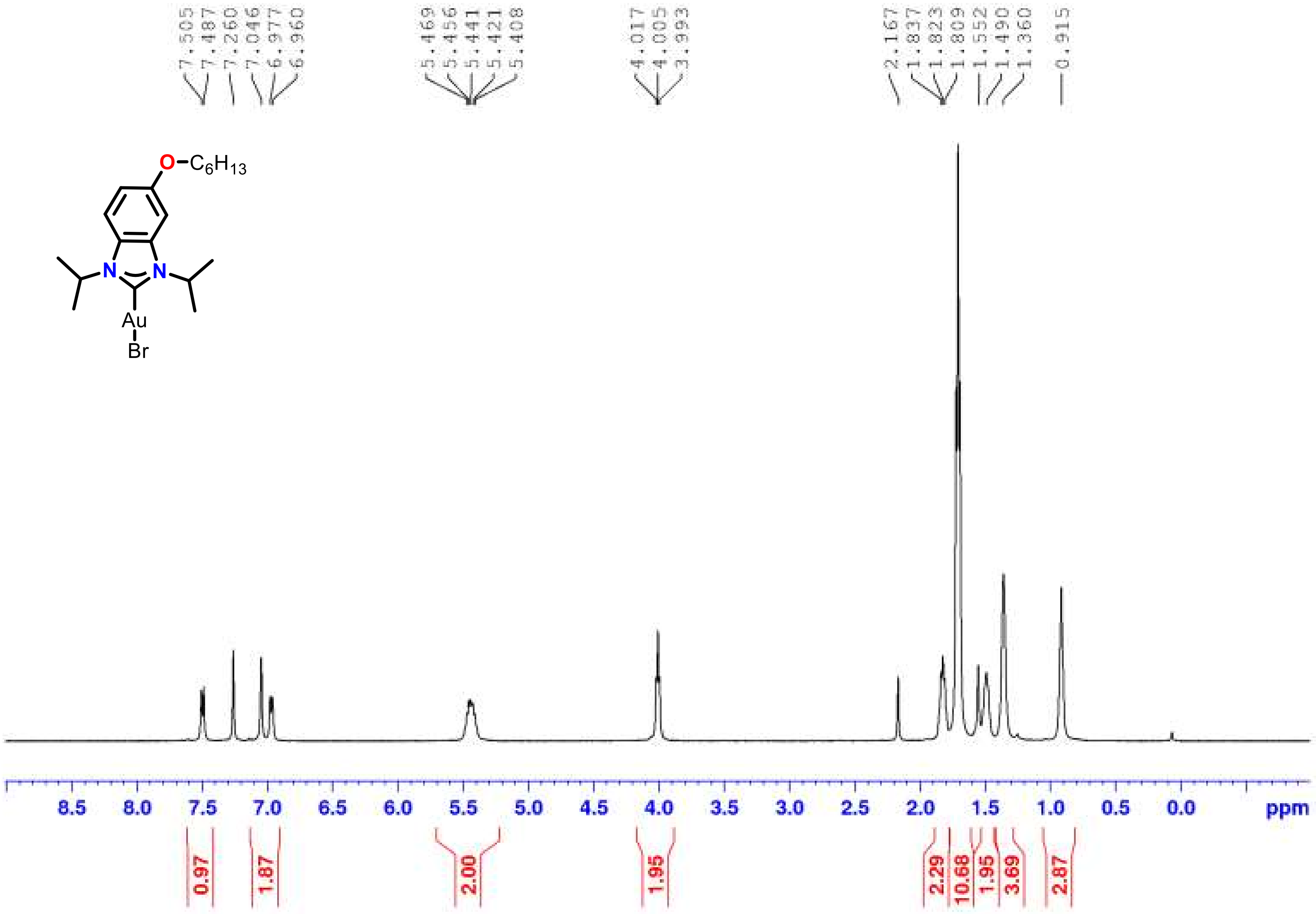


**Fig. S46.** $^{1}$H NMR (500 MHz; $CDCl_3$) spectrum of **$^{RO}$NHC$^{iPr}$-AuBr**.

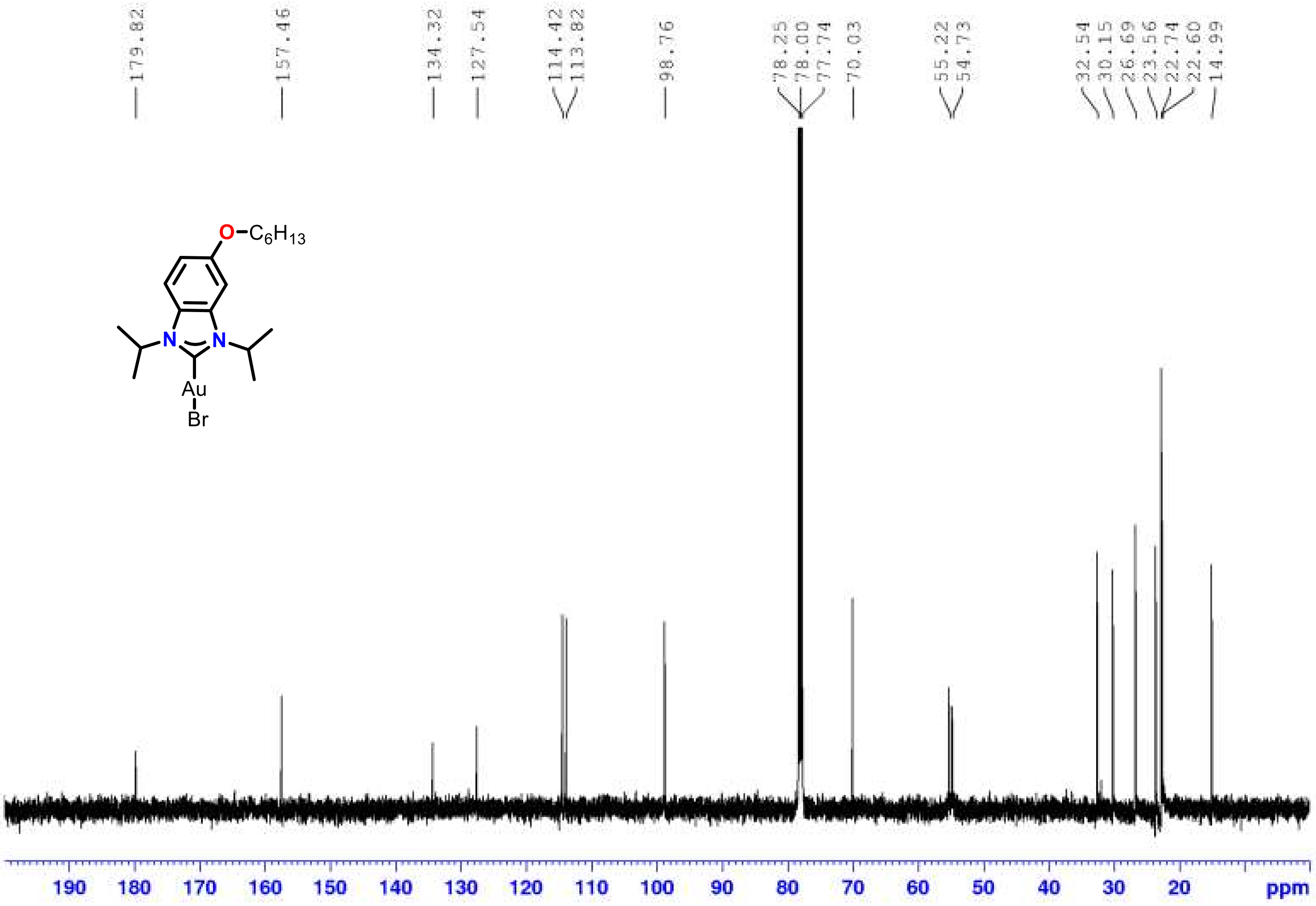


**Fig. S47.** $^{13}$C NMR (126 MHz; $CDCl_3$) spectrum of **$^{RO}$NHC$^{iPr}$-AuBr**.

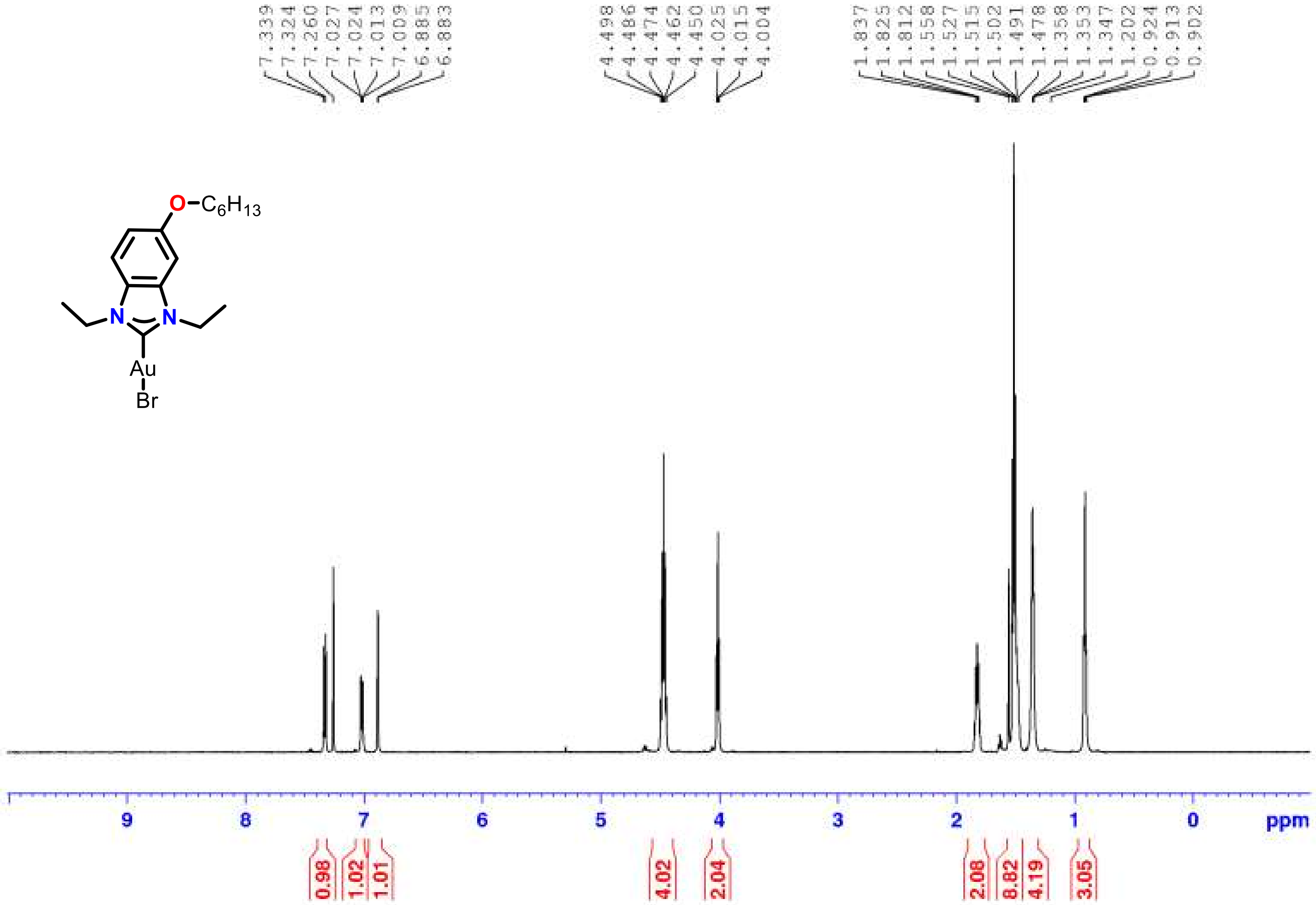


**Fig. S48.** $^{1}$H NMR (500 MHz; $CDCl_3$) spectrum of **$^{RO}$NHC$^{Et}$-AuBr**.

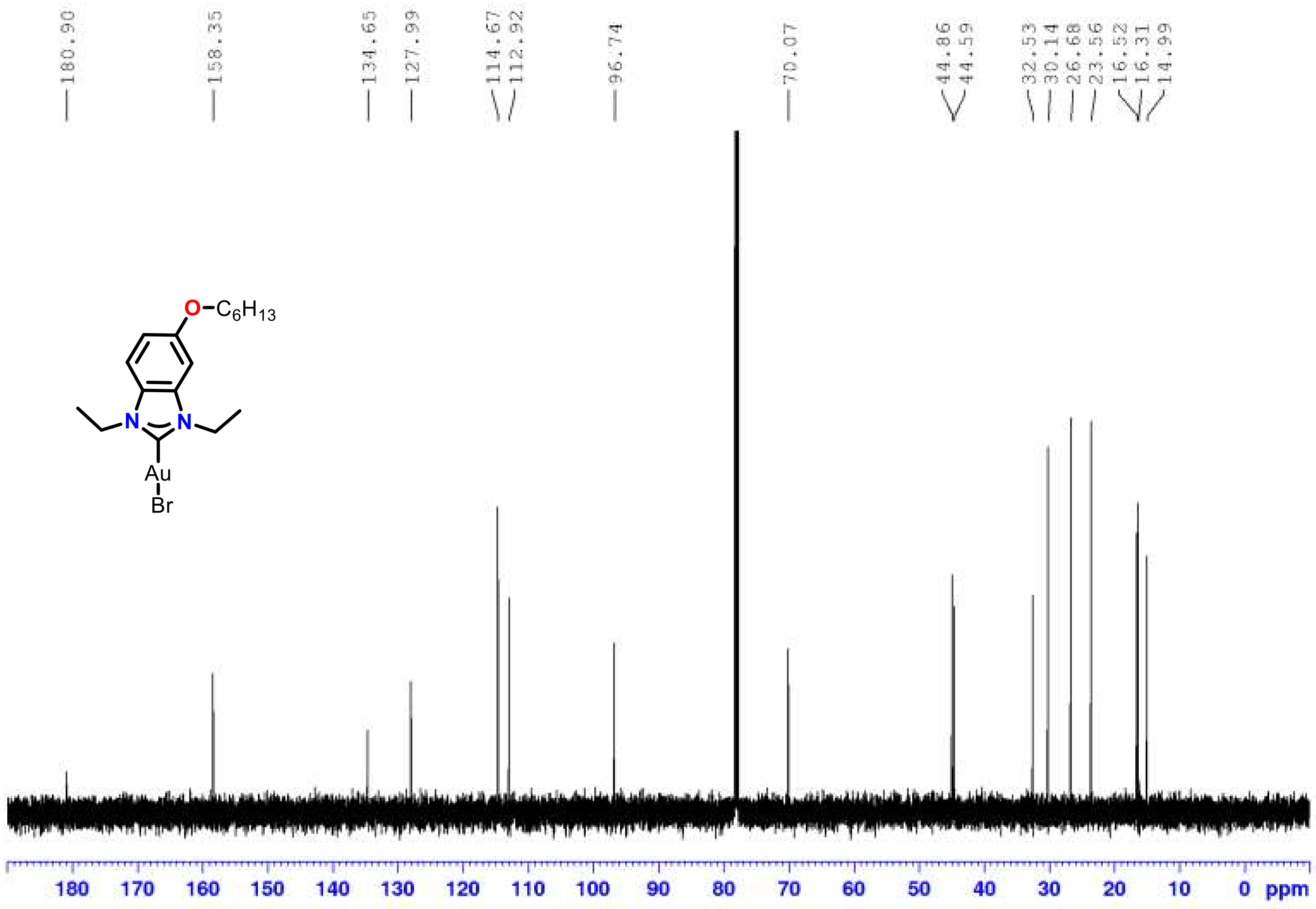


**Fig. S49.** $^{13}$C NMR (126 MHz; $CDCl_3$) spectrum of **$^{RO}$NHC$^{Et}$-AuBr**.

## S.3.21. Bond Lengths and Bond Angles of NHC gold complexes

**Table 2.** Bond Lengths for $^{(RO)2}$NHC$^{iPr}$-AuBr.

| Atom | Atom | Length/Å | Atom | Atom | Length/Å |
|---|---|---|---|---|---|
| Au | Br | 2.391(3) | C32 | C33 | 1.47(4) |
| Au | C31 | 2.00(2) | C32 | C34 | 1.43(4) |
| O1 | C25 | 1.389(18) | C13 | C14 | 1.525(18) |
| O1 | C1 | 1.43(3) | C1 | C2 | 1.540(18) |
| C31 | N1 | 1.38(3) | C8 | C9 | 1.535(18) |
| C31 | N2 | 1.28(3) | C8 | C7 | 1.532(18) |
| O2 | C26 | 1.365(18) | C15 | C14 | 1.534(18) |
| O2 | C13 | 1.509(17) | C15 | C16 | 1.536(18) |
| N1 | C29 | 1.37(2) | C19 | C18 | 1.523(18) |
| N1 | C35 | 1.47(3) | C19 | C20 | 1.526(18) |
| N2 | C28 | 1.40(2) | C21 | C20 | 1.521(18) |
| N2 | C32 | 1.49(3) | C21 | C22 | 1.539(18) |
| C27 | C28 | 1.3900 | C17 | C16 | 1.520(18) |
| C27 | C26 | 1.3900 | C17 | C18 | 1.517(18) |
| C28 | C29 | 1.3900 | C9 | C10 | 1.521(18) |
| C29 | C30 | 1.3900 | C5 | C4 | 1.531(18) |
| C30 | C25 | 1.3900 | C5 | C6 | 1.523(18) |
| C25 | C26 | 1.3900 | C10 | C11 | 1.536(18) |
| C23 | C22 | 1.537(18) | C7 | C6 | 1.534(18) |
| C23 | C24 | 1.536(18) | C3 | C4 | 1.526(18) |

## S.3.21. Bond Lengths and Bond Angles of NHC gold complexes

**Table 2.** Bond Lengths for $^{(RO)2}$NHC$^{iPr}$-AuBr.

| Atom | Atom | Length/Å | Atom | Atom | Length/Å |
|---|---|---|---|---|---|
| C36 | C35 | 1.53(4) | C3 | C2 | 1.535(18) |
| C37 | C35 | 1.57(4) | C12 | C11 | 1.513(18) |

**Table 3**. Bond Angles for **$^{(RO)2}$NHC$^{iPr}$-AuBr.**

| Atom | Atom | Atom | Angle/° | Atom | Atom | Atom | Angle/° |
|---|---|---|---|---|---|---|---|
| C31 | Au | Br | 179.2(6) | C33 | C32 | N2 | 111(2) |
| C25 | O1 | C1 | 112.2(17) | C34 | C32 | N2 | 111(3) |
| N1 | C31 | Au | 121.1(16) | C34 | C32 | C33 | 117(3) |
| N2 | C31 | Au | 129.4(16) | O2 | C13 | C14 | 102.2(17) |
| N2 | C31 | N1 | 108(2) | N1 | C35 | C36 | 112(2) |
| C26 | O2 | C13 | 118.3(15) | N1 | C35 | C37 | 111(2) |
| C31 | N1 | C35 | 125(2) | C36 | C35 | C37 | 112(2) |
| C29 | N1 | C31 | 107.8(17) | O1 | C1 | C2 | 106(2) |
| C29 | N1 | C35 | 127(2) | C7 | C8 | C9 | 112(2) |
| C31 | N2 | C28 | 111.4(18) | C14 | C15 | C16 | 108.7(19) |
| C31 | N2 | C32 | 129(2) | C18 | C19 | C20 | 115(2) |
| C28 | N2 | C32 | 119(2) | C20 | C21 | C22 | 112.2(19) |
| C28 | C27 | C26 | 120.0 | C18 | C17 | C16 | 112(2) |
| C27 | C28 | N2 | 135.2(13) | C13 | C14 | C15 | 107.9(19) |
| C27 | C28 | C29 | 120.0 | C10 | C9 | C8 | 112(2) |

**Table 3**. Bond Angles for $^{(RO)2}$**NHC$^{iPr}$-AuBr.**

| Atom | Atom | Atom | Angle/° | Atom | Atom | Atom | Angle/° |
|---|---|---|---|---|---|---|---|
| C29 | C28 | N2 | 104.8(13) | C17 | C16 | C15 | 111.6(19) |
| N1 | C29 | C28 | 107.5(13) | C17 | C18 | C19 | 115(2) |
| N1 | C29 | C30 | 132.5(13) | C6 | C5 | C4 | 114(2) |
| C30 | C29 | C28 | 120.0 | C21 | C20 | C19 | 113(2) |
| C29 | C30 | C25 | 120.0 | C9 | C10 | C11 | 107(2) |
| O1 | C25 | C30 | 121.0(12) | C8 | C7 | C6 | 112(2) |
| O1 | C25 | C26 | 118.7(12) | C23 | C22 | C21 | 112.9(19) |
| C26 | C25 | C30 | 120.0 | C4 | C3 | C2 | 112(2) |
| O2 | C26 | C27 | 124.8(12) | C3 | C4 | C5 | 113(2) |
| O2 | C26 | C25 | 115.2(12) | C5 | C6 | C7 | 110(2) |
| C25 | C26 | C27 | 120.0 | C3 | C2 | C1 | 113(2) |
| C24 | C23 | C22 | 115(2) | C12 | C11 | C10 | 110(2) |

**Table 4**. Bond Lengths for $^{(RO)2}$**NHC$^{Et}$-AuBr.**

| Atom | Atom | Length/Å | Atom | Atom | Length/Å |
|---|---|---|---|---|---|
| Au1 | Br1 | 2.3780(12) | C13 | C14 | 1.38(2) |
| Au1 | C1 | 1.962(10) | C14 | C15 | 1.56(3) |
| O1 | C4 | 1.361(11) | C15 | C16 | 1.37(2) |
| O1 | C12 | 1.437(12) | C16 | C17 | 1.49(2) |
| O2 | C5 | 1.372(11) | C17 | C18 | 1.506(16) |
| O2 | C24 | 1.40(2) | C18 | C19 | 1.52(2) |
| N1 | C1 | 1.365(12) | C19 | C20 | 1.48(2) |
| N1 | C2 | 1.394(12) | C20 | C21 | 1.47(2) |

**Table 4**. Bond Lengths for $^{(RO)2}$**NHC$^{Et}$-AuBr.**

| Atom | Atom | Length/Å | Atom | Atom | Length/Å |
|---|---|---|---|---|---|
| N1 | C8 | 1.470(12) | C21 | C22 | 1.499(13) |
| N2 | C1 | 1.361(11) | C22 | C23 | 1.419(15) |
| N2 | C7 | 1.384(12) | C24 | C25 | 1.543(16) |
| N2 | C10 | 1.498(13) | C25 | C26 | 1.469(17) |
| C2 | C3 | 1.364(13) | C26 | C27 | 1.556(17) |
| C2 | C7 | 1.360(11) | C27 | C28 | 1.487(17) |
| C3 | C4 | 1.361(13) | C28 | C29 | 1.519(16) |
| C4 | C5 | 1.433(12) | C29 | C30 | 1.495(17) |
| C5 | C6 | 1.353(13) | C30 | C31 | 1.550(16) |
| C6 | C7 | 1.408(13) | C31 | C33 | 1.508(17) |
| C8 | C9 | 1.455(19) | C32 | C33 | 1.531(16) |
| C10 | C11 | 1.40(2) | C32 | C34 | 1.499(17) |
| C12 | C13 | 1.51(2) | C34 | C35 | 1.480(16) |

**Table 5**. Bond Angles for $^{(RO)2}$**NHC$^{Et}$-AuBr**.

| Atom | Atom | Atom | Angle/° | Atom | Atom | Atom | Angle/° |
|---|---|---|---|---|---|---|---|
| C1 | Au1 | Br1 | 179.5(3) | C2 | C7 | C6 | 121.0(9) |
| C4 | O1 | C12 | 116.9(8) | C9 | C8 | N1 | 109.8(12) |
| C5 | O2 | C24 | 116.0(11) | C11 | C10 | N2 | 106.8(15) |
| C1 | N1 | C2 | 110.9(7) | O1 | C12 | C13 | 106.6(11) |
| C1 | N1 | C8 | 123.1(8) | C14 | C13 | C12 | 120.6(19) |
| C2 | N1 | C8 | 125.9(8) | C13 | C14 | C15 | 116(2) |
| C1 | N2 | C7 | 110.2(7) | C16 | C15 | C14 | 120(2) |

**Table 5**. Bond Angles for **$^{(RO)2}$NHC$^{Et}$-AuBr**.

| Atom | Atom | Atom | Angle/° | Atom | Atom | Atom | Angle/° |
|---|---|---|---|---|---|---|---|
| C1 | N2 | C10 | 123.7(8) | C15 | C16 | C17 | 115.8(19) |
| C7 | N2 | C10 | 125.7(8) | C16 | C17 | C18 | 115.4(18) |
| N1 | C1 | Au1 | 127.3(6) | C17 | C18 | C19 | 106.4(17) |
| N2 | C1 | Au1 | 127.5(7) | C20 | C19 | C18 | 108.8(17) |
| N2 | C1 | N1 | 105.1(8) | C21 | C20 | C19 | 109.9(18) |
| C3 | C2 | N1 | 131.6(8) | C20 | C21 | C22 | 106.2(17) |
| C7 | C2 | N1 | 105.8(8) | C23 | C22 | C21 | 105.4(17) |
| C7 | C2 | C3 | 122.5(9) | O2 | C24 | C25 | 106.3(16) |
| C4 | C3 | C2 | 118.2(8) | C26 | C25 | C24 | 113(2) |
| O1 | C4 | C3 | 126.3(9) | C25 | C26 | C27 | 109.9(19) |
| O1 | C4 | C5 | 113.7(9) | C28 | C27 | C26 | 115(2) |
| C3 | C4 | C5 | 119.9(9) | C27 | C28 | C29 | 110.1(19) |
| O2 | C5 | C4 | 114.2(9) | C30 | C29 | C28 | 110.4(19) |
| C6 | C5 | O2 | 124.4(8) | C29 | C30 | C31 | 103.9(19) |
| C6 | C5 | C4 | 121.4(9) | C33 | C31 | C30 | 108.2(19) |
| C5 | C6 | C7 | 116.9(8) | C34 | C32 | C33 | 95.9(18) |
| N2 | C7 | C6 | 131.1(8) | C31 | C33 | C32 | 105.0(18) |
| C2 | C7 | N2 | 107.9(8) | C35 | C34 | C32 | 95.9(18) |

# S4. References


1 L. S. Kassel, *J. Chem. Phys.*, 1936, **4**, 276–282.

2 J. P. Perdew, *Phys. Rev. B*, 1986, **33**, 8822–8824.

3 A. Allouche, *J. Comput. Chem.*, 2012, **32**, 174–182.

4 E. Van Lenthe, R. Van Leeuwen, E. J. Baerends and J. G. Snijders, *Int. J. Quantum Chem.*, 1996, **57**, 281–293.

5 L. Jensen, L. L. Zhao, J. Autschbach and G. C. Schatz, *J. Chem. Phys.*, DOI:10.1063/1.2046670.

6 *PyMOL Mol. Graph. Syst. Schrödinger, LLC.*

7 J. Holz, M. Ayerbe García, W. Frey, F. Krupp and R. Peters, *Dalt. Trans.*, 2018, **47**, 3880–3905.

8 A. Nezamzadeh, E. Kaur, M. D. Aloisio, D. A. R. Nanan, Y. S. Hedberg, C. M. Crudden and M. C. Biesinger, DOI:10.1021/acs.jpcc.5c03111.

9 C. M. Crudden, J. H. Horton, I. I. Ebralidze, O. V. Zenkina, A. B. McLean, B. Drevniok, Z. She, H. B. Kraatz, N. J. Mosey, T. Seki, E. C. Keske, J. D. Leake, A. Rousina-Webb and G. Wu, *Nat. Chem.*, 2014, **6**, 409–414.

10 L. Kolářová, L. Prokeš, L. Kučera, A. Hampl, E. Peňa-Méndez, P. Vaňhara and J. Havel, *J. Am. Soc. Mass Spectrom.*, 2017, **28**, 419–427.

11 N. L. Dominique, A. Chandran, I. M. Jensen, D. M. Jenkins and J. P. Camden, *Chem. - A Eur. J.*, DOI:10.1002/chem.202303681.

12 T. B. Demille, R. A. Hughes, N. Dominique, J. E. Olson, S. Rouvimov, J. P. Camden and S. Neretina, *Nanoscale*, 2020, **12**, 16489–16500.

13 J. E. Olson, A. S. Braegelman, L. Zou, M. J. Webber and J. P. Camden, *Appl. Spectrosc.*, 2020, **74**, 1374–1383.

14 F. Benz, R. Chikkaraddy, A. Salmon, H. Ohadi, B. De Nijs, J. Mertens, C. Carnegie, R. W. Bowman and J. J. Baumberg, *J. Phys. Chem. Lett.*, 2016, **7**, 2264–2269.

15 J. P. Camden, J. A. Dieringer, Y. Wang, D. J. Masiello, L. D. Marks, G. C. Schatz and R. P. Van Duyne, *J. Am. Chem. Soc.*, 2008, **130**, 12616–12617.

16 J. K. Daniels and G. Chumanov, *J. Phys. Chem. B*, 2005, **109**, 17936–17942.

17 J. F. DeJesus, L. M. Sherman, D. J. Yohannan, J. C. Becca, S. L. Strausser, L. F. P. Karger, L. Jensen, D. M. Jenkins and J. P. Camden, *Angew. Chemie*, 2020, **132**, 7655–7660.

18 P. C. Lee and D. Meisel, *J. Phys. Chem.*, 1982, **86**, 3391–3395.

19 N. L. Dominique, I. M. Jensen, G. Kaur, C. Q. Kotseos, W. C. Boggess, D. M. Jenkins and J. P. Camden, *Angew. Chemie - Int. Ed.*, 2023, **62**, 1–7.

20 N. L. Dominique, R. Chen, A. V. B. Santos, S. L. Strausser, T. Rauch, C. Q. Kotseos, W. C. Boggess, L. Jensen, D. M. Jenkins and J. P. Camden, *Inorg. Chem. Front.*, 2022, **9**, 6279–6287.

21 C. M. Crudden, J. H. Horton, M. R. Narouz, Z. Li, C. A. Smith, K. Munro, C. J. Baddeley, C. R. Larrea, B. Drevniok, B. Thanabalasingam, A. B. McLean, O. V. Zenkina, I. I. Ebralidze, Z. She, H. B. Kraatz, N. J. Mosey, L. N. Saunders and A. Yagi, *Nat. Commun.*, 2016, **7**, 1–7.

22 L. M. Sherman, S. L. Strausser, R. K. Borsari, D. M. Jenkins and J. P. Camden, *Langmuir*, 2021, **37**, 5864–5871.

23 I. M. Jensen, V. Clark, H. L. Kirby, N. Arroyo-Currás and D. M. Jenkins, *Mater. Adv.*, 2024, **5**, 7052–7060.

24 G. Kaur, N. L. Dominique, G. Hu, P. Nalaoh, R. L. Thimes, S. L. Strausser, L. Jensen, J. P. Camden and D. M. Jenkins, *Inorg. Chem. Front.*, 2023, **10**, 6282–6293.

25 J. F. Dejesus, M. J. Trujillo, J. P. Camden and D. M. Jenkins, *J. Am. Chem. Soc.*, 2018, **140**, 1247–1250.

26 R. L. Thimes, A. V. B. Santos, R. Chen, G. Kaur, L. Jensen, D. M. Jenkins and J. P. Camden, *J. Phys. Chem. Lett.*, 2023, **14**, 4219–4224.

27 M. A. Pellitero, I. M. Jensen, N. L. Dominique, L. C. Ekowo, J. P. Camden, D. M. Jenkins and N. Arroyo-Currás, *ACS Appl. Mater. Interfaces*, 2023, **15**, 35701–35709.

28 R. W. Y. Man, C. H. Li, M. W. A. MacLean, O. V. Zenkina, M. T. Zamora, L. N. Saunders, A. Rousina-Webb, M. Nambo and C. M. Crudden, *J. Am. Chem. Soc.*, 2018, **140**, 1576–1579.
